# Transcriptome analysis and molecular mechanism of linseed (*Linum usitatissimum* L.) drought tolerance under repeated drought using single-molecule long-read sequencing

**DOI:** 10.1186/s12864-021-07416-5

**Published:** 2021-02-09

**Authors:** Wei Wang, Lei Wang, Ling Wang, Meilian Tan, Collins O. Ogutu, Ziyan Yin, Jian Zhou, Jiaomei Wang, Lijun Wang, Xingchu Yan

**Affiliations:** 1 Key Laboratory of Biology and Genetic Improvement of Oil Crops of Ministry of Agriculture and Rural Affairs, Oil Crops Research Institute of Chinese Academy of Agricultural Science, Wuhan, 430062 China; 2 CAS Key Laboratory of Plant Germplasm Enhancement and Specicalty Agriculature, Wuhan Botanical Garden, The Innovative Academy of Science Design, Chinese Academy of Sciences, Wuhan, 430074 China; 3 Wuhan Igenebook Biotechnology Co.,Ltd, Wuhan, 430075 China

**Keywords:** Transcriptome, Linseed, Repeated drought, SMRT, Transcription factors

## Abstract

**Background:**

Oil flax (linseed, *Linum usitatissimum* L.) is one of the most important oil crops., However, the increases in drought resulting from climate change have dramatically reduces linseed yield and quality, but very little is known about how linseed coordinates the expression of drought resistance gene in response to different level of drought stress (DS) on the genome-wide level.

**Results:**

To explore the linseed transcriptional response of DS and repeated drought (RD) stress, we determined the drought tolerance of different linseed varieties. Then we performed full-length transcriptome sequencing of drought-resistant variety (Z141) and drought-sensitive variety (NY-17) under DS and RD stress at the seedling stage using single-molecule real-time sequencing and RNA-sequencing. Gene Ontology (GO) and reduce and visualize GO (REVIGO) enrichment analysis showed that upregulated genes of Z141 were enriched in more functional pathways related to plant drought tolerance than those of NY-17 were under DS. In addition, 4436 linseed transcription factors were identified, and 1190 were responsive to stress treatments. Moreover, protein-protein interaction (PPI) network analysis showed that the proline biosynthesis pathway interacts with stress response genes through RAD50 (DNA repair protein 50) interacting protein 1 (RIN-1). Finally, proline biosynthesis and DNA repair structural gene expression patterns were verified by RT- PCR.

**Conclusions:**

The drought tolerance of Z141 may be related to its upregulation of drought tolerance genes under DS. Proline may play an important role in linseed drought tolerance by maintaining cell osmotic and protecting DNA from ROS damage. In summary, this study provides a new perspective to understand the drought adaptability of linseed.

**Supplementary Information:**

The online version contains supplementary material available at 10.1186/s12864-021-07416-5.

## Background

Drought stress (DS) is the most prevalent environmental factor limiting crop productivity and can directly result in an average yield loss of more than 50%, and global climate change is increasing the frequency of severe drought conditions [1]. Drought is expected to cause serious plant growth problems for more than 50% of arable land by 2050 [2]. DS affects crop water potential and turgor, e.g., reduces leaf expansion and promotes leaf senescence and abscission, which interfere with normal functions and change physiological and morphological traits in crops [3]. In addition, DS directly and indirectly, inhibits crop photosynthesis and leads to slow crop growth, yield loss, and even death.

Unlike animals, plants cannot simply uproot and move. Therefore, plants have evolved a series of special mechanisms to resist the damage caused by DS. A series of drought tolerance genes involved in the abscisic acid (ABA), proline, glycine-betaine, and sorbitol pathways upregulated by DS in wheat [4]. Similarly, tolerant maize varieties exhibited more drastic changes in global gene expression than susceptible varieties which correlated with different physiological mechanisms of adaptation to drought [5]. In addition, transgenic maize with enhanced *ZmVPP1* expression demonstrated improved drought tolerance which was attributed to enhanced photosynthetic efficiency and root development [6]. Despite recent advances, the mechanisms by which plants resist DS are still unclear.

Oil flax (*Linum usitatissimum* L.) also as known as linseed, is one of important oil crop in the world. It contains unsaturated fatty acids and plant hormones that are beneficial in the human body. Among them, α-linolenic acid (ALA) and secoisolariciresinol diglucoside (SDG) have been proven to promote nervous system development and significantly reduce breast cancer risk, respectively [7–10]. Furthermore, linseed is a fairly hardy species and has a higher level of drought tolerance than many other food crops. Therefore, it is widely grown in the western and northwestern provinces in China, such as Gansu and Inner Mongolia, which experience the highest drought frequency and longest drought in East Asia [11]. Nonetheless, DS still represents a major limit to linseed production [12]. Since 1995, when long-term traditional breeding programs to enhance linseed stress tolerance and improve crop yield under periodic drought, transgenic linseed plants have been obtained for enhancing tolerance to DS [13, 14]. Some transgenic linseed plants have been obtained for enhancing tolerance to drought stress [15]. Despite recent advances in linseed drought tolerance, how it functions is another open question.

PacBio’s SMRT (single-molecule real-time) sequencing (PacBio, http://www.pacificbiosciences.com/) provides is third-generation sequencing platform that is widely used for long-reads genome sequencing [16]. Due to its ability to obtain full-length transcripts without assembly, this method can provide direct comprehensive analysis of splice isoforms of each gene and improve annotation of existing gene models. SMRT sequencing is an ideal method for plant genome research due to the highly repetitive nature plant genomes compared to vertebrate genomes [17–19]. Recently, Li et al. (2017) used Iso-Seq to analyse full-length (FL) splice isoforms in strawberry, suggesting its suitability in uncovering the mechanism of drought tolerance in linseed [20].

Since the response of plants to DS is very complex, the physiological and transcription responses of leaves and roots to DS are almost completely different [21, 22]. In this study, we analysed and discussed the transcription data of only the aboveground parts to focus on determining the molecular mechanism underlying their response to DS. The first identified variation in drought tolerance of linseed varieties NY-17and Z141, was determined by combining SMRT sequencing and short-read next generation sequencing to generate a more complete FL linseed transcriptome. In addition, comprehensive candidate gene identification was conducted for; DS, re-watering (RW), and repeated drought (RD) conditions, and analysis of expression patterns for homologous genes in linseed was performed under different drought conditions.

## Results

### Determination of drought tolerance in linseed varieties

**Fig. 1:**
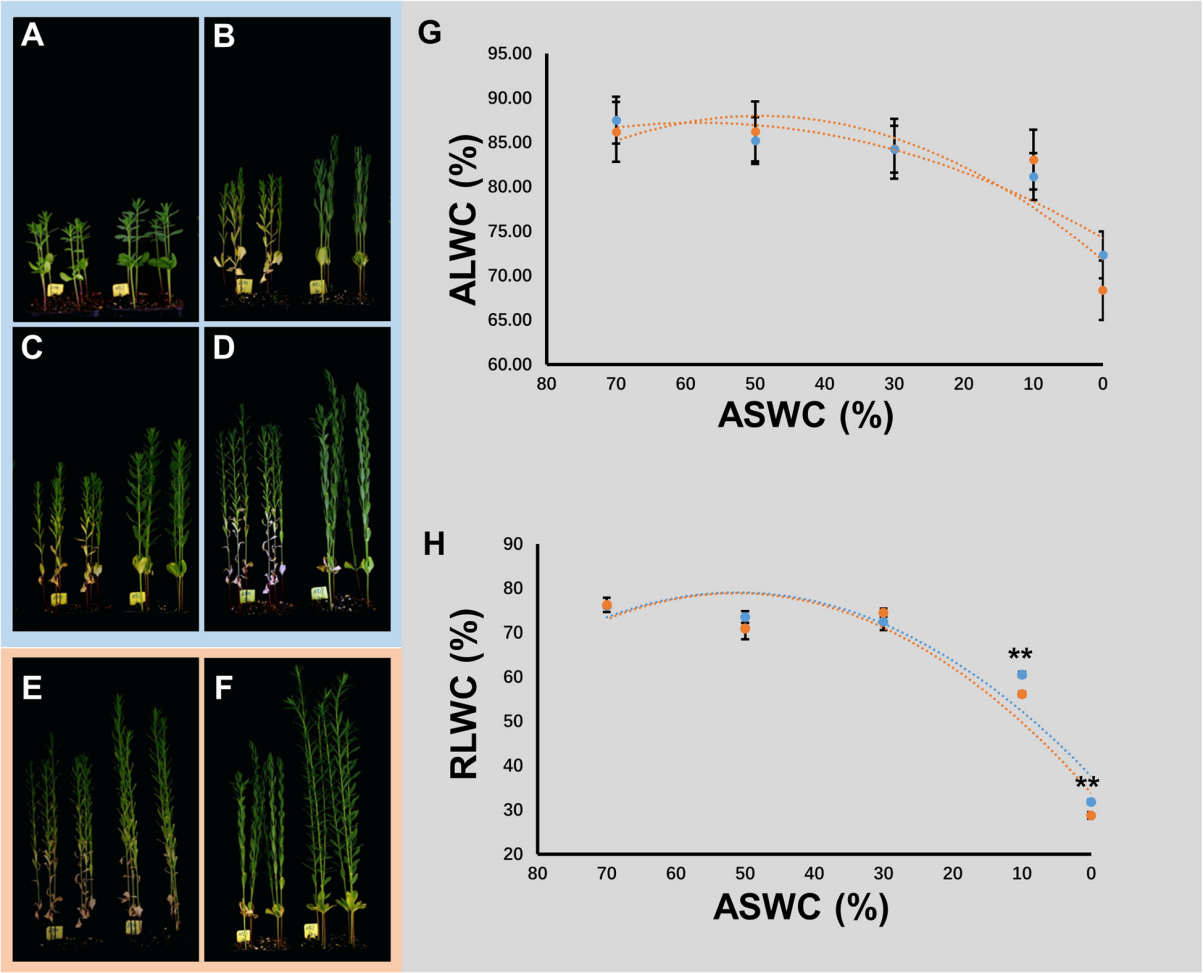
Identification of linseed drought tolerance. **a**-**d** Z141 (left) and NY-17 (right) phenotype differences under normal water content (CK), drought stress (DS), re-watering (RW), and repeated drought (RD) respectively. **e**, **f** Z141 and NY-17 phenotypic differences between drought stress (left) and controls (right). **g**, **h** Z141 and NY-17 ALWC and RLWC with means and SEs (*n* = 3) respectively. The abscissa indicates ASWC, and the ordinates indicate ALWC (**g**) and RLWC (**h**). Blue dots indicate Z141, and orange dots indicate NY-17. **, *p* < 0.01, see Table 1 for ANOVA and Table S2 and Table S3 for a summary of these drought tolerance-related traits

In this study, we measured three drought-tolerance related phenotypic traits of Z141 and NY-17 (Additional file 1). Z141 consistently performed better than NY-17 under DS (Fig. 1a-d). In addition, under DS, Z141 had a lower plant height and biomass reduction rate compared than NY-17 under DS (Fig. 1e, f; Additional file 2). The biomass reduction rate under DS was 30 and 46% in Z141 and NY-17 respectively. The relative leaf water content (RLWC) of Z141 was significantly higher than that of NY-17, suggesting that Z141 leaves can retain more water under drought stress. (Fig. 1g, h; Additional file 3).

Two-way ANOVA results showed significant effects of the different varieties and different drought level treatments and their effects on plant height, biomass ALWC and RLWC (Table 1). By comparing the phenotypes of Z141 and NY-17 under drought stress, it is found that the drought-tolerant of Z141 was stronger than that of NY-17. Therefore, we reveal the molecular mechanism difference between Z141 and NY-17 in response to drought stress using single-molecule long-read transcriptome sequencing.

**Table 1:** Two-way ANOVAs to test the effects of different drought stress (Fixed effect), two linseed biotypes (random effects), and their interaction on plant height, biomass, leaf absolute water content (LAWC) and leaf relative water content (LRWC)

Trait	Drought			Linseed			D x L		
	df	*F*	*p*	df	*F*	*p*	df	*F*	*p*
Plant height	1	709.13	0.000	1	479.90	0.000	1	26.36	0.001
Biomass	1	108.03	0.000	1	302.29	0.000	1	41.29	0.000
LAWC	1	314.44	0.000	1	1.27	0.292	1	36.88	0.000
LRWC	1	949.63	0.000	1	5.22	0.05	1	6.48	0.03

The drought (D) had two levels (drought stress or non-drought stress) and linseed biotype (L) had two levels too

### Analysis of the linseed transcriptome by PacBio Iso-Seq

Total RNA of Z141 and NY-17 was isolated from control, DS, RW and RD treatment groups and quality checked. A total of 16 RNA samples were sent to Wuhan Frasergen Bioinformatics Co.,Ltd. Genomic Service for sequencing using the PacBio Sequel platform. This platform can generate sufficiently long read lengths that cover the full length of most RNA transcripts, ensuring that accurate reconstructed FL splice variants are obtained. Over 2 million polymerase reads with a mean length of ~ 30,000 bp were generated after quality checking by Frasergen (Additional file 4). After processing raw data, we obtained more than 33 million filtered subreads with a mean length of ~ 2000 bp (Additional file 5). In addition, we obtained 1,599,415 circular consensus (CCS) reads, which included 1,293,134 FL reads (Additional file 6). De novo reconstruction of the transcriptome data was performed using RNA-Seq reads and publicly available flax sequences. To evaluate the density and length of isoforms, we compared the locus coverages of PacBio full-length and non-chimeric (FLNC) sequences and swine SSC 10.2 annotation. In the PacBio dataset, a total of 1,093,282 high-quality FLNC sequences covered 108,579 isoforms and were allocated to 28,686 loci (Additional file 7). Due to the high base error of SMRT sequencing, high-quality Illumina short reads were obtained using Prooveread software to correct the errors (Additional file 8). In this study, the pre- and post-correction FLNC sequences were aligned to the linseed genome sequence through GMAP, and finally, we obtained 1,093,282 high-quality FLNC sequences for further study (Additional file 9).

### Global comparisons of DS- and RD-related transcriptomes reveal gene expression and functional group differences

**Fig. 2:**
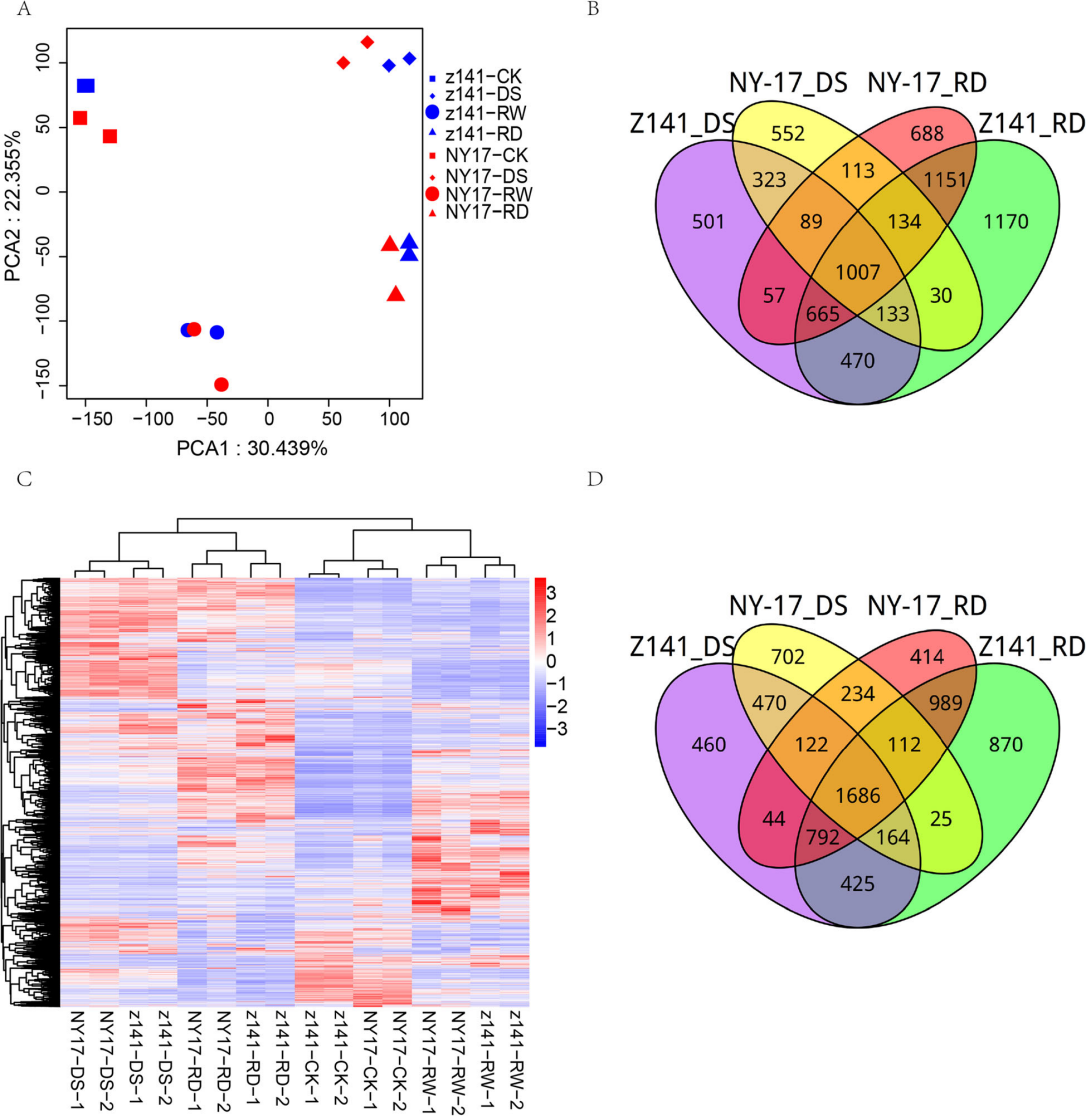
Comparative analysis of transcriptome profiles of linseed seedling leaves under DS and RD. **a** Principal component analysis (PCA) of mRNA populations from the control, DS, RW and RD groups. Each sample contained two replicates. Principal components (PCs) 1 and 2 account for 30 and 22% of the variance, respectively. The PCA plot shows four distinct groups of mRNA populations. Group I: Z141 CK (blue square) and NY-17 CK (red square); group II: Z141 DS (blue diamond) and NY-17 DS (red diamond); group III: Z141 RW (blue circle) and NY-17 RW (red circle) and group IV: Z141 RD (blue triangle) and NY-17 RD (red triangle). **b** Hierarchical clustering of DEGs exhibiting altered expression levels in response to CK, DS, RW and RD treatments. The colours in the scale (blue (low), white (medium) and red (high)) represent the normalized expression levels of differentially expressed DEGs. **c**, **d** Venn diagrams showing overlap of up- (**c**) or downregulated (**d**) genes in response to the four assayed abiotic stresses: Z141-DS (purple), NY-17-DS (yellow), Z141-RD (green) and NY-17-RD (red)

mRNA populations were compared using principal component analysis (PCA) to provide a framework for understanding how linseed genes are regulated to respond to DS. Transcriptomes of Z141 and NY-17 under DS, RW and RD were likely to share a great similarity in gene expression, with variations forming three groups that were separated far from the control (Fig. 2a). The transcriptomes of DS exhibited a distinct relationship from those of RD, suggesting that the gene expression in the transcriptome has a major shift between DS and RD.

Cluster analysis of differentially expressed genes (DEGs) further supported our observed results from PCA (Fig. 2b). The overlaps of up- and downregulated genes between Z141-RD and NY-17-RD was significantly higher than that between Z141-DS and Z141-RD, with 62.1% compared to 47.8% (upregulated) and 70.7% compared to 60.6% (downregulated) respectively (Fig. 2c, d). In addition, in Z141 and NY-17 approximately 52.2 and 65.6% of upregulated genes were responsive to only RD respectively, and 29.9 and 43.6% of upregulated genes were responsive to only DS (Additional file 10). Specifically, in Z141 and NY-17, 8005 (including 3245 for DS and 4760 for RD) and 6285 (including 2381 for DS and 3904 for RD) genes were upregulated under drought, respectively (Additional file 10). Approximately 9104 (including 4041 for DS and 5063 for RD) and 7908 (3515 for DS and 4393 for RD) genes were downregulated under drought in Z141 and NY-17 (Additional file 10). We also observed a higher proportion of stress-responsive genes under RD than that under DS. In this study, 2275 and 1343 genes were upregulated, and 3067 and 2154 were downregulated when Z141 and NY-17 were under DS, respectively. In total, 1007 and 1686 genes were significantly up- and downregulated when Z141 and NY-17 were under DS and RD (Fig. 2c, d). Taken together, these results suggest that the transcriptomes of DS and RD has fundamentally different.

**Fig. 3:**
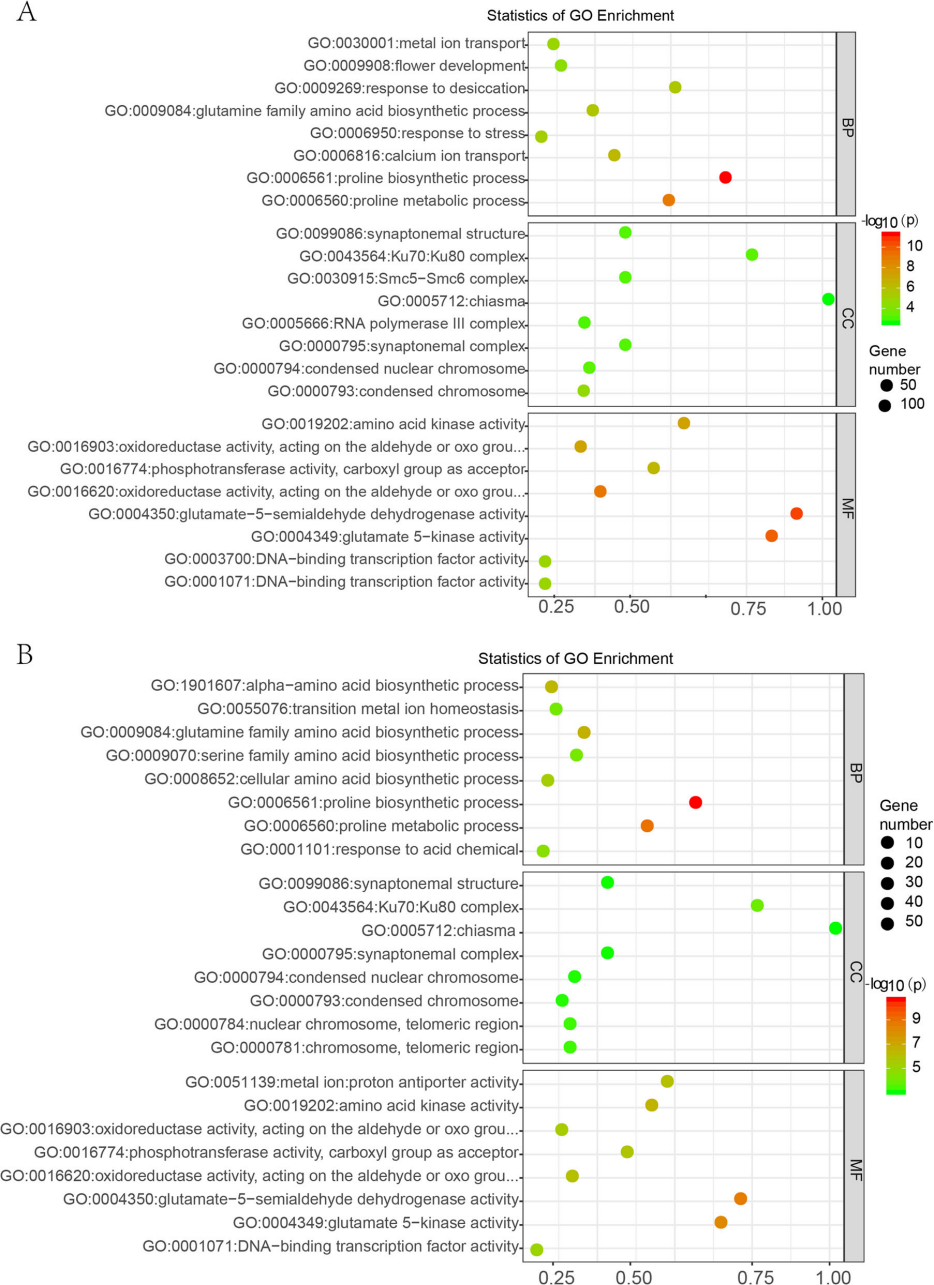
Bubble diagram showing the Gene Ontology (GO) classification of upregulated genes overlapping between DS and RD in Z141 or NY-17. GO terms of upregulated genes overlapping between DS and RD in Z141 (**a**) or in NY-17 (**b**). The three main GO categories are (from top to bottom): biological process, cellular component and molecular function

Gene Ontology (GO) enrichment analysis was conducted to examine the functional distribution of the DS-related candidate genes identified in our study. We performed GO enrichment analysis on 2275 and 1343 DEGs that both up-regulated under DS and RD stress in Z141 or NY-17 respectively (Additional file 11). A series of GO categories exhibited significantly higher enrichments in the overlapping or unique upregulated gene sets under DS and RD treatments compared to their levels in the control. The GO terms of upregulated genes overlapping between DS and RD in Z141 and NY-17 were mainly enriched in “proline biosynthetic process (GO: 0006561)” and “proline metabolic process (GO: 0006560)” (Fig. 3a, b). Moreover, except for amino acid biosynthesis and metabolism, abiotic stress-related GO terms e.g., “response to stress (GO: 0009650)” and “response to desiccation (GO: 0009269)”, exhibited significant enrichment among Z141 upregulated genes (Fig. 3a). Interestingly, GO terms related to flower development (GO: 0009908) were significantly enriched in only Z141 upregulated genes (Additional file 11, Fig. 3a). Precocious flowering might be an important drought avoidance mechanism for species preservation when plants under stress [23, 24]. Therefore, this result may indicates that the drought avoidance mechanism of Z141 was activated. DS inhibits plant photosynthesis. In this study, the GO terms of photosynthesis (GO: 0015979) were significantly enriched in downregulated genes in Z141 and NY-17 under DS and RD (Additional file 11). Proline accumulation is one of the striking metabolic responses of plants to drought stress, it contributes to the redox balance of cells under stressful conditions [25]. Our study showed that proline biosynthesis genes were significantly up-regulated in linseed under drought stress.

The difference in linseed gene regulation patterns under DS and RD, suggests that under repeated DS, linseed may have different molecular mechanisms for drought tolerance. In order to verify this hypothesis, we performed GO enrichment analysis on 970 and 2485 DEGs that were specifically up-regulated in Z141 under DS or RD stress. Of the stress responsive GO terms, two distinct functional categories of specific DS upregulated genes in Z141 exhibited significantly higher enrichments, namely methylation and negative regulation. The first group included “histone H3-K36 demethylation (GO: 0070544)” and “macromolecule methylation (GO: 0043414)”, whereas the second group included “negative regulation of biological process (GO: 0048519)” and “negative regulation of macromolecule metabolic process (GO: 0010605)” (Additional files 11 and 12). The GO terms of upregulated genes in Z141 under RD were mainly enriched in “fatty acid oxidation (GO: 0019395)”, “fatty acid biosynthetic process (GO: 0006633)”, “fatty acid m metabolic process (GO: 0006631)” and “lipid metabolic process (GO: 0006629)” (Additional file 11). The GO terms of genes downregulated in only Z141 under DS were mainly enriched in “carbohydrate metabolic process (GO: 0005975)”, “lignin biosynthetic process (GO: 0009809)” and “lignin metabolic process (GO: 0009808)”, whereas under RD, the GO terms of genes downregulated in only Z141 were mainly enriched in “amide biosynthetic process (GO: 0043604)” and “cellular amide metabolic process (GO: 0043603)” (Additional files 11 and 12). Overall, these functional categories indicated that epigenetic modifications might play a crucial role in the DS response process, although the exact functions of these genes remain to be elucidated. Meanwhile, DS may induce the Z141 to shift from vegetative growth to reproductive growth.

Under DS, 1038 DEGs were specifically up-regulated in NY-17, and their GO terms of genes were mainly enriched in RNA regulation, including “RNA modification (GO: 0009451)”, “RNA processing (GO: 0006396)” and “ncRNA processing (GO: 0034470)” (Additional file 11). There were 1525 DEGs specifically up-regulated under RD, and the GO terms of genes upregulated only under RD were mainly enriched in “transmembrane transport (GO: 0055085)” (Additional files 11 and 12). The GO terms of 1379 specifically down-regulated DEGs in NY-17 under DS were mainly enriched in flavonoid biosynthesis (GO: 0009813). Interestingly, more than 3000 DEGs were specifically down-regulated in NY-17 under RD stress, and the GO terms of genes were similar to those in Z141 and were mainly enriched in “amide biosynthetic process (GO: 0043604)” and “cellular amide metabolic process (GO: 0043603)” (Additional files 11 and 12).

### Comparison of Z141 and NY-17 transcriptomes reveals the molecular mechanism of linseed drought tolerance

**Fig. 4:**
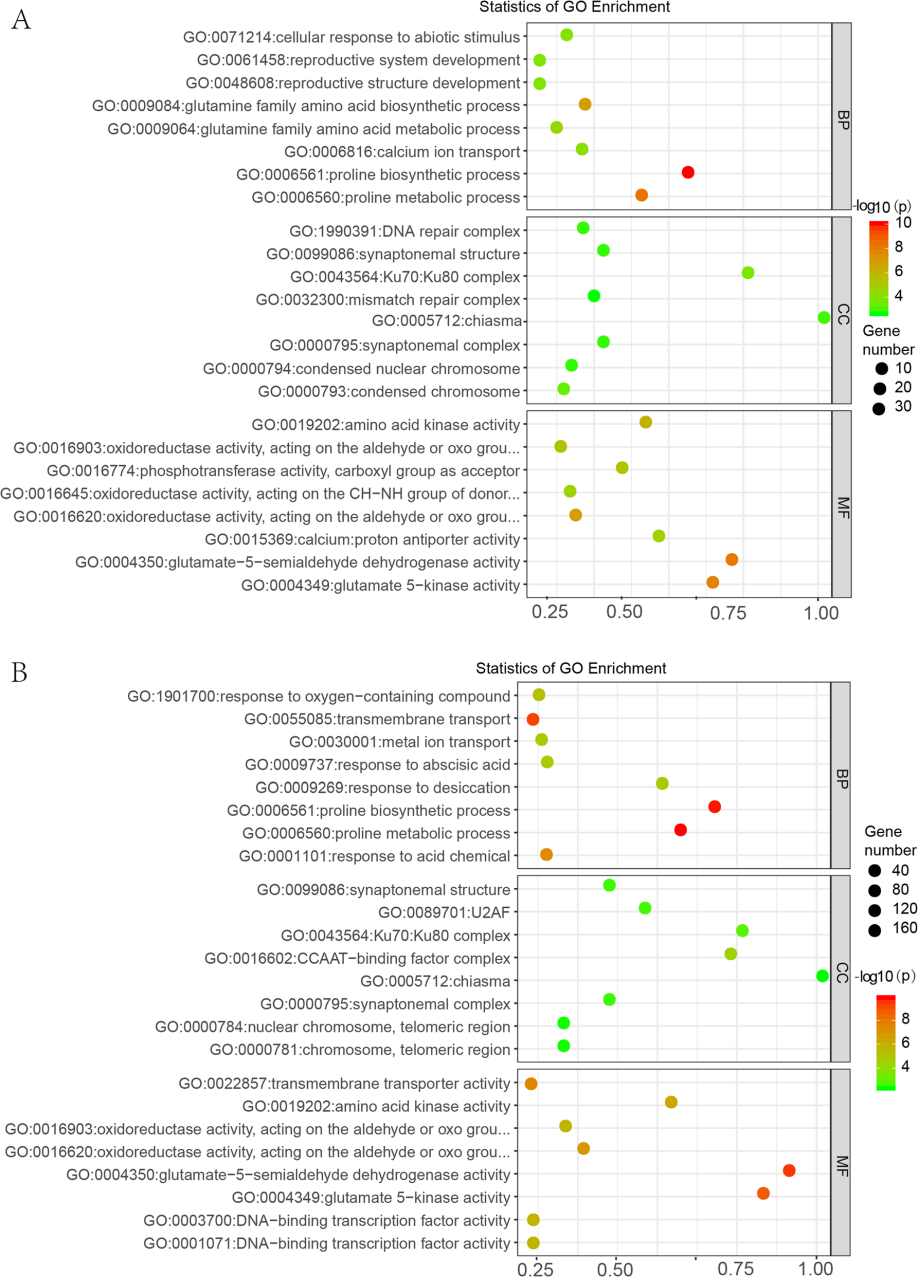
Bubble diagram showing the *p* value significance of enriched GO categories for Z141 and NY-17 overlapping upregulated genes in response to DS or RD. The GO terms of upregulated genes overlapping between Z141 and NY-17 under DS (**a**) or RD (**b**) treatment. Different colours indicate different functional groups

Although the transcriptomes of Z141 and NY-17 are very similar in overall gene expression, a set of stress-responsive genes exhibited altered expression patterns specific to Z141 or NY-17 under DS, indicating that genes of distinguished functional categories could impact the drought tolerance of linseed. There were 1552 overlapping up-regulated genes between Z141 and NY-17 under DS, and the GO items were mainly enriched in two distinct functional categories, including proline biosynthesis and reproductive development. The proline biosynthesis category “proline biosynthetic process (GO: 0006561)”, “proline metabolic process (GO: 0006560)”, “glutamine family amino acid biosynthetic process (GO: 0009084)” and “glutamine family amino acid metabolic process (GO: 0009064)”, whereas the abiotic stress response category includeed “reproductive system development (GO: 0061458)” and “reproductive structure development (GO: 0048608)” (Additional files 13 and 14, Fig. 4a). Under RD stress, 2957 DEGs were both up-regulated in Z141 and NY-17. The GO items of these genes were also mainly enriched in the proline biosynthesis category with “proline biosynthetic process (GO: 0006561)” and “proline metabolic process (GO: 0006560)”, and in the abiotic stress response category with “response to abscisic acid (GO: 0009737)”, and “response to desiccation (GO: 00009269)”, “response to acid chemical (GO: 0001101)” (Additional files 13 and 14, Fig. 4b). The GO terms of downregulated genes overlapping between Z141 and NY-17 under DS and RD conditions were mainly enriched in functional categories related to photosynthesis (Additional file 12).

There were 1693 specifically up-regulated DEGs under DS in Z141, and the GO items of these genes were mainly enriched in “abscission (GO: 0009838)”, “defense response (GO: 0006952)” and “NADP biosynthetic process (GO: 0006741)” (Additional files 13 and 14), whereas under RD, the GO terms were mainly enriched in “jasmonic acid biosynthetic process (GO: 0009695)” and “jasmonic acid metabolic process (GO: 0009694)” (Additional files 13 and 14). The uniquely upregulated genes showed more enrichment in pathways closely related to plant drought resistance, such as jasmonic acid biosynthesis, abscission and NADP biosynthesis, than in other pathways.. In contrast, the GO terms for genes upregulated in NY-17 under DS were mainly enriched in the RNA regulation functional category with “ncRNA metabolic process (GO: 0034660)”, “ncRNA processing (GO: 0034470)”, and “tRNA processing (GO: 0008033)” terms (Additional files 13 and 14). Under RD, the GO terms for genes in only NY-17 were mainly enriched in “phenylpropanoid biosynthetic process (GO: 0009699)” and “phenylpropanoid metabolic process (GO: 0009698)” (Additional files 13 and 14).

### Reduce and visualize GO (REVIGO) analysis

**Fig. 5:**
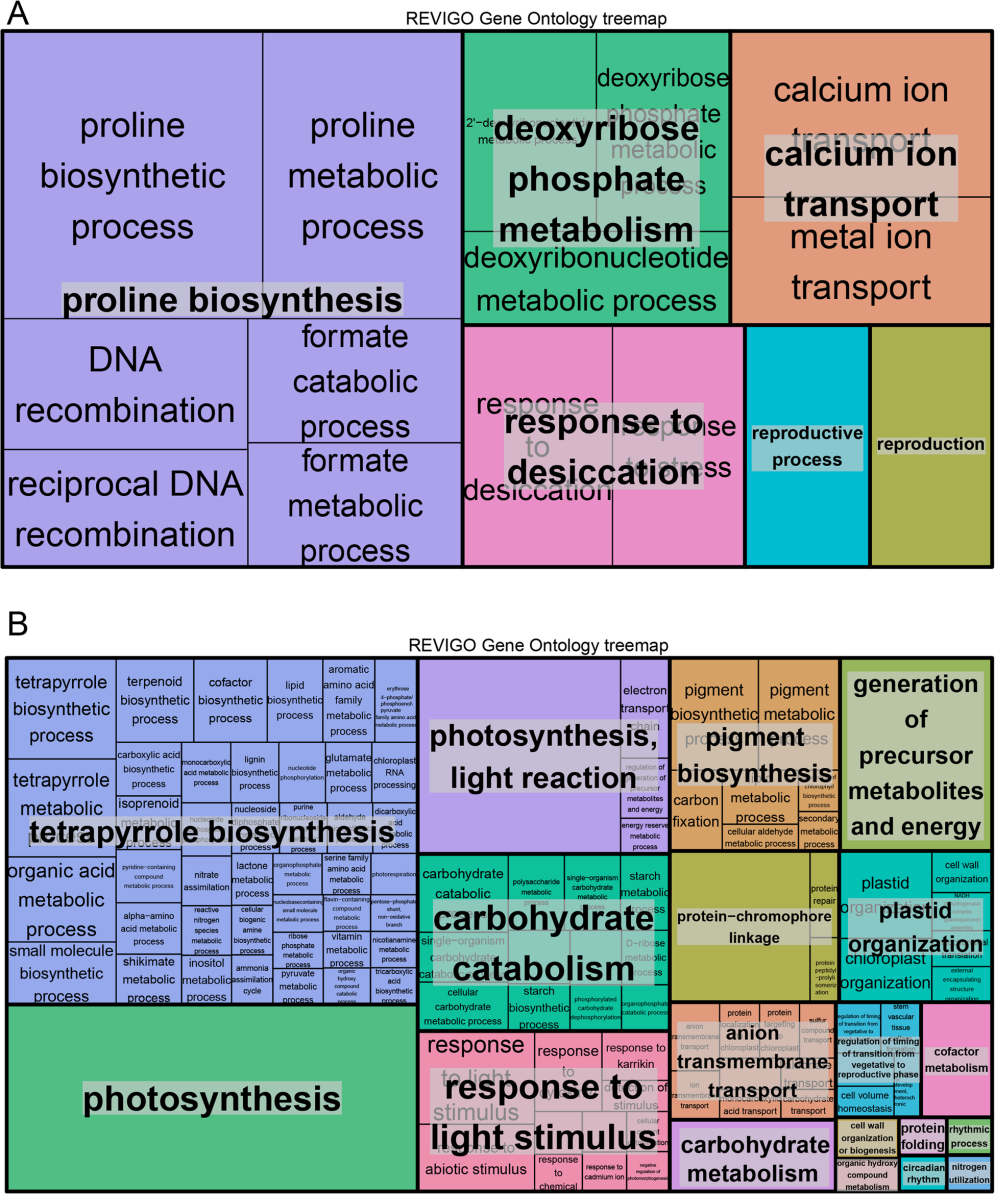
Gene Ontology (GO) based pathway analysis using REVIGO for up- and downregulated DEGs in Z141 under DS (**a** and **b**) represent the biological process (BP) up- and downregulated DEGs in Z141 under DS, respectively

To remove the insignificant GO terms which *p.* adjust value > 0.05 and visualize the GO difference between only Z141 and NY-17 genotypes, we submitted upregulated and downregulated enriched GO categories from Z141 and NY-17, respectively, with a false discovery rate (FDR) < 0.05, respectively, to REVIGO analysis (Fig. 5a, b). Graphical results revealed that highly significant biological process (BP) GO terms such as proline biosynthesis process (GO: 0006561), DNA recombination (GO: 0006310), reciprocal DNA recombination (GO: 0035825), response to desiccation (GO: 0009269) and response to stress (GO: 0006950) were upregulated in Z141 under DS. These GO terms are enriched in 6 main functional groups, namely, proline biosynthesis, response to desiccation, deoxyribose phosphate metabolism, calcium ion transport, reproductive process, and reproduction (Fig. 5a). Although DEGs of proline biosynthesis (GO: 0006561), response to abiotic stimulus (GO: 0009628), and mismatch repair (GO: 0006298) were significantly upregulated in NY-17 under DS stress, more DEGs were enriched in RNA modification (GO: 0009451), RNA processing (GO: 0006396), and ncRNA processing (GO: 0034660). Therefore, the upregulated DEGs in NY-17 under DS were mainly enriched in RNA modification, anatomical structure homeostasis, ribosome biogenesis, protein refolding, reproductive system development, and reproductive process (Additional file 15).

The REVIGO analysis showed that the functional groups of enriched GO terms were more similar between Z141 and NY-17 under RD stress than under DS. The GO terms were mainly enriched in proline biosynthesis, response to stress, metal ion transport, and inorganic ion homeostasis. These functional groups are closely related to the response of plants to DS; however, in NY-17, the DEGs of leaf senescence (GO: 0010150) and ageing (GO: 0007568) were upregulated, and this result is consistent with the phenotype of NY-17 under RD stress (Additional file 15).

The downregulated GO terms in both Z141 and NY-17 under DS and RD stress were mainly involved in tetrapyrrole biosynthesis, photosynthesis, and light reactions (Additional file 15, Fig. 5b). This result is consistent with GO analysis and indicated that the effects of DS on the linseed aboveground parts mainly involved photosynthesis.

### Functional analysis of DEGs using MapMan analysis

**Fig. 6:**
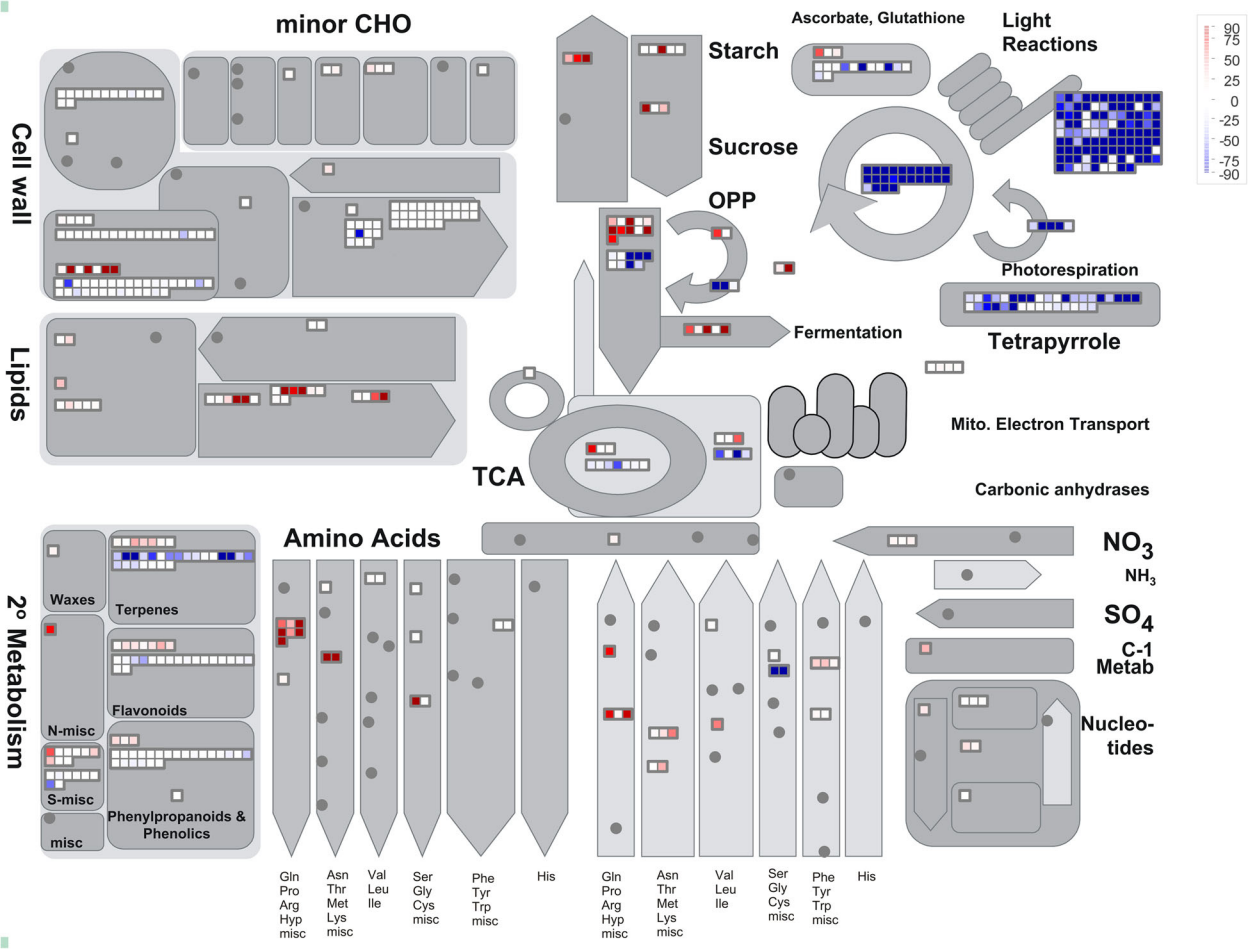
MapMan visualization of drought stress-responsive DEGs in Z141 and NY-17 under DS (**a**, **c**) and RD stress (**b**, **d**). Up- and downregulated DEGs are represented in red and blue, respectively. Colour brightness indicates the degree of difference, as shown by the scale on the right

MapMan is a user-driven tool that projects large data sets onto diagrams of metabolic pathways and other processes. Therefore, in this study, we used it to explore the effects and changes induced under DS in linseed leaf tissues. We input data of specific BP DEGs that were co-upregulated or co-downregulated in Z141 and NY-17 under DS or RD stress and used the reference Lusitatissimum_200. m02. Figure 6 and additional file 16 shows an overview of Z141 and NY-17 up- and downregulated DEGs involved in metabolic pathways under DS and RD stress.

The results showed that of the Z141 and NY-17 DEGs that were up- or downregulated DEGs under DS stress, 1483 upregulated DEGs and 2478 downregulated DEGs were mapped, and of them, only 178 and 581 are visible in Fig. 6 and additional file 16. In contrast, of the Z141 and NY-17 DEGs that were up- or downregulated under RD stress, 2973 upregulated DEGs and 3581 downregulated DEGs were mapped; 400 and 723 of these are visible in Fig. 6 and additional file 14. Consistent with the GO enrichment analysis, up- and downregulated DEGs were mainly enriched in similar functional groups and pathways by MapMan analysis.

It is evident from both GO enrichment and MapMan analysis that upregulated DEGs were mostly enriched in the glutamine family amino acid biosynthesis process (GO: 0009084) and proline biosynthetic process (GO: 0006561). The downregulated DEGs were mainly enriched in photosynthesis (GO: 0015979), light harvesting in photosystem I (GO: 0009768), and light harvesting (GO: 0009765). These terms are most likely to play an essential role in regulating DS in linseed.

### PPI network analysis

**Fig. 7:**
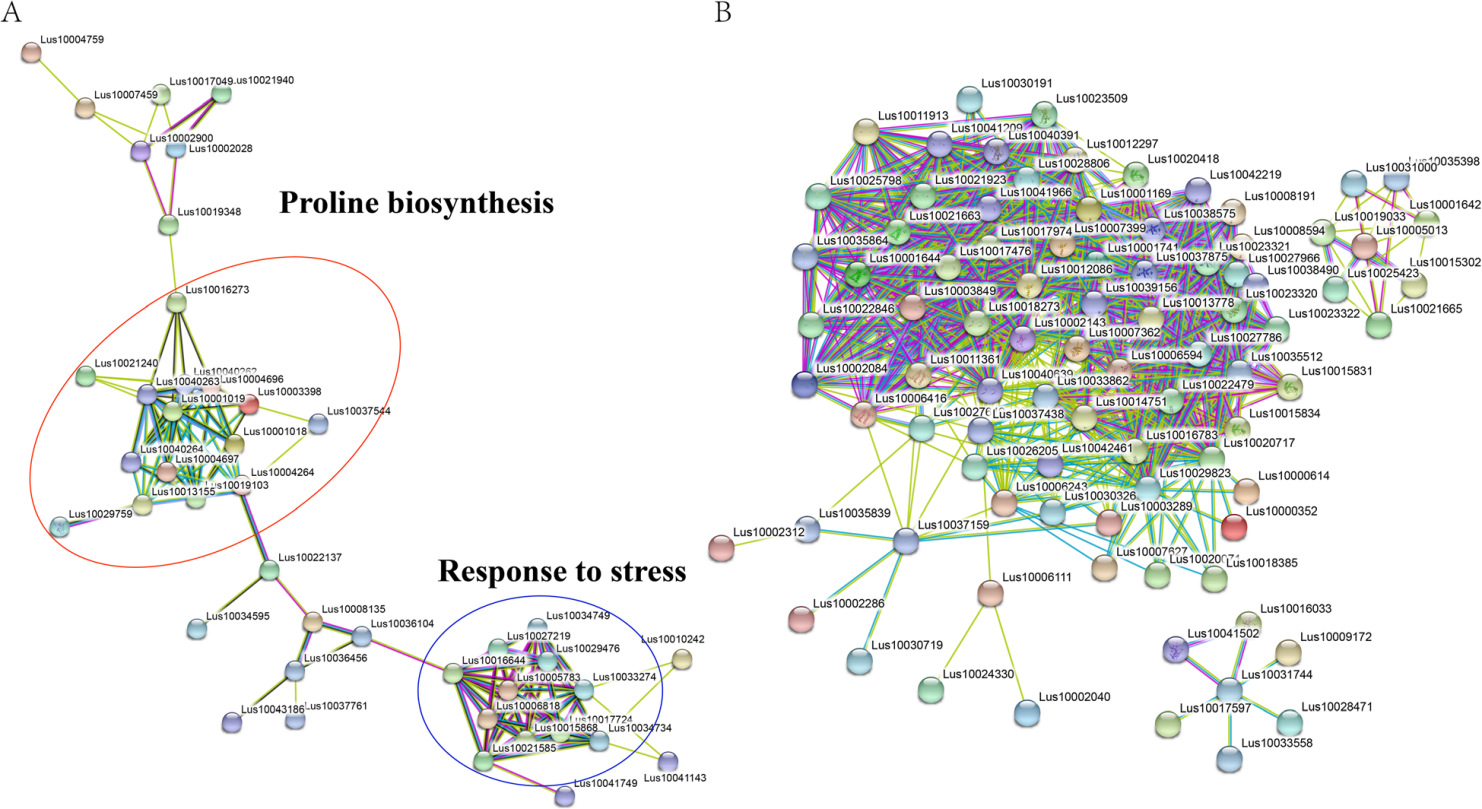
Interaction networks of stress-responsive genes identified by RNA-Seq analysis. **a** and **b** Protein-protein interactions among up- (**a**) and downregulated (**b**) DEGs in Z141 and NY-17 responsive to both DS and RD stress

To further explore the protein interactions during DS, we constructed a PPI network of all the up- and downregulated DEGs and identified them in linseed leaf tissues using the STRING program. For the upregulated DEGs, we identified two interaction subnetworks that were predicted from 43 nodes of proteins with a PPI enrichment *p*-value< 1.0e-16 at the medium confidence parameter level. In this network analysis, we identified RAD50 (DNA repair protein 50) interacting protein 1 (RIN-1) as a hub gene that interacted with proline biosynthesis and response to stress (Fig. 7a). For the downregulated DEGs, there were 94 nodes of proteins with PPI enrichment (Fig. 7b). Almost all of the nodes were concentrated on photosynthesis or related regulation networks. This result is completely consistent with the results of our previous analysis.

### Identification of transcription factors (TFs) temporarily up- and downregulated in response to DS and RD

TFs have play irreplaceable roles in the response to various abiotic stresses by modulating target gene expression [26]. To understand the essence of regulatory processes during DS and RD treatment, a domain searching method was used to first predict TFs in Z141 and NY-17 on a whole-genome scale based on our identified non-redundant linseed unigenes. A total of 4936 linseed TF genes distributed among 50 families were identified (Additional file 17) [27].

**Fig. 8:**
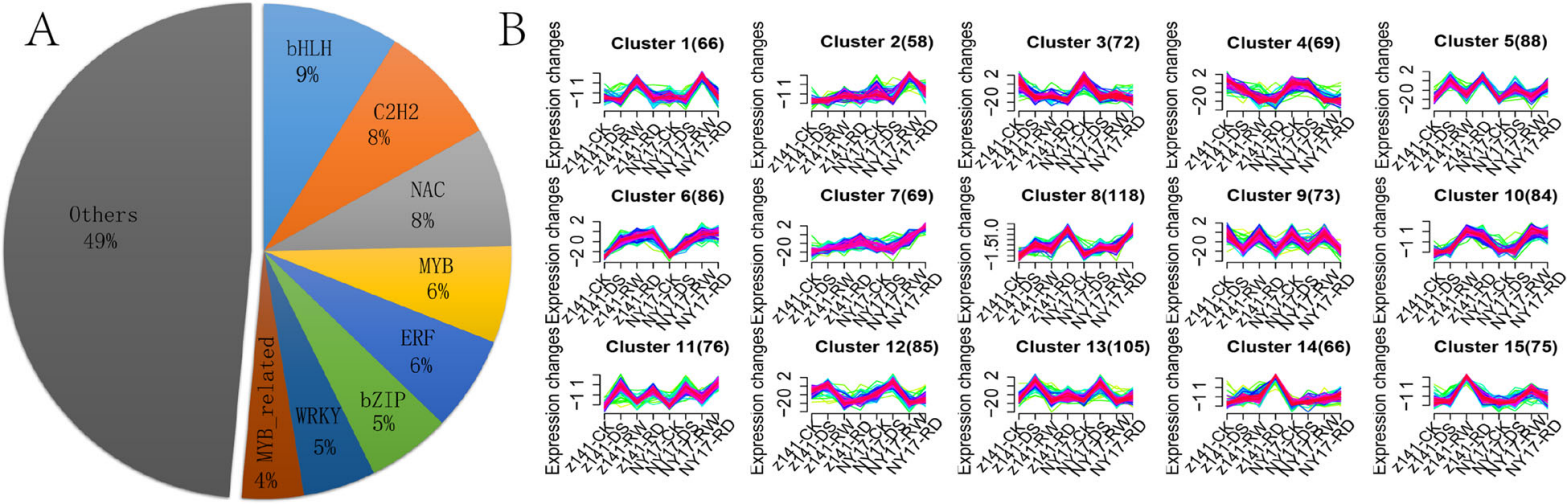
Clustering analysis of DS and RD responsive TFs. **a** Pie chart showing the top 8 TF families, which together contain approximately 50% of the differentially expressed TF genes. **b** Clustering of the differentially expressed TFs based on their expression patterns in Z141 and NY-17 under CK, DS, RW and RD. Fifteen clusters comprising 1190 TFs are exhibited here, and the numbers in parentheses indicate the number of TFs in the corresponding clusters. The X axis represents treatment conditions and the y axis represents centralized and normalized expression values. The red lines represent the mean expression trend of TFs (green lines) belonging to each cluster

To profile a stress-responsive TF open reading frame collection (TFome) under DS and RD, we focused on TF genes exhibiting diverse expression patterns with stress changes, including continuous upregulated, continuous downregulated an early peak in expression and a late peak in expression. As a result, 1190 TFs distributed in 50 families were found to be differentially regulated in response to at least one stress. (Fold change ≥2 and FDR adjusted p-value < 0.01). Eleven TF families accounted for approximately half of the stress-responsive TF genes, including bHLH (9%), C2H2 (8%), NAC (8%), MYB (6%), ERF (6%), bZIP (5%), WRKY (5%) and MYB-related (4%) (Fig. 8a).

Moreover, the 1190 TFs were further classified into 15 clusters according to their expression patterns by performing Mfuzz program analysis in R software. Clusters 5, 8,11 and 13 consisted of 387 TFs mainly upregulated by DS and RD, including *DREB*, *HSF* and *NF-YA10*, which have been confirmed to be key regulators of plant abiotic resistance pathways (Fig. 8b and Additional file 18).

### Candidate gene prediction

**Fig. 9:**
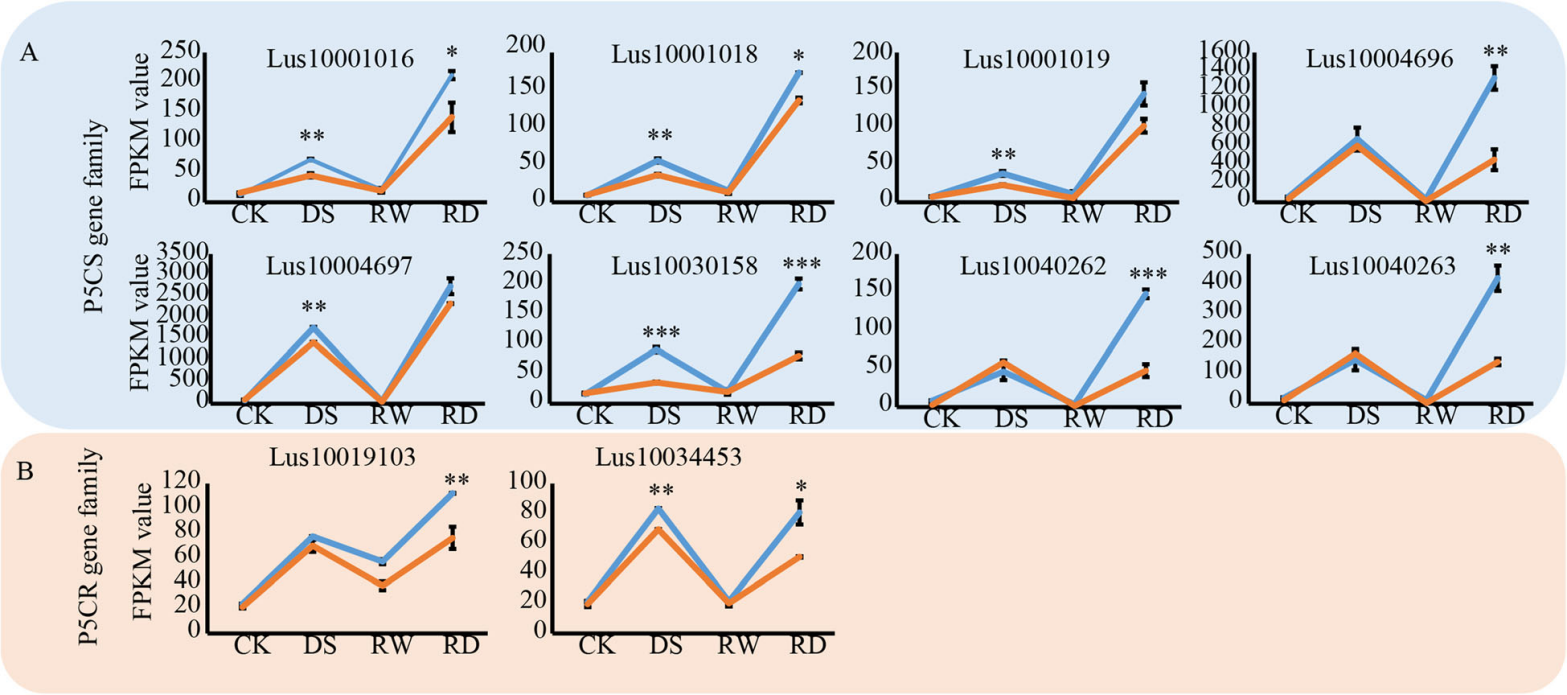
Gene expression levels of *P5CS* and *P5CR* gene family members. **a** Expression patterns of 8 *P5CS* gene family members. The blue and orange lines represent the gene expression of *P5CS* gene family members in Z141 and NY-17, respectively. **b** Expression patterns of 2 *P5CR* gene family members. The blue and orange lines represent the gene expression of *P5CR* gene family members in Z141 and NY-17, respectively. The error bar represents the standard error. * indicates *p* < 0.05; ** indicates *p* < 0.01

By considering the results of GO enrichment, MapMan, and PPI network analysis and gene annotations, we screened DS-responsive genes from the DEGs that have functions related to proline biosynthesis, response to stress, response to water, and cellular response to abiotic stimulus for candidate gene analysis. A total of 508 DEGs related to the above functions were screened for candidates for gene prediction analysis in Z141 and NY-17, respectively (Additional file 19, Table 2). *P5CS* gene family encodes 1- pyrrolin-5 - carboxylate synthase (*P5CS*), which is the key rate-limiting enzyme in plant proline biosynthesis [28]. Previous studies have shown that overexpression of members of the *P5CS* gene family can significantly increase the proline content in plant cells and improve the drought tolerance of plants [29]. Usually, the *P5CS* gene family of other plants has 2–4 members [28]. But in linseed, we have identified 8 members, and their expression patterns closely match with our repeated drought patterns (Additional file 19 and 20, Fig. 9a). In addition, the expression level of most *P5CS* gene family members in Z141 was significantly higher than that in NY-17 under drought stress. (Fig. 9a). P5CR gene family members encode the last enzyme in the plant proline biosynthesis pathway, overexpression *P5CR* gene will significantly improve the photosynthetic response of *Arabidopsis* under drought and high-temperature stress [30, 31]. In this study, we found that the expression level of *P5CR* gene, such as *Lus10034453*, in Z141 was significantly higher than that in NY-17 under drought stress, and its expression pattern also matched our drought treatment model (Fig. 9b). Higher gene expression of *P5CR* family members may be conducive to proline accumulation. These results indicate that the members of the *P5CS* and *P5CR* gene families are closely related to drought tolerance in plants. Moreover, in this study, we found that some encoding dehydrin genes were also rapidly increased their expression levels under drought stress (Additional file 19 and 20). Previous studies have shown that overexpression dehydrin genes will enhance the drought tolerance of plants [32, 33]. Therefore, we hypothesize that proline, dehydrin, and DNA repair might play important roles in regulating drought tolerance in linseed. Hence, based on all the above analyses, 24 genes (including 8 P5CS gene family members, 2 P5CR gene family members, 8 DNA repair genes and 6 dehydrin -encoding genes) were considered the most likely candidate genes enabling drought tolerance (Additional file 19 and 20). However, further validation and verification are needed to check their actual roles in drought tolerance.

**Table 2:** The number of DEGs related to proline biosynthesis, response to stress, cell response to stress, photosynthesis, and carbohydrate catabolism, in Z141 and NY-17

Trait Name	Description	Up-DEGs				Down-DEGs			
		Z141		NY-17		Z141		NY-17	
		DS	RD	DS	RD	DS	RD	DS	RD
**proline biosynthesis**	**proline metabolic process**	12	15	11	14	0	0	0	0
	**proline biosynthetic process**	12	13	11	12	0	0	0	0
**response to stress**	**response to acid chemical**	44	77	34	65	0	0	0	0
	**response to stress**	180	276	123	222	0	0	0	0
	**response to water**	17	25	12	18	0	0	0	0
	**response to inorganic substance**	25	43	21	33	0	0	0	0
	**response to X-ray**	3	3	3	3	0	0	0	0
	**response to lipid**	32	54	23	43	0	0	0	0
	**response to chemical**	106	168	77	137	0	0	0	0
	**response to oxygen-containing compound**	49	87	38	75	0	0	0	0
**cell response to stress**	**cellular response to abiotic stimulus**	11	14	10	15	0	0	0	0
	**cellular response to acid chemical**	20	29	17	29	0	0	0	0
	**cellular response to radiation**	8	10	8	12	0	0	0	0
	**cellular response to ionizing radiation**	3	3	3	3	0	0	0	0
	**cellular response to gamma radiation**	3	3	3	3	0	0	0	0
	**cellular response to X-ray**	3	3	3	3	0	0	0	0
**photosynthesis**	**photosynthesis, light harvesting**	0	0	0	0	47	49	41	45
	**photosynthesis, light harvesting in photosystem I**	0	0	0	0	31	33	30	32
	**regulation of photosynthesis**	0	0	0	0	11	10	8	10
	**photosynthesis**	0	0	0	0	172	180	137	171
	**photosynthesis, light reaction**	0	0	0	0	87	95	67	87
	**photosynthesis, dark reaction**	0	0	0	0	6	7	5	6
	**regulation of photosynthesis, light reaction**	0	0	0	0	9	10	7	9
**carbohydrate catabolism**	**carbohydrate metabolic process**	0	0	0	0	304	300	241	285
	**carbohydrate catabolic process**	0	0	0	0	89	84	63	79
	**single-organism carbohydrate catabolic process**	0	0	0	0	36	39	23	38

### Validation of isoforms by RT-PCR

**Fig. 10:**
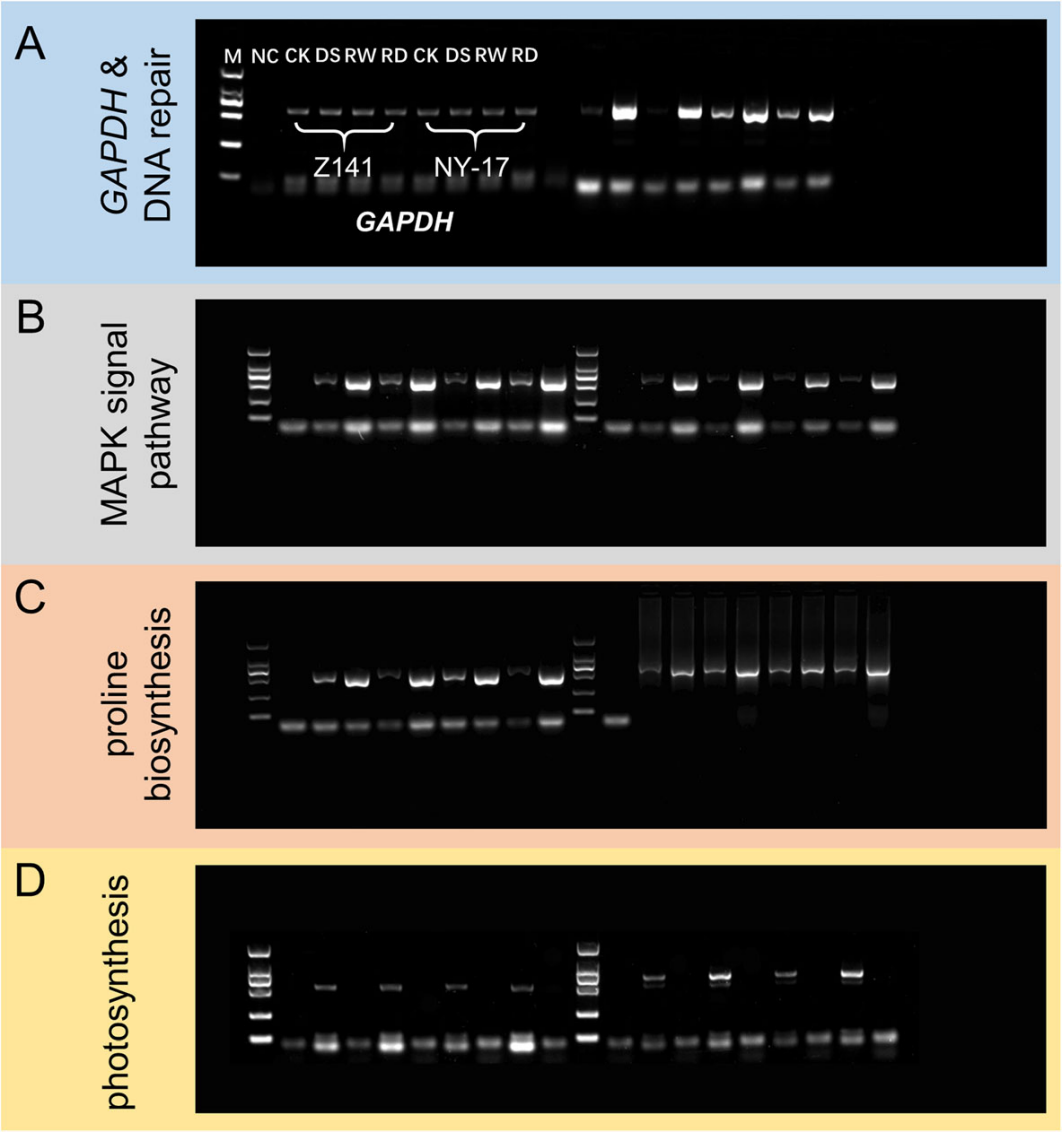
Validation of differentially expressed genes by RT-PCR. **a**-**d** Gene expression changes of *GAPDH*, DNA repair, MAPK signalling pathway, proline biosynthesis and photosynthesis genes under CK, DS, RW and RD in Z141 and NY-17. M indicates the DL2000 DNA marker; NC indicates negative control and CK, DS, RW, and RD indicate control check, drought stress, re-watering and repeat drought, respectively. Note: This image has been optimized by reasonable cropping, the original and full-length gel images please see additional file 24

Expression analysis of differentially expressed functional candidate genes, associated with DNA repair, the MAPK signalling pathway, proline biosynthesis, and photosynthesis that were selected from transcriptome data, was validated by RT-PCR. The results (Fig. 10a-d) demonstrated that transcript abundances of selected genes were consistent with the transcriptome analysis results, thereby validating the reliability of our annotated transcriptome data for future studies.

## Discussion

Linseed is an important special oil crop and has excellent drought tolerance; in extreme cases, it can complete its life cycle in areas where annual rainfall is only 200 mm [34]. Plant DS tolerance is controlled by quantitative traits; therefore, understanding linseed adaptive mechanisms and genes related to drought tolerance can provide new ideas for drought tolerance research in other crops. Although linseed whole-genome sequencing data were published in 2012, it was not until 2017 that the first report of a transcriptome dataset of flax at different developmental stages under DS was published by Dash [35]. Unfortunately, the author did not analyse the data; therefore, the molecular mechanism of its drought tolerance remains a black box. In addition, very limited attempts have been made to understand drought tolerance at the second- generation transcriptome level. In this regard, the present study explored drought tolerance in two linseed genotypes (Z141 and NY-17) at the seedling stage using single-molecule long-read sequencing.

In this study, we regulated the absolute soil water content (ASWC) by measuring the weight of the soil. This method enabled us to investigate the phenotypic and gene expression changes of plants under at various DS levels, and these stresses are reproducible because the ASWC is not dependent on the type of soil. After that, we measured the degrees of LWC, plant height, and biomass dry weight of Z141 and NY-17 at different drought levels. RLWC is an important evidence for plant drought tolerance, it reflects the plant tissues water status and the he ability of plants to retain water. ALWC is closely related to the characteristics of plant itself. Therefore, in this study RLWC had a significant difference between Z141 and NY-17, while ALWC did not have significant difference. We observed that the biomass reduction rate in NY-17 was significantly higher than that in Z141, but the RLWC in Z141 was significantly higher than that in NY-17 for the control when the ASWC was lower than 10%. A large number of studies have shown that under DS, high LWC values significantly reduce yield loss [36, 37]. Biomass accumulation is also significantly affected by DS, and the biomass reduction rates of plants are inversely proportional to their drought tolerances [38, 39]. Interestingly, in recent years, some studies have shown that drought-related SNPs are usually associated with plant height [40, 41]. In the study of woody plants, plants should be short under drought conditions [42]. These findings are similar to our conclusions, regardless of whether there is DS, and the NY-17 plants were significantly taller than the Z141 plants (Table 1 and Additional file 2). Thus, we conclude that compared with NY-17, Z141 has better drought tolerance. This finding is different from our initial hypothesis because NY-17 is widely planted in arid areas such as the Gansu Province in China, where the average annual precipitation is lower than 400 mm.

Under DS the differences between Z141 and NY-17 occurred in not only phenotype, but also gene expression patterns. In our study, we observed that more DEGs were identified in Z141 than in NY-17 under DS. For example, 3245 DEGs were upregulated and 4167 DEGs were downregulated in Z141 under DS, In contrast, only 2381 genes were upregulated and 3515 DEGs were downregulated in NY-17, under DS. This result suggests that Z141 responds more rapidly or sensitively to DS than NY-17 does. In addition, we also found that the number of DEGs under RD stress was significantly greater than that under DS stress (Fig. 9). This finding is similar to results from previous studies, which suggests that a greater number of variable physiological responses occurred in repeated than in sustained drought treatments [43]. Further analysis found that the numbers of specifically up- and downregulated DEGs in Z141 were significantly greater than those in NY-17. In this study, for example, under DS, more than 52% of the total upregulated DEGs were specifically upregulated in Z141, and only 34% were specifically upregulated in NY-17 (Fig. 3c). Therefore, we hypothesized that the significant differences in gene expression patterns may be the reason for Z141 having better drought tolerance.

From both GO enrichment and MapMan analyses, we found that proline biosynthesis genes were significantly upregulated when Z141 and NY-17 were under DS. These findings are similar, to those reported by earlier studies, which revealed a strong correlation between proline accumulation and drought tolerance. Dramatic accumulation of proline is a common physiological response in plants exposed to various abiotic stresses. Previous studies have shown that proline seems to have more roles under stress conditions, such as stabilization of proteins, membranes, and subcellular structures, and protecting cellular functions by scavenging reactive oxygen species (ROS), than in the absence of stress [44–46]. P5CS is the key and rate-limiting enzyme that catalyses the activation of glutamate by phosphorylation and the reduction of the labile intermediate γ-glutamyl phosphate into glutamate semialdehyde (GSA) in the higher plant proline biosynthesis pathway. Overexpression of *P5CS* significantly increases plant proline production and accumulation and increases drought and salt tolerance in transgenic crops [25, 29, 47]. In the present study, we observed that the gene expression levels of *P5CS* and *P5CR* in Z141 were significantly higher than those in NY-17 (Fig. 9a). Another interesting finding in this study was the number of members in the P5CS family in linseed, which was significantly higher than that in other crops. Normally, there are approximately two or three family members in the *P5CS* gene family, namely, *P5CS1*, *P5CS2* and *P5CS3* [28], but in our study, we identified 8 family members in the *P5CS* gene family (Fig. 10a). More *P5CS* gene family members, higher gene expression and faster proline accumulation may be important factors for linseed survival in arid environments.

In addition to differences in gene expression patterns, there are also significant differences in the number of DEGs, especially specific up- and downregulated DEGs, in Z141 and NY-17 under DS. In this study, we observed that under DS, the numbers and percentages of genes specifically up- or downregulated in Z141 were greater than those in NY-17. For example, under DS, a total of 3245 genes were upregulated in Z141, of which 1693 genes (accounting for 52.2%) were specifically upregulated (Fig. 2c). In contrast, 2381 genes were upregulated in NY-17, of which only 829 genes, accounting for 34.8%, were specifically upregulated (Fig. 2c). These specifically functionally regulated genes were also different. For example, under DS, the specifically upregulated genes in Z141 were mainly associated with NADP biosynthesis, abscission, defense response and the MAPK signaling pathway (Additional file 12), while the specifically upregulated genes in NY-17 were mainly involved in RNA regulation (Additional file 12). Previous studies have shown that the expression of NADP biosynthesis genes is upregulated when plants are under DS [48, 49]. Despite some studies suggesting that NADP genes could be related to ABA-mediated signaling, the mechanism remains unclear, and more studies are warranted [50]. Our study revealed an upregulation of NADP biosynthesis genes in Z141 leaves under DS, which suggests that NADP may compensate for a deficiency in CO_2_ in the light-independent reactions caused by DS. Thus, the specifically up- or downregulated genes in Z141may explain why this linseed variety has better drought tolerance than NY-17.

Most plant drought tolerance studies have been conducted by considering stress as a single event that occurs once in the life of a plant; however little is known about when recurrent drought episodes occur. A study in two shortgrass species found that drought timing and lack of previous drought exposure determined their sensitivity to water stress [51]. In contrast, some studies have found that plants exposed to multiple drought cycles can develop a differential acclimation that potentiates their defense mechanisms, allowing them to be kept in an ‘alert state’ to successfully cope with further drought events [52, 53]. In our study, we found similar results. For example, the gene expression levels of *P5CS* and *P5CR*, which are the key enzymes of proline biosynthesis in plants under RD stress, were significantly higher under RD stress than those under DS. Whether plants have “memory” has been the focus of research in recent years [54–56]; however, in our study, we observed that the functional categories of unique downregulated genes were significantly different between Z141 and NY-17 under DS but very similar under RD (Additional file 11). The difference in linseed responses to RD stress suggests that linseed might develop DS “memory”, thus changing its gene expression pattern to adapt quickly to future drought events [57].

Approximately 7% of the coding sequences were associated with TFs, which play a central role in regulating gene responses to abiotic stresses in plants [58–60]. In this study, we predicted 4936 potential TFs in the linseed genome, accounting for approximately 9% of the total genes, and representing nearly twice the number of TFs registered in plantTFDB (2481, Additional file 14). Numerous studies have shown that *DREB* is a master regulator of gene networks in the plant acclimation response to drought by regulating responsive gene expression by binding to cis-acting elements [61]. Similarly, one-third of *DREB* family members were significantly upregulated under DS. Many other TFs, such as *HSF* and *NF-YA10*, were also specifically upregulated under DS. Previous studies have shown that HSF and NF-YA10 can increase plant high temperature and salt tolerance respectively [62, 63]. However, in this study we ensured that the temperature (~ 22 °C) was suitable for linseed growth during drought treatment. This may suggest that the molecular mechanisms of abiotic stress tolerance in plants to factors such as drought, high-temperature, and saline-alkali conditions are not independent and that there may be some interactions [64]. Moreover, some TFs related to plant drought avoidance were also specifically upregulated under DS in this study. NF-YC3 and WRKY75 have been proven to induce flowering or regulated root development in plants under abiotic stress [65–67]. Unexpectedly, some validated negative stress regulators were also unregulated under DS which complicates understanding the molecular mechanisms underlying linseed tolerance to abiotic stress. For example, MYB102 has been proven to delay leaf senescence and decrease abiotic stress tolerance in *Arabidopsis thaliana* [68]. This information indicates that even under abiotic stress the up-regulated TF may not necessarily help improve plant abiotic tolerance. In conclusion, our results indicate that TF regulation of linseed drought tolerance is considerably complex but it is still helpful for us to understand the molecular mechanisms underlying linseed tolerance to DS.

DNA is a very sensitive target of hydroxyl radicals [69]. ROS generation is one potential cause of DNA damage under drought [70, 71]. Oxidative damage of DNA involves base modifications and strand cleavage, which lead to senescence and diseases in biological systems. Timely and accurate repair of DNA damage is the key point of plant survival under DS. For example, overexpression of *OsNAC14*, which has a DNA repair function in rice, has been demonstrated to significantly induce tolerance to drought [72]. In our study, 8 DNA repair-related DEGs were found to be significantly upregulated in Z141 and NY-17 (Additional file 16). Furthermore, PPI network analysis reported an interaction between proline biosynthesis and stress response-related genes (Fig. 7). Although previous studies showed that proline can remove ROS and maintain cell function, the underlying mechanism has not been clear [73, 74]. This result indicated that proline not only maintains the normal osmotic pressure of cells but also protects DNA from ROS damage when plants are under DS.

**Fig. 11:**
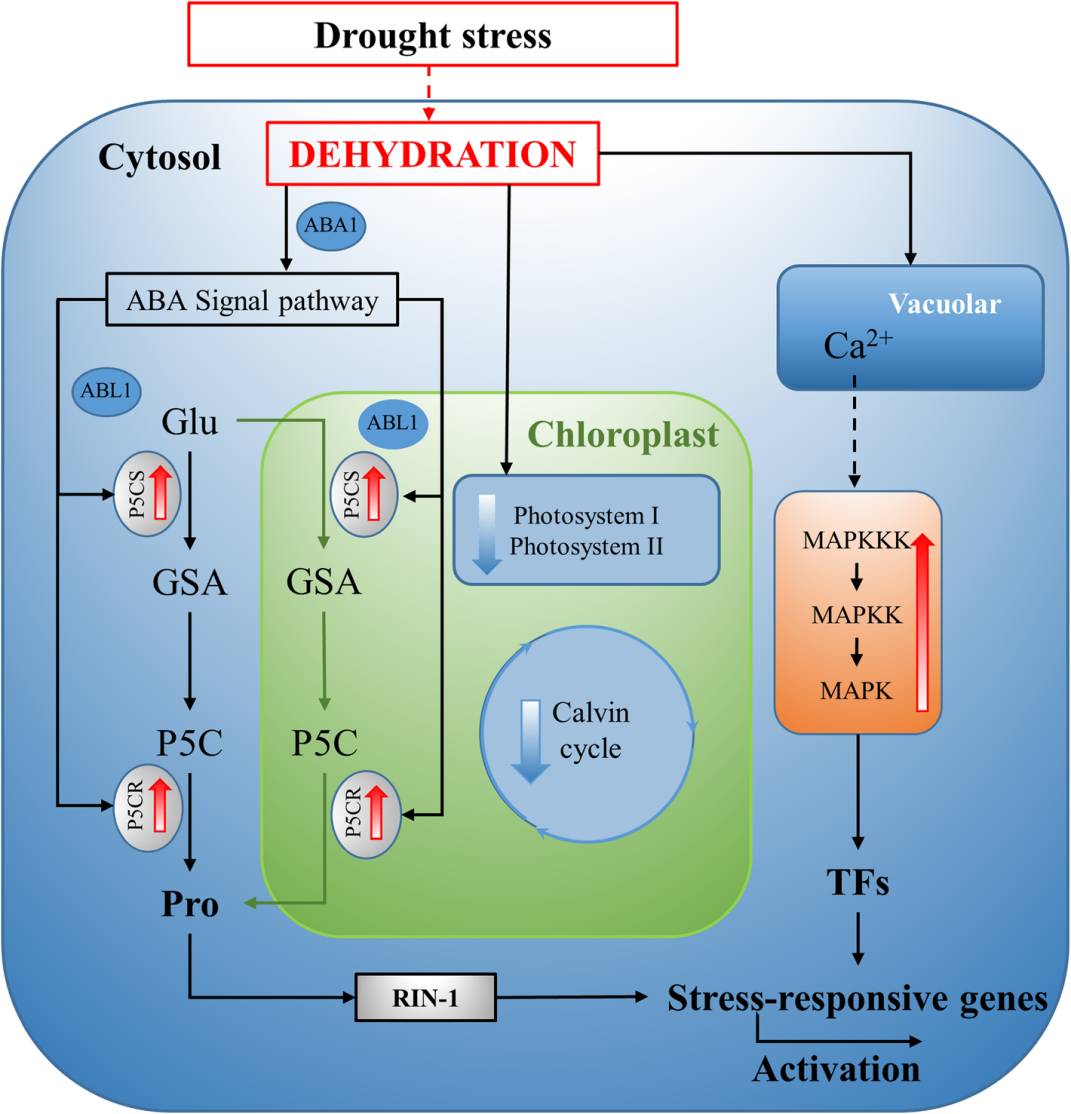
A schematic hypothetical model based on the findings of the present study and describing the regulatory network of linseed leaf responses. Red and green arrows illustrate up- and downregulated DEGs in Z141 and NY-17, respectively

Based on the above results, we proposed the model presented in Fig. 11. This model shows how proline biosynthesis, DNA repair, TF activities, and signaling terms, might regulated increased drought tolerance in linseed.

## Conclusions

Our results revealed that a group of genes involved in plant drought tolerance were upregulated in only the linseed variety with better drought tolerance under DS. In addition, more genes are involved in the linseed response to drought stress under RD. than under a single drought. Third, in this study, we found that the rate of proline accumulation affects linseed drought tolerance.

Finally, some of the TFs involved in the response to high temperature stress were expressed in linseed under DS, indicating that the linseed response to drought and high-temperature stress was cooperative rather than independent. Taken together, the results from this study deepen our understanding of the molecular mechanism of linseed drought tolerance and the orchestrated linseed responses to RD stress, which frequently occur under field condition, and provide a new perspective to understand the drought adaptability of linseed. To our knowledge, this is the first study to compare and analyse the gene expression patterns of linseed varieties with different drought tolerances under different drought treatments on a genome-wide scale using single-molecule long-read sequencing. Therefore, our study will contribute to the current body of knowledge on drought tolerance gene identification and functional analysis in linseed.

## Methods

**Phenotyping for drought tolerance in the linseed seedling stage.**

Linseed variety NY-17 (accession no.: NYS-2005001) was provided by the Guyuan Branch of the Ningxia Academy of Agriculture and Forestry Sciences, while Z-141 (China metaphase germplasm bank no.: HM00001753) which was introduced from Alberta, Canada, was provided by the Zhangjiakou Academy of Agricultural Sciences. Z141 and NY-17 seeds sterilization method as described by Seta-Koselska [75]. Then transferred into 7 cm diameter plastic pots filled with a mixture of cultivation soil and vermiculite in a 1:1 ratio. After germination, the pots were transferred to culture room with a 16 h photoperiod and a temperature of 22 °C.

**Fig. 12:**
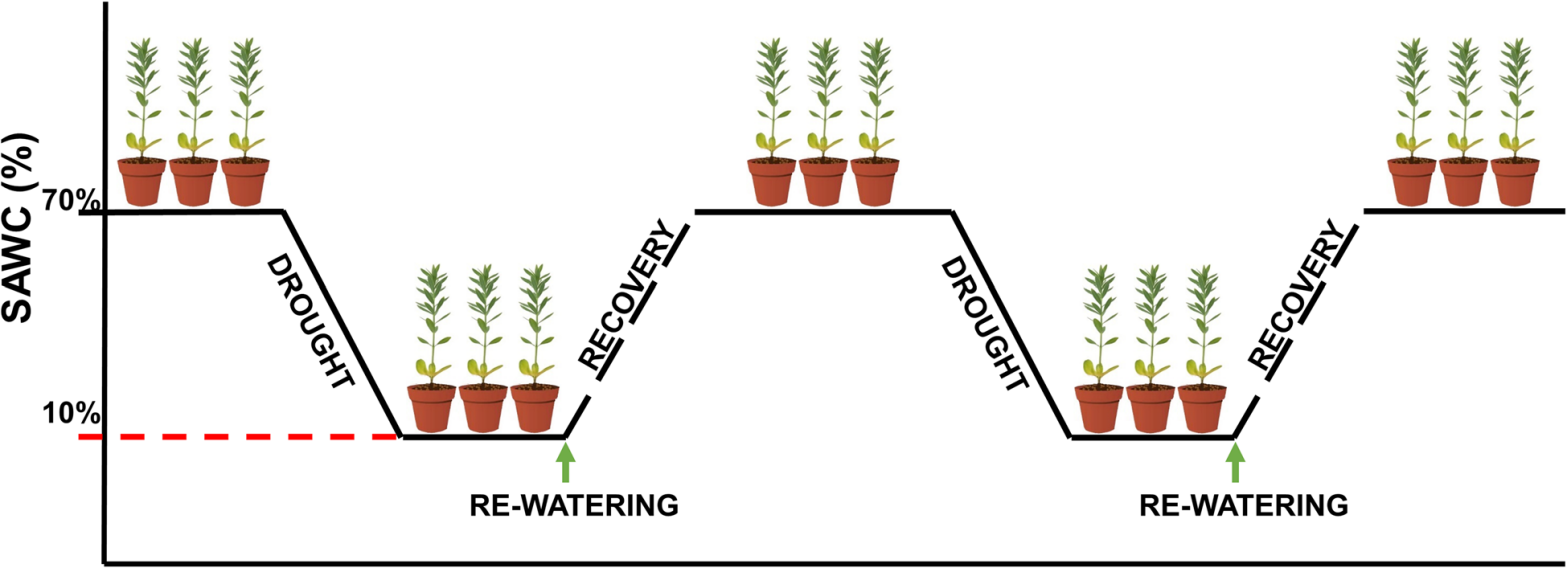
Schematic representation of the cycles of dehydration and re-watering. Each drought cycle consisted of a dehydration phase followed by a recovery period. Dehydration was imposed by suspending irrigation until the ASWC reached approximately 10% (red dashed line) and the plants were kept at this soil water level for 2 days, after which the pots were re-watered until the ASWC was 70%. The plants were then maintained for 2 days

The drought stress expression has adopted completely random design (CRD) with control trial. The pot (7 × 7 × 7 cm) was filled with uniformly mixed loam (nutritive soil: vermiculite = 1:1), and keep the absolute soil water content (ASWC) at 70%. ASWC was measured as described by Turner [76]. The seeds of each linseed variety were randomly planted in 6 pots, 3 of which were randomly selected as control groups and the other 3 pots as experimental group. Each pot planted 6 linseed seeds and as a biological repeat, there were 3 biological repeats for drought stress and control respectively. DS and RD stress experiments began from 20d old after linseed germination, and refer to Menezes-Silva et al. [53]. Gradual reduced the soil water content of the experimental group until ASWC of ~ 10%. Two days after the ASWC reached 10%, measured the phenotypic traits of the experimental group and the control group, then the stressed plants were watered to reach 70% ASWC to help recover. The DS and RW treatments were repeated, and afterward, the irrigation was maintained normally until the maturation stage (Fig. 12). Leaf tissues were collected during each drought and RW treatment from six independent plants with three biological replicates.

### Measurement of phenotypic traits

The three drought tolerance related phenotypic traits, plant height, biomass, leaf water content (including ALWC and RLWC) ALWC and RLWC were measured as described by Ghashghaie [77] and Yamasaki [78] respectively. Three biological replicates were measured for all phenotypic traits. Specific measurement methods and formulas please see additional file 1.

### RNA isolation, library preparation, and transcriptome sequencing

Total RNA from leaf tissues was extracted using TRIzol reagent (Invitrogen), according to the manufacturer’s instructions. RNA concentration was measured using a NanoDrop 2000 spectrophotometer (ND-2000, Thermo Fisher Scientific, Inc., USA). RNA from 16 samples was pooled together at equimolar rations. Approximately, 2 μg of total RNA was used for cDNA synthesis using an optimized SMARTer PCR cDNA Synthesis Kit that had been optimized for preparing high-quality, FL cDNAs (TaKaRa Biotechnology, Dalian, China), which was followed by size fractionation (1–3 and > 3 kb) using the BluePippin™ Size Selection System (Sage Science, Beverly, MA). Iso-Seq libraries were subsequently constructed using the protocol by (https://www.pacb.com/wp-content/uploads/Procedure-Checklist-Iso-Seq-Template-Preparation-for-Sequel-Systems.pdf). Each SMRT cell line was sequenced using P6 C4 reagent on the PacBio RS II platform with 4 h sequencing movies.

### Illumina RNA-Seq library construction

mRNA was purified from the total RNA using poly T oligo-attached magnetic beads. Sequencing libraries were generated using the NEBNext® Ultra™ RNA Library Prep Kit for Illumina® (NEB, USA) following the manufacturer’s recommendations. The library quality was assessed on the Agilent Bioanalyzer 2100 system.

### Subread processing and error correction

PacBio raw data were preprocessed using the SMRT Pipe analysis workflow of the PacBio SMRT Analysis software suite (http://www.Pacb.com/products-andservices/analytical-software/smrt-analysis/). CCS reads were obtained from the P_CCS model. Briefly, raw polymerase reads were filtered and trimmed to generate the subreads and read of inserts (ROIs) using the RS_Subreads protocol, requiring a minimum polymerase read length of 50 bp, a minimum polymerase read quality of 0.75, a minimum subread length of 50 bp and a minimum of one full pass. FLNC reads were regarded as those containing a 5′ adaptor, 3′ adaptor and poly (A) tail in the expected arrangement with no additional copies of the adaptor sequence within the ROI.

Error correction of FLNC reads with the high-quality Illumina short reads was performed using Proovread version 2.12 with the default parameters [79]. The quality of Illumina short reads was examined using FastQC (v0.11.5; http://www.Bioinformatics.babraham.ac.uk/projects/fastqc). Sequencing adaptors and low-quality bases in short reads were trimmed before the error correction of FLNC reads. FLNC reads before and after error correction were respectively mapped to the IWGSC RefSeq v1.0 using GMAP (version 2016-09-14; https://github.com/juliangehring/GMAPGSNAP) [80].

### Identification of gene loci and isoforms

Based on read-genome alignments, FLNC reads with the same splicing junctions were collapsed into one isoform. The isoforms that had shorter 5′ terminal regions but shared the introns and splicing sites in the remaining region, were regarded as transcripts degraded at the 5′ terminal region and were filtered out. For the remaining isoforms, supporting evidence was examined. We retained isoforms supported with at least two FLNC reads, one FLNC read with a percent of identity (PID) higher than 99%, or all junction sites that were fully supported by Illumina reads or annotations of the IWGSC RefSeq v1.0. Isoforms that overlapped by at least 20% of their length on the same strand were considered to be from the same gene locus. Newly discovered loci and isoforms were compared with the reference genome annotation using the same criteria as for loci and isoform identification. Alternative splicing (AS) events were classified and characterized by comparing different isoforms of the same gene loci using as profile [81].

### Expression levels of genes and isoforms

For each sample, the trimmed short reads were mapped to the linseed reference genome (https://phytozome.jgi.doe.gov/pz/portal.html#!info?alias=Org_Lusitatissimum) using TopHat (v2.1.1; https://ccb.jhu.edu/software/tophat) [82]. RSEM (v1.3.0; https://deweylab.Github.io/RSEM) was used to calculate the isoform-level expression in terms of FPKM and TPM (transcripts per million) (Additional file 21) [83].

### Identification of differentially expressed genes and differentially spliced genes

To carry out differential expression analysis, transcript quantification results generated by RSEM were processed and refined in successive steps. First, transcript and gene read counts were generated from TPM data correcting for possible gene length variations across samples that were mainly derived from differential transcript usage using the tximport 1.10.0 R package with the option “lengthScaledTPM” [84]. Second, the corrected read count data of genes were used to estimate their expression in terms of FPKM. Third, the corrected read count data of genes were imported into the R package EdgeR to identify DEGs with the criteria of a fold change ≥2.0, an FDR-adjusted *p*-value < 0.05 and an expression level of FPKM≥1 in at least one sample for each comparison (Additional files 21 and 22) [85].

### Gene set enrichment and transcription factor (TF) analysis

The GO descriptions were obtained by BLAST and BLAST2GO searches and GO enrichment analysis using the R package clusterprofiler [86, 87]. TFs prediction was based on Zheng’s method using software iTAK software to predict the TFs by their protein sequence [88].

### PCA and heatmap analysis

PCA was performed using all samples’ FPKM values. The first principal component and second principal component values of each sample were calculated and plotted using the R package ggplot2 [89].

We selected the DEGs from all comparison groups, and then used the expression levels of these DEGs in all samples to perform hierarchical clustering. Finally, a heatmap was plotted using the R package pheatmap [90].

### REVIGO analysis

The results of GO enrichment with q value < 0.05 were imported it into the REVIGO database (http://revigo.irb.hr/) [91]. The final results were displayed in a tree diagram.

### MapMan analysis

The latest linseed mapping file provided by the MapMan database was downloaded. Then, the mapping file and the DEGs that were up- or downregulated in Z141 or NY-17 under DS or RD stress were imported into MapMan software *ver* 3.6.0 [92].

### PPI network analysis

The Search Tool for Retrieval of Interacting Genes/Proteins (STRING) online database was applied to construct up- or downregulated protein-protein interaction (PPI) networks using *Linum usitatissimum* as the background [93].

### Statistical analysis

For the phenotypic trait measurements, data from the different DS treatments were analysed separately. The significant effects of different varieties (fixed effects) and different DS treatments (random effects) were tested using ANOVA. For all comparisons involving pairs of means (Z141 versus NY-17), we used an independent *t*-test. Statistical analyses were performed using the software package SPSS ver. 21.0 for Windows (IBM Inc., New York, USA).

### Validation by RT-PCR

To further evaluate the reliability of our transcriptome data, total RNA of all the treated samples was extracted using the TRIeasy™ Total RNA Extraction Reagent (YEASEN, Shanghai, China) and first-strand cDNA synthesis was performed using the Hifair® 1st Strand cDNA Synthesis Kit (gDNA digester plus) (YEASEN, Shanghai, China) according to the manufacturer’s protocol. Subsequently, the expression of glyceraldehyde-3-phosphate dehydrogenase (GAPDH, Lus10014603) and seven candidate genes, including one DNA repair related gene (Lus10021585), two MAPK signaling pathway associated genes (Lus10012962 and Lus10001832), two proline biosynthesis-dependent genes (Lus10004697 and Lus10001016), and two photosynthesis-related genes (Lus10038490 and Lus10027966), were detected by RT-PCR using the first-strand cDNA of eight treatment samples. The coding sequences of all selected genes were used to design specific amplification primers (Additional file 20) in Primer Premier 6.0 software. All primers were synthesized by Sangon (Shanghai, China). Each 20 μL RT-PCR verification reaction contained 1.0 μL cDNA template, 1.0 μL each of the forward and reverse primers (10 μM), 10 μL 2 × Hifair Canace® Gold PCR Master Mix (containing 1.0 U/50 μL polymerase, 1.5 mM Mg^2+^, and 200 μM dNTP) (YEASEN, Shanghai, China), and 7 μL ddH2O. Double distilled water was used as a blank control template. The amplification conditions were as follows: an initial denaturation at 98 °C for 5 min; 34 cycles of 98 °C for 10 s, 60 °C for 20 s, and 72 °C for 30 s; and a final extension at 72 °C for 5 min. Finally, the PCR products were checked by 2.0% agarose gel electrophoresis.

## Supplementary Information

**Additional file 1: Table S1**. Methodology for measuring the drought-tolerant related traits.

**Additional file 2: Table S2**. Effect of drought stress (SAWS=10%) on drought tolerant related traits in Z141 and NY-17

**Additional file 3: Table S3**. Effect of drought stress on ALWC and RLWC in Z141 and NY-17.

**Additional file 4: Table S4**. Sequence summary of PacBio SMRT Cells.

**Additional file 5: Table S5**. Sequence summary of PacBio subreads.

**Additional file 6: Table S6**. Full length evaluation

**Additional file 7: Table S7**. Gene structure annotation

**Additional file 8: Table S8**. Illumina RNA-seq data of each stress.

**Additional file 9: Table S9**. Classification of FLNC sequences with genome alignment.

**Additional file 10: Table S10**. The list of Z141 and NY-17 DEGs under DS or RD

**Additional file 11: Figure S1**. Bubble diagram showing the GO classification of differentially expressed transcripts between DS and RD in Z141 or NY-17. (a, b) GO terms of downregulated genes overlapping between DS and RD in Z141 (a) or NY-17 (b). (c-f) GO terms of genes up- (c, d) or downregulated (e, f) in only Z141 under DS or RD, respectively. (g-j) GO terms of genes up- (g, h) or downregulated (i, j) in only NY-17 under DS or RD respectively.

**Additional file 12: Table S11** The GO analysis result of up- or down-regulated DEGs in Z141 and NY-17 under DS or RD stress.

**Additional file 13: Figure S2**. Bubble diagram showing the GO classification of differentially expressed transcripts between Z141 and NY-17 under DS or RD treatment. (a, b) GO terms of downregulated genes overlapping between Z141 and NY-17 under DS (a) or RD (b) treatment. (c-f) GO terms of genes up- (c, d) or downregulated (e, f) in Z141 or NY-17 under only DS. (g-j) GO terms of genes up- (g, h) or downregulated (i, j) in Z141 or NY-17 under only RD.

**Additional file 14: Table S12** The GO analysis result of DEGs both up- or down-regulated in Z141 and NY-17 under DS or RD stress.

**Additional file 15: Figure S3**. Tree diagram showing the REVIGO classification of up- or down-regulated differentially expressed transcripts in Z141 or NY-17 under DS or RD respectively. (a, b) The REVIGO classification of up- (a) and down-regulated (b) genes in Z141 under RD stress. (c, d) The REVIGO classification of up- (c) and down-regulated (d) genes in NY-17 under DS stress. (e, f) The REVIGO classification of up- (e) and down-regulated (f) genes in NY-17 under RD stress.

**Additional file 16: Figure S4**. MapMan visualization of drought stress-responsive DEGs in Z141 (b) and NY-17 (a, c) under DS and RD stress, respectively. The up- and downregulated DEGs are represented in red and blue colour. The Colour brightness indicates the degree of difference, as shown in the scale on the right.

**Additional file 17: Table S13**. Comparison of our predicated TFs with that released by PlantTFDB.

**Additional file 18: Table S14**. Detail lists of 15 clusters of differentially expressed transcription factors.

**Additional file 19: Table S15**. The results of candidate genes expression and functional analysis.

**Additional file 20: Table S16**. List of possible candidate genes selected for drought tolerance in linseed

**Additional file 21: Table S17**. All sample FPKM sheet (XLS 3860 kb)

**Additional file 22: Table S18**. All sample reads count sheet (ZIP 4362 kb) (XLS 3462 kb)

**Additional file 23: Table S19**. Primers designed for RT-PCR validation.

**Additional file 24: Figure S5**. The original and full-length gel images of Fig. 10. (XLS 3462 kb)

## Data Availability

The raw data has been uploaded to the National Center for Biotechnology Information Short Read Archive (https://www.ncbi.nlm.nih.gov/sra/PRJNA598287).

## Abbreviations

DS: Drought stress
RW: Re-watering
RD: Repeated drought
DEGs: Differentially expressed genes
GO: Gene ontology
RT-PCR: Reverse transcriptional RCR
ALA: α-linolenic acid
EPA: Eicosapentaenoic acid
DHA: Docosahexaenoic acid
SMRT: Single-molecule real-time
ASWC: Absolute soil water content
FL: Full-length
ALWC: Absolute leaf water content
RLWC: Relative leaf water content
CCS: Circular consensus
ROIs: Read of inserts
FLNC: Full-length and non-chimeric
DA: Drought avoidance
DT: Drought tolerance
