# Supplementary material for: Transcriptome analysis and molecular mechanism of linseed (Linum usitatissimum L.) drought tolerance under repeated drought using single-molecule long-read sequencing
Source: BMC Genomics. 2021 Feb 9;22:109. doi: 10.1186/s12864-021-07416-5 (PMC7871411; doi:10.1186/s12864-021-07416-5)
Supplement: Supplementary file 1 — Additional file 1: Table S1. Methodology for measuring the drought-tolerant related traits. [file 12864_2021_7416_MOESM1_ESM.docx]

Table S1. Methodology for measuring the drought-tolerant related traits

| **Trait** | **Measurement** |
| --- | --- |
| **Plant height (cm)** | Measured from the base of plants at the ground to the stem tip at seedling stage. |
| **Biomass (g)** | The aerial part of the plants is dried in an oven (~80 ℃) to constant weight |
| **LAWC (%)** | (fresh mass - dry mass)/ dry mass ×100% |
| **LRWC (%)** | (fresh mass- dry mass)/ (turgid mass- dry mass)× 100% |
