# Supplementary material for: Transcriptome analysis and molecular mechanism of linseed (Linum usitatissimum L.) drought tolerance under repeated drought using single-molecule long-read sequencing
Source: BMC Genomics. 2021 Feb 9;22:109. doi: 10.1186/s12864-021-07416-5 (PMC7871411; doi:10.1186/s12864-021-07416-5)
Supplement: Supplementary file 2 — Additional file 2: Table S2. Effect of drought stress (SAWS=10%) on drought tolerant related traits in Z141 and NY-17 [file 12864_2021_7416_MOESM2_ESM.docx]

Table S2. Effect of drought stress (SAWS=10%) on drought tolerant related traits in Z141 and NY-17.

|  | **Z141** | | **NY-17** | |
| --- | --- | --- | --- | --- |
| **Trait** | **Control** | **Drought** | **Control** | **Drought** |
| **Plant height (cm)** | 19.00±0.21 | 13.25±0.21 | 26.23±0.03 | 17.74±0.44 |
| **Biomass (g)** | 0.64±0.02 | 0.35±0.03 | 0.22±0.01 | 0.15±0.01 |
