# Supplementary material for: Transcriptome analysis and molecular mechanism of linseed (Linum usitatissimum L.) drought tolerance under repeated drought using single-molecule long-read sequencing
Source: BMC Genomics. 2021 Feb 9;22:109. doi: 10.1186/s12864-021-07416-5 (PMC7871411; doi:10.1186/s12864-021-07416-5)
Supplement: Supplementary file 3 — Additional file 3: Table S3. Effect of drought stress on ALWC and RLWC in Z141 and NY-17. [file 12864_2021_7416_MOESM3_ESM.docx]

Table S3. Effect of drought stress on LAWC and LRWC in Z141 and NY-17

|  | **Z141**  **ASWC (%)** | | | | | **NY-17**  **ASWC(%)** | | | | |
| --- | --- | --- | --- | --- | --- | --- | --- | --- | --- | --- |
| **Trait** | **70** | **50** | **30** | **10** | **0** | **70** | **50** | **30** | **10** | **0** |
| **ALWC (%)** | 87.50±0.14 | 85.22±0.18 | 84.22±0.22 | 81.13±0.37 | 72.32±1.10 | 86.17±0.19 | 86.23±0.59 | 84.25±0.32 | 83.05±0.31 | 68.34±0.80 |
| **RLWC (%)** | 75.99±0.46 | 73.45±1.35 | 72.40±1.81 | 60.54±0.58 | 31.81±0.51 | 76.23±1.60 | 70.91±2.36 | 74.49±0.93 | 56.09±0.52 | 28.74±0.77 |
