# Supplementary material for: Transcriptome analysis and molecular mechanism of linseed (Linum usitatissimum L.) drought tolerance under repeated drought using single-molecule long-read sequencing
Source: BMC Genomics. 2021 Feb 9;22:109. doi: 10.1186/s12864-021-07416-5 (PMC7871411; doi:10.1186/s12864-021-07416-5)
Supplement: Supplementary file 4 — Additional file 4: Table S4. Sequence summary of PacBio SMRT Cells. [file 12864_2021_7416_MOESM4_ESM.docx]

Table S4. Sequence summary of PacBio SMRT Cells

| **Library** | **Cell** | **Total basees (bp)** | **Reads Number** | **Mean Length (bp)** | **Read N50 (bp)** |
| --- | --- | --- | --- | --- | --- |
| Z141 1<3k | C01 | 12,903,767,113 | 472,461 | 27,312 | 49,640 |
| Z141 >3k | D01 | 15,358,282,321 | 652,592 | 23,534 | 40,804 |
| NY-17 1<3k | D01 | 16,863,639,348 | 559,270 | 30,153 | 53,923 |
| NY-17 >3k | H01 | 10,630,578,914 | 343,939 | 30,908 | 52,130 |
| Total |  | 55,756,267,696 | 2,028,262 |  |  |
