# Supplementary material for: Transcriptome analysis and molecular mechanism of linseed (Linum usitatissimum L.) drought tolerance under repeated drought using single-molecule long-read sequencing
Source: BMC Genomics. 2021 Feb 9;22:109. doi: 10.1186/s12864-021-07416-5 (PMC7871411; doi:10.1186/s12864-021-07416-5)
Supplement: Supplementary file 5 — Additional file 5: Table S5. Sequence summary of PacBio subreads. [file 12864_2021_7416_MOESM5_ESM.docx]

Table S5. Sequence summary of PacBio subreads.

| **Library** | **Cell** | **Total basees (bp)** | **NO. of subreads** | **Mean Subreaad Length (bp)** |
| --- | --- | --- | --- | --- |
| Z141 1<3k | C01 | 12,431,934,278 | 11,036,887 | 1,126 |
| Z141 >3k | D01 | 15,126,701,717 | 5,498,149 | 2,751 |
| NY-17 1<3k | D01 | 16,287,449,271 | 13,605,149 | 1,197 |
| NY-17 >3k | H01 | 10,513,142,382 | 3,020,688 | 3,480 |
| Total |  | 54,359,227,648 |  |  |
