# Supplementary material for: Transcriptome analysis and molecular mechanism of linseed (Linum usitatissimum L.) drought tolerance under repeated drought using single-molecule long-read sequencing
Source: BMC Genomics. 2021 Feb 9;22:109. doi: 10.1186/s12864-021-07416-5 (PMC7871411; doi:10.1186/s12864-021-07416-5)
Supplement: Supplementary file 6 — Additional file 6: Table S6. Full length evaluation [file 12864_2021_7416_MOESM6_ESM.docx]

Table S6. Full length evaluation

| **Sample** | **Library** | **Cell** | **# of CCS** | **5' primer** | **3' primer** | **Poly-A** | **Full-Length** | **# of FLNC** | **Mean FLNC Length(bp)** |
| --- | --- | --- | --- | --- | --- | --- | --- | --- | --- |
| Z141<3k | 1-3k | C01 | 388,621 | 372,744 | 372,381 | 365,490 | 353,313 | 348,808 | 1,163 |
| Z141>3k | 3k+ | D01 | 471,760 | 442,441 | 443,569 | 401,451 | 378,932 | 285,995 | 3,299 |
| NY-17<3k | 1-3k | D01 | 470,518 | 452,489 | 452,026 | 443,192 | 429,356 | 423,067 | 1,277 |
| NY-17>3k | 3k+ | H01 | 268,516 | 259,575 | 259,399 | 254,005 | 245,360 | 235,264 | 3,811 |
| Total | - | - | 1,599,415 | 1,527,249 | 1,527,375 | 1,464,138 | 1,406,961 | 1,293,134 | - |
