# Supplementary material for: Transcriptome analysis and molecular mechanism of linseed (Linum usitatissimum L.) drought tolerance under repeated drought using single-molecule long-read sequencing
Source: BMC Genomics. 2021 Feb 9;22:109. doi: 10.1186/s12864-021-07416-5 (PMC7871411; doi:10.1186/s12864-021-07416-5)
Supplement: Supplementary file 7 — Additional file 7: Table S7. Gene structure annotation [file 12864_2021_7416_MOESM7_ESM.docx]

Table S7. Gene structure annotation.

| Feature | Loci | Loci <1K | Loci 1-2K | Loci 2-3K | Loci >=3K |
| --- | --- | --- | --- | --- | --- |
| Annotation.loci | 43,484 | 22,026(50.65%) | 15,465(35.56%) | 4,119(9.47%) | 1,874(4.31%) |
| PacBio.loci | 28,686 | 4,304(15.00%) | 12,932(45.08%) | 5,137(17.91%) | 6,313(22.01%) |
