# Supplementary material for: Transcriptome analysis and molecular mechanism of linseed (Linum usitatissimum L.) drought tolerance under repeated drought using single-molecule long-read sequencing
Source: BMC Genomics. 2021 Feb 9;22:109. doi: 10.1186/s12864-021-07416-5 (PMC7871411; doi:10.1186/s12864-021-07416-5)
Supplement: Supplementary file 8 — Additional file 8: Table S8. Illumina RNA-seq data of each stress. [file 12864_2021_7416_MOESM8_ESM.docx]

Table S8. Illumina RNA-seq data of each stress.

| Sample | Total Reads | Total mapping rate | Left reads mapped | Left reads multiple mapped | Right reads mapped | Right reads multiple mapped |
| --- | --- | --- | --- | --- | --- | --- |
| z141-CK-1 | 46,611,198 | 41,250,992(88.5%) | 20,748,876(89.0%) | 620,466(3.0%) | 20,502,116(88.0%) | 608,496(3.0%) |
| z141-CK-2 | 42,135,638 | 37,104,476(88.1%) | 18,604,423(88.3%) | 480,228(2.6%) | 18,500,053(87.8%) | 475,817(2.6%) |
| z141-DS-1 | 38,108,244 | 33,333,847(87.5%) | 16,772,482(88.0%) | 474,619(2.8%) | 16,561,365(86.9%) | 467,027(2.8%) |
| z141-DS-2 | 43,607,676 | 37,517,971(86.0%) | 18,782,617(86.1%) | 546,373(2.9%) | 18,735,354(85.9%) | 546,559(2.9%) |
| z141-RW-1 | 40,664,846 | 35,139,155(86.4%) | 17,797,644(87.5%) | 816,005(4.6%) | 17,341,511(85.3%) | 785,701(4.5%) |
| z141-RW-2 | 41,738,502 | 35,375,400(84.8%) | 18,174,414(87.1%) | 815,141(4.5%) | 17,200,986(82.4%) | 757,114(4.4%) |
| z141-RD-1 | 48,517,024 | 41,924,285(86.4%) | 21,117,750(87.1%) | 567,022(2.7%) | 20,806,535(85.8%) | 565,001(2.7%) |
| z141-RD-2 | 41,681,234 | 35,361,967(84.8%) | 17,679,464(84.8%) | 483,765(2.7%) | 17,682,503(84.8%) | 486,816(2.8%) |
| NY17-CK-1 | 49,471,150 | 43,313,708(87.6%) | 21,801,450(88.1%) | 598,560(2.7%) | 21,512,258(87.0%) | 587,005(2.7%) |
| NY17-CK-2 | 49,885,912 | 44,166,031(88.5%) | 22,147,899(88.8%) | 616,686(2.8%) | 22,018,132(88.3%) | 609,831(2.8%) |
| NY17-DS-1 | 42,575,312 | 37,097,611(87.1%) | 18,574,715(87.3%) | 573,381(3.1%) | 18,522,896(87.0%) | 572,676(3.1%) |
| NY17-DS-2 | 50,269,748 | 44,424,566(88.4%) | 22,361,186(89.0%) | 642,218(2.9%) | 22,063,380(87.8%) | 631,256(2.9%) |
| NY17-RW-1 | 42,248,852 | 37,632,290(89.1%) | 18,878,334(89.4%) | 472,723(2.5%) | 18,753,956(88.8%) | 468,138(2.5%) |
| NY17-RW-2 | 39,559,908 | 34,367,100(86.9%) | 17,256,409(87.2%) | 448,232(2.6%) | 17,110,691(86.5%) | 444,370(2.6%) |
| NY17-RD-1 | 44,407,456 | 38,393,679(86.5%) | 19,289,002(86.9%) | 520,149(2.7%) | 19,104,677(86.0%) | 518,670(2.7%) |
| NY17-RD-2 | 50,412,482 | 44,583,922(88.4%) | 22,430,612(89.0%) | 545,864(2.4%) | 22,153,310(87.9%) | 538,218(2.4%) |
