# Supplementary material for: Transcriptome analysis and molecular mechanism of linseed (Linum usitatissimum L.) drought tolerance under repeated drought using single-molecule long-read sequencing
Source: BMC Genomics. 2021 Feb 9;22:109. doi: 10.1186/s12864-021-07416-5 (PMC7871411; doi:10.1186/s12864-021-07416-5)
Supplement: Supplementary file 9 — Additional file 9: Table S9. Classification of FLNC sequences with genome alignment. [file 12864_2021_7416_MOESM9_ESM.docx]

Table S9. Classification of FLNC sequences with genome alignment.

| feature | Pre correction | Post correctio | Merge |
| --- | --- | --- | --- |
| unmap | 8,248(0.64%) | 6,347(0.49%) | 5,837(0.45%) |
| multiple-best | 8,617(0.67%) | 8,693(0.67%) | 8,443(0.65%) |
| low pid | 294,844(22.80%) | 197,552(15.28%) | 185,568(14.35%) |
| high quality map | 981,421(75.89%) | 1,080,538(83.56%) | 1,093,282(84.55%) |

Note :

Multiple_best: both entire_PID and region_PID mapped to multiple site of genome.

low pid : entire_PID < 94%, region_PID<96%.

high quality map ：entire_PID >= 94%, region_PID >= 96%.

Merge: Integrating the results of post correction and before correction
