# Supplementary figures and images for: Transcriptome analysis and molecular mechanism of linseed (Linum usitatissimum L.) drought tolerance under repeated drought using single-molecule long-read sequencing
Source: BMC Genomics. 2021 Feb 9;22:109. doi: 10.1186/s12864-021-07416-5 (PMC7871411; doi:10.1186/s12864-021-07416-5)

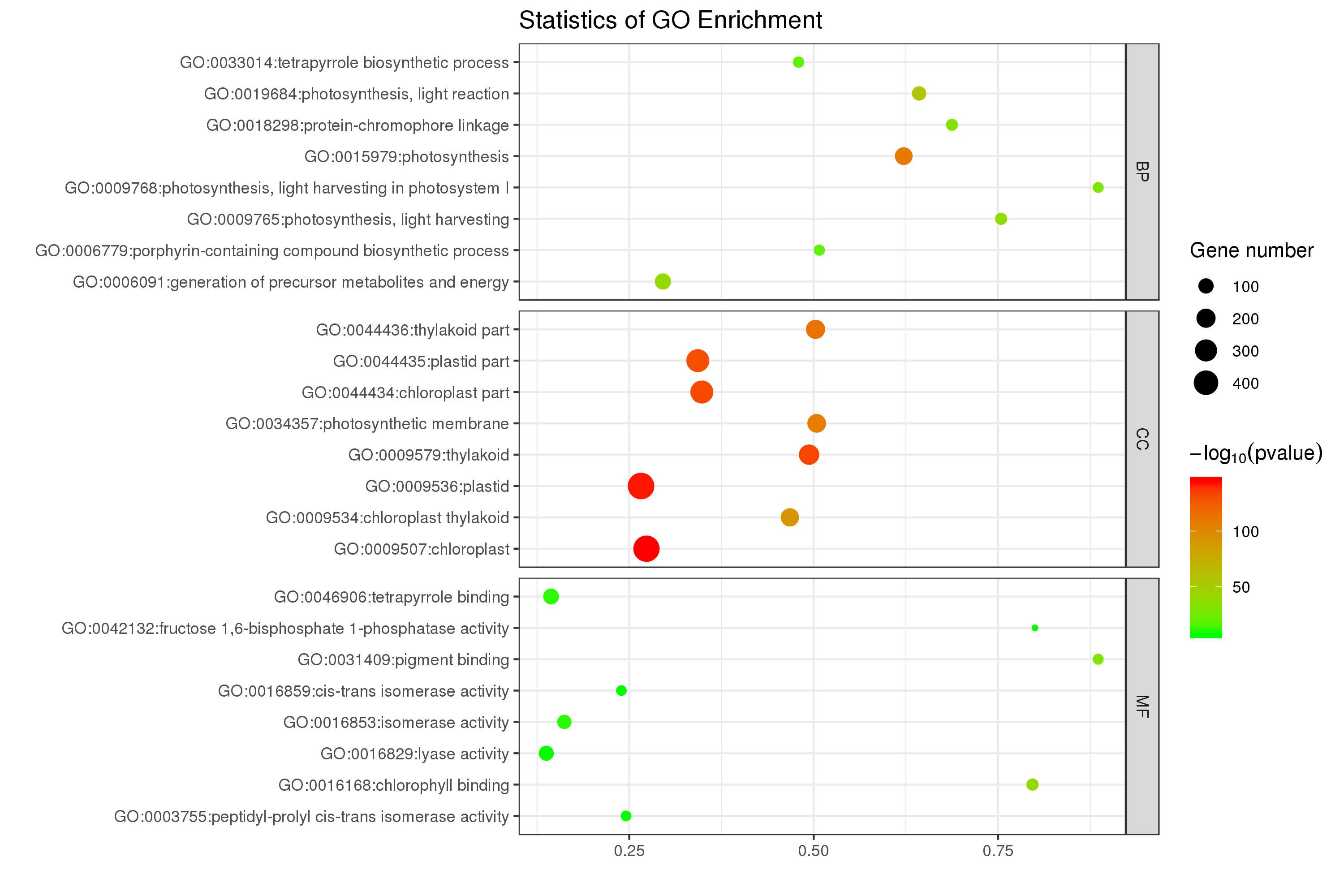

Supplement: Supplementary file 11 — Additional file 11: Figure S1. Bubble diagram showing the GO classification of differentially expressed transcripts between DS and RD in Z141 or NY-17. (a, b) GO terms of downregulated genes overlapping between DS and RD in Z141 (a) or NY-17 (b). (c-f) GO terms of genes up- (c, d) or downregulated (e, f) in only Z141 under DS or RD, respectively. (g-j) GO terms of genes up- (g, h) or downregulated (i, j) in only NY-17 under DS or RD respectively. [file 12864_2021_7416_MOESM11_ESM.zip › Supplementary Figure S1A.png]

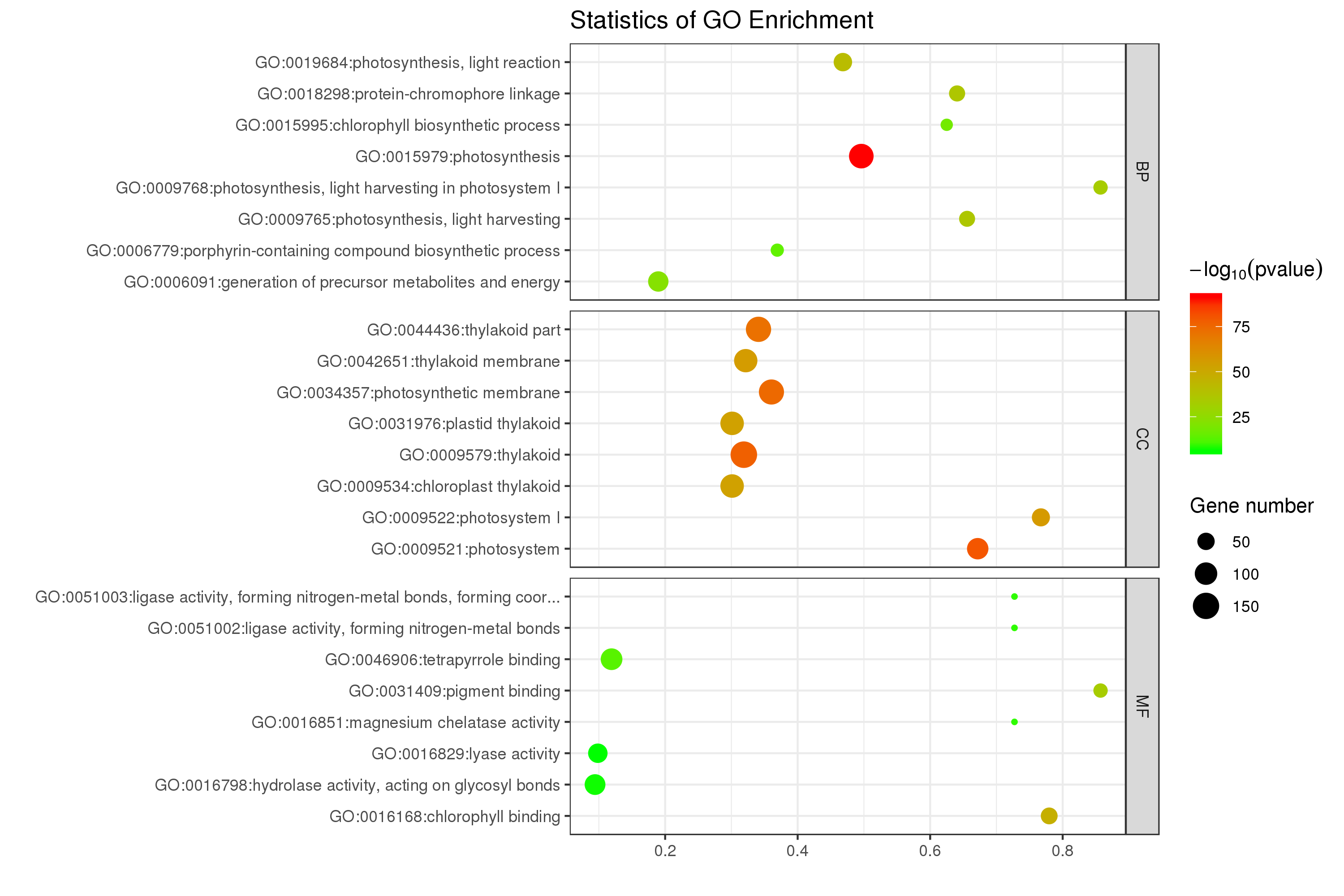

Supplement: Supplementary file 11 — Additional file 11: Figure S1. Bubble diagram showing the GO classification of differentially expressed transcripts between DS and RD in Z141 or NY-17. (a, b) GO terms of downregulated genes overlapping between DS and RD in Z141 (a) or NY-17 (b). (c-f) GO terms of genes up- (c, d) or downregulated (e, f) in only Z141 under DS or RD, respectively. (g-j) GO terms of genes up- (g, h) or downregulated (i, j) in only NY-17 under DS or RD respectively. [file 12864_2021_7416_MOESM11_ESM.zip › Supplementary Figure S1B.png]

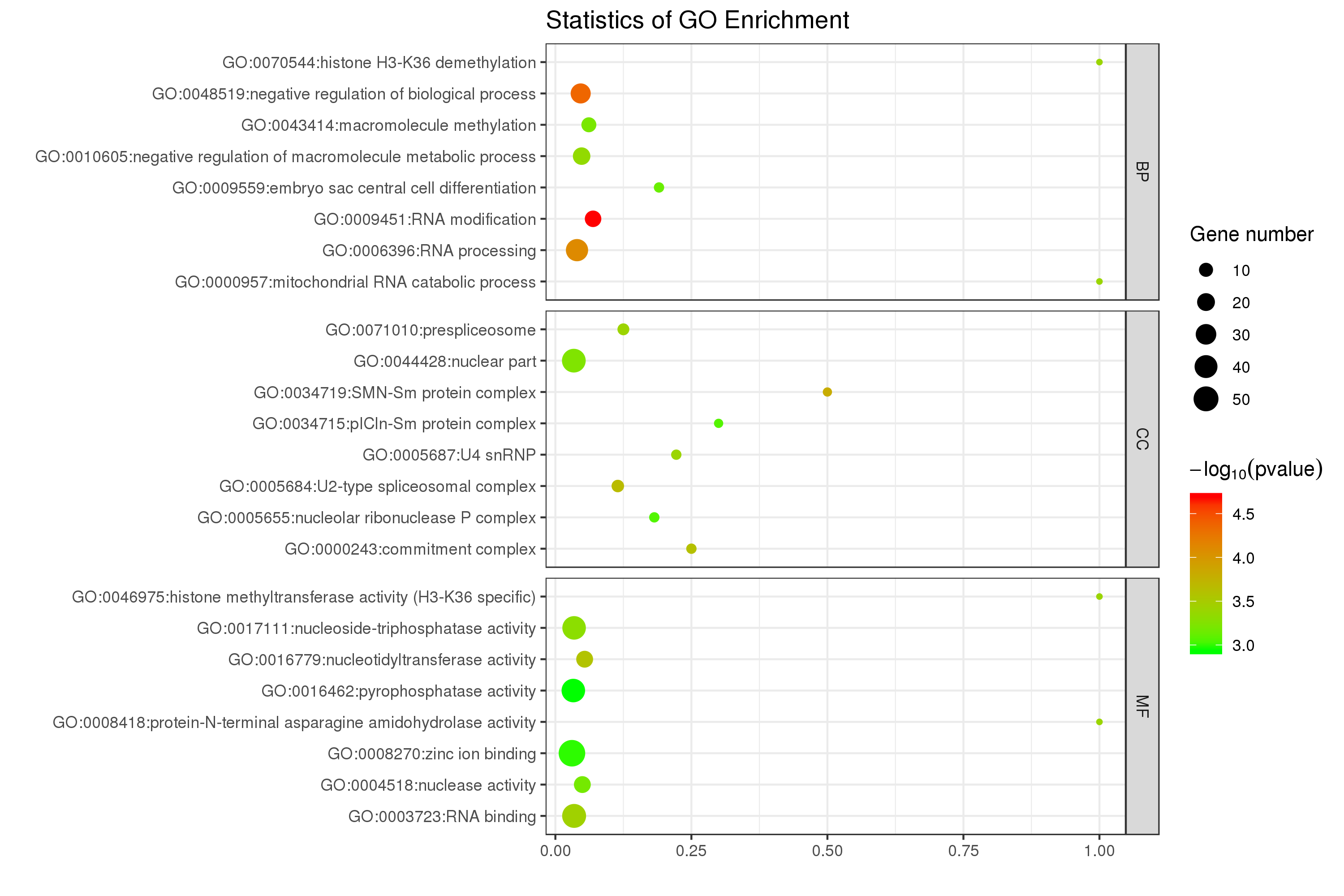

Supplement: Supplementary file 11 — Additional file 11: Figure S1. Bubble diagram showing the GO classification of differentially expressed transcripts between DS and RD in Z141 or NY-17. (a, b) GO terms of downregulated genes overlapping between DS and RD in Z141 (a) or NY-17 (b). (c-f) GO terms of genes up- (c, d) or downregulated (e, f) in only Z141 under DS or RD, respectively. (g-j) GO terms of genes up- (g, h) or downregulated (i, j) in only NY-17 under DS or RD respectively. [file 12864_2021_7416_MOESM11_ESM.zip › Supplementary Figure S1C.png]

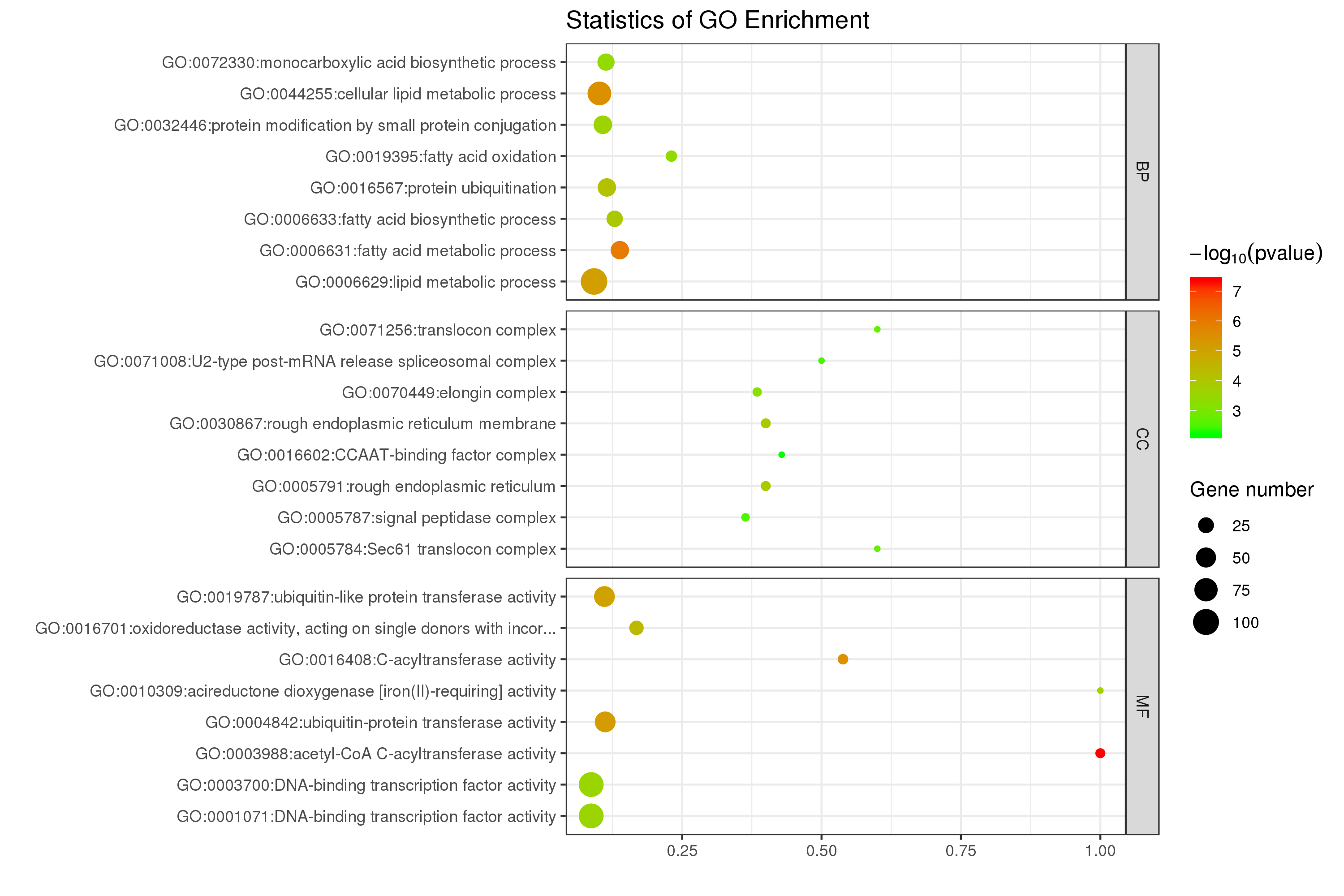

Supplement: Supplementary file 11 — Additional file 11: Figure S1. Bubble diagram showing the GO classification of differentially expressed transcripts between DS and RD in Z141 or NY-17. (a, b) GO terms of downregulated genes overlapping between DS and RD in Z141 (a) or NY-17 (b). (c-f) GO terms of genes up- (c, d) or downregulated (e, f) in only Z141 under DS or RD, respectively. (g-j) GO terms of genes up- (g, h) or downregulated (i, j) in only NY-17 under DS or RD respectively. [file 12864_2021_7416_MOESM11_ESM.zip › Supplementary Figure S1D.png]

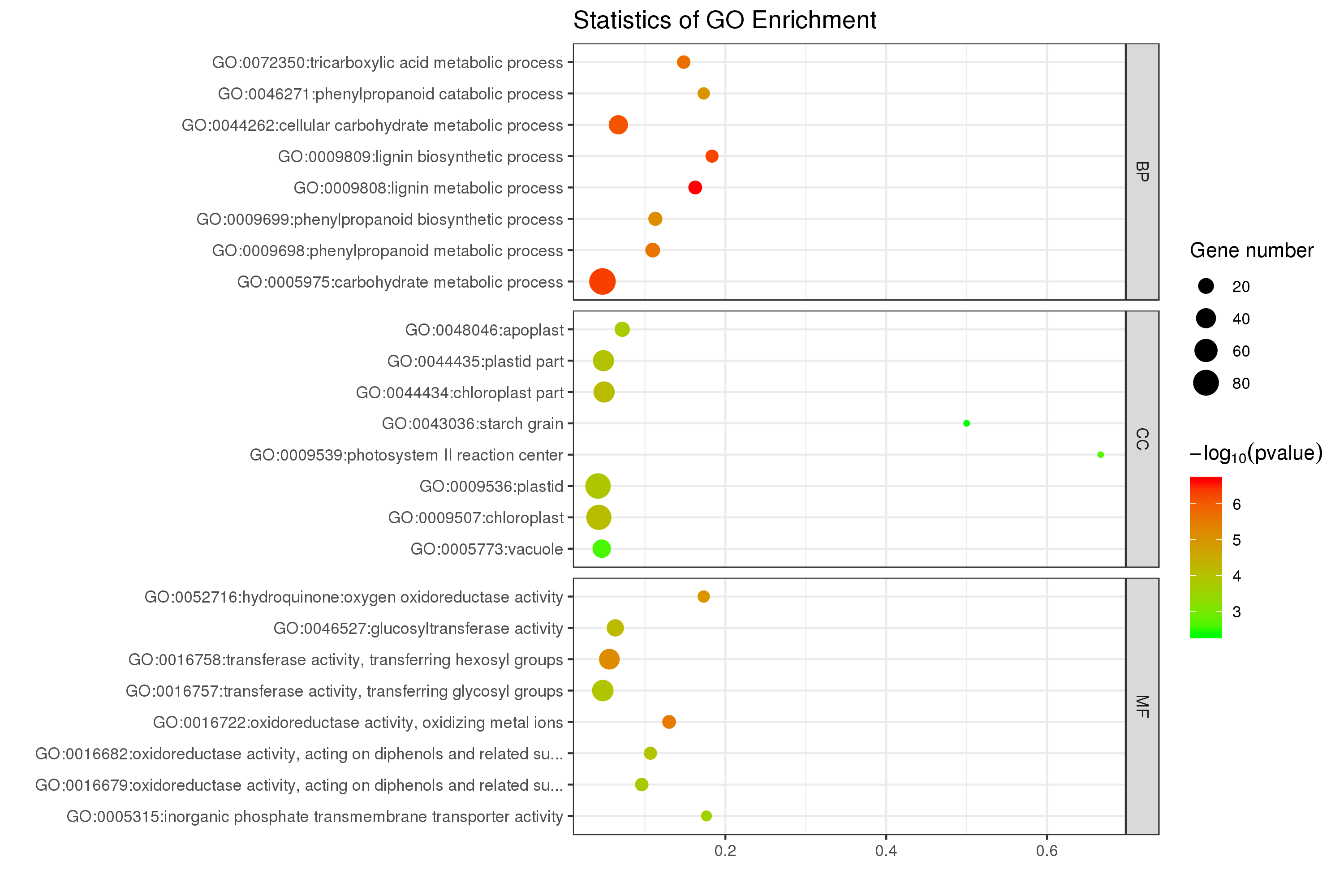

Supplement: Supplementary file 11 — Additional file 11: Figure S1. Bubble diagram showing the GO classification of differentially expressed transcripts between DS and RD in Z141 or NY-17. (a, b) GO terms of downregulated genes overlapping between DS and RD in Z141 (a) or NY-17 (b). (c-f) GO terms of genes up- (c, d) or downregulated (e, f) in only Z141 under DS or RD, respectively. (g-j) GO terms of genes up- (g, h) or downregulated (i, j) in only NY-17 under DS or RD respectively. [file 12864_2021_7416_MOESM11_ESM.zip › Supplementary Figure S1E.png]

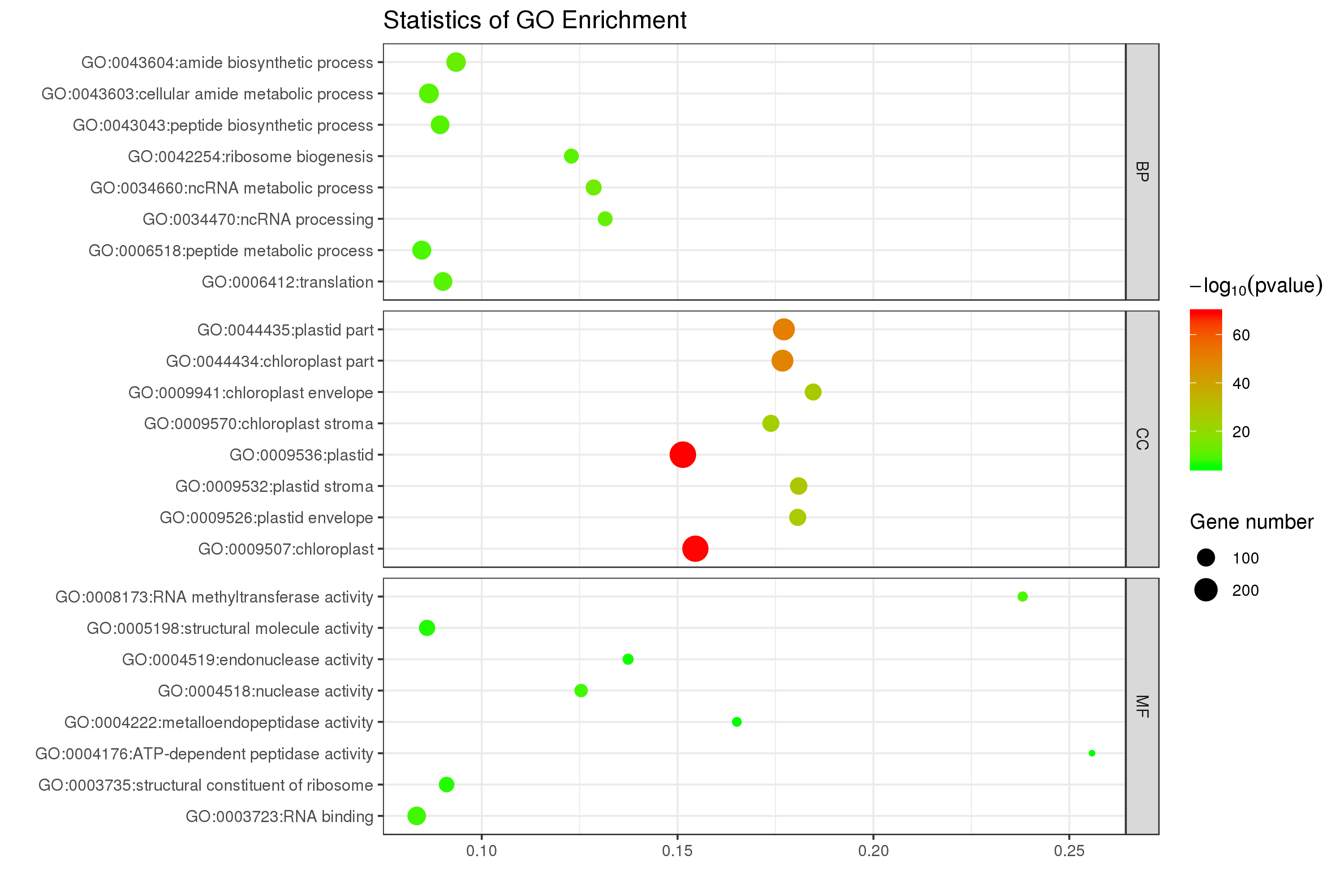

Supplement: Supplementary file 11 — Additional file 11: Figure S1. Bubble diagram showing the GO classification of differentially expressed transcripts between DS and RD in Z141 or NY-17. (a, b) GO terms of downregulated genes overlapping between DS and RD in Z141 (a) or NY-17 (b). (c-f) GO terms of genes up- (c, d) or downregulated (e, f) in only Z141 under DS or RD, respectively. (g-j) GO terms of genes up- (g, h) or downregulated (i, j) in only NY-17 under DS or RD respectively. [file 12864_2021_7416_MOESM11_ESM.zip › Supplementary Figure S1F.png]

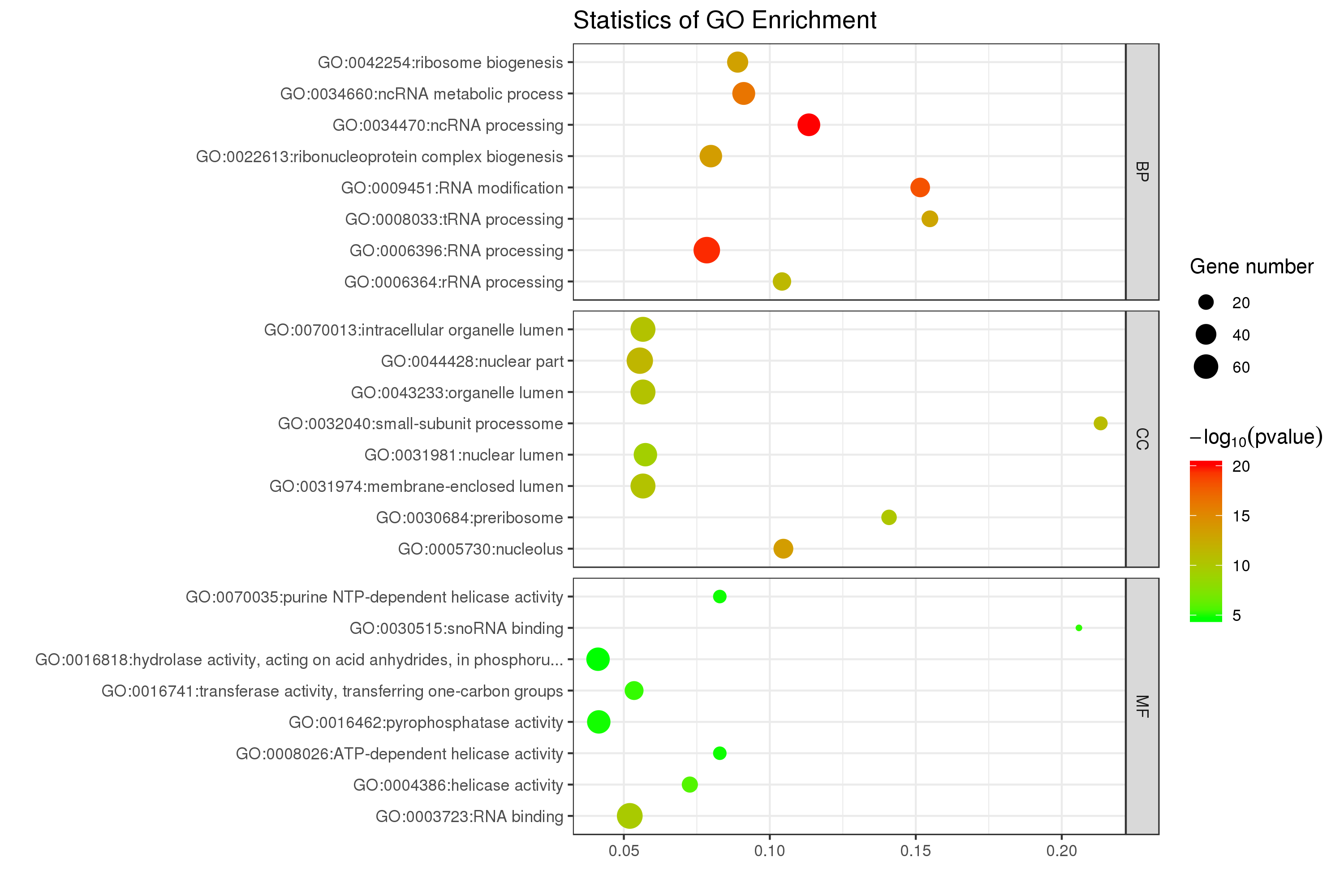

Supplement: Supplementary file 11 — Additional file 11: Figure S1. Bubble diagram showing the GO classification of differentially expressed transcripts between DS and RD in Z141 or NY-17. (a, b) GO terms of downregulated genes overlapping between DS and RD in Z141 (a) or NY-17 (b). (c-f) GO terms of genes up- (c, d) or downregulated (e, f) in only Z141 under DS or RD, respectively. (g-j) GO terms of genes up- (g, h) or downregulated (i, j) in only NY-17 under DS or RD respectively. [file 12864_2021_7416_MOESM11_ESM.zip › Supplementary Figure S1G.png]

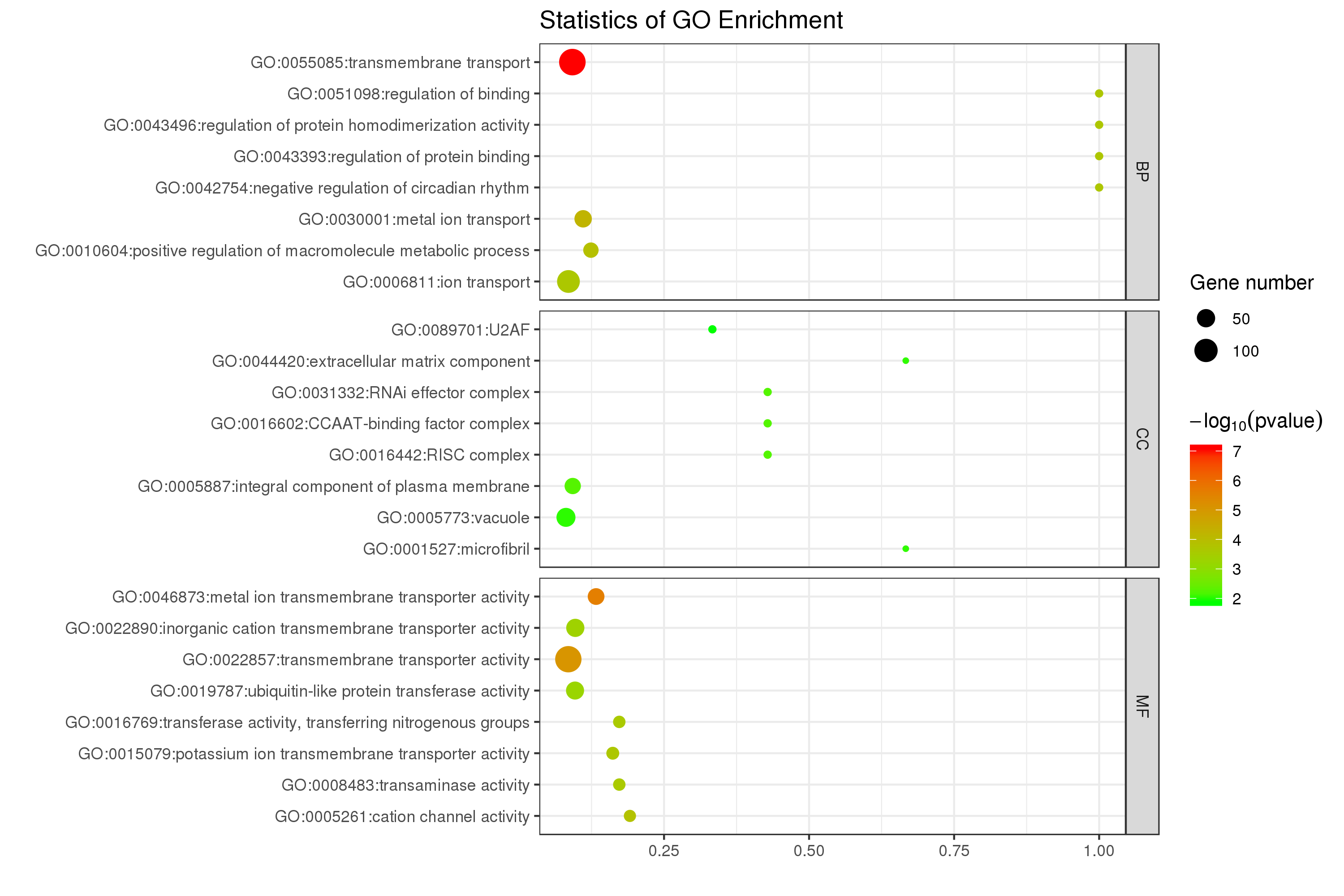

Supplement: Supplementary file 11 — Additional file 11: Figure S1. Bubble diagram showing the GO classification of differentially expressed transcripts between DS and RD in Z141 or NY-17. (a, b) GO terms of downregulated genes overlapping between DS and RD in Z141 (a) or NY-17 (b). (c-f) GO terms of genes up- (c, d) or downregulated (e, f) in only Z141 under DS or RD, respectively. (g-j) GO terms of genes up- (g, h) or downregulated (i, j) in only NY-17 under DS or RD respectively. [file 12864_2021_7416_MOESM11_ESM.zip › Supplementary Figure S1H.png]

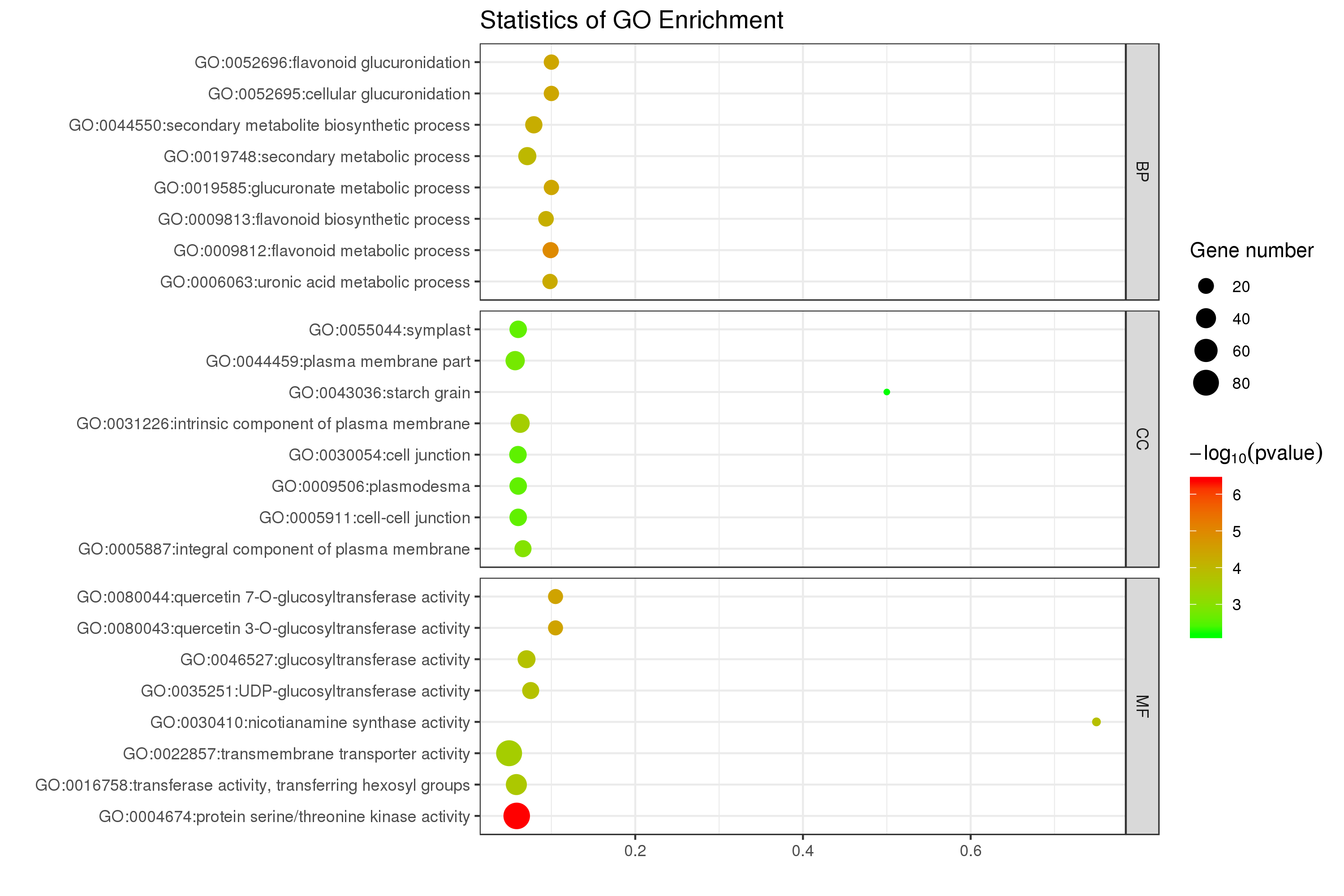

Supplement: Supplementary file 11 — Additional file 11: Figure S1. Bubble diagram showing the GO classification of differentially expressed transcripts between DS and RD in Z141 or NY-17. (a, b) GO terms of downregulated genes overlapping between DS and RD in Z141 (a) or NY-17 (b). (c-f) GO terms of genes up- (c, d) or downregulated (e, f) in only Z141 under DS or RD, respectively. (g-j) GO terms of genes up- (g, h) or downregulated (i, j) in only NY-17 under DS or RD respectively. [file 12864_2021_7416_MOESM11_ESM.zip › Supplementary Figure S1I.png]

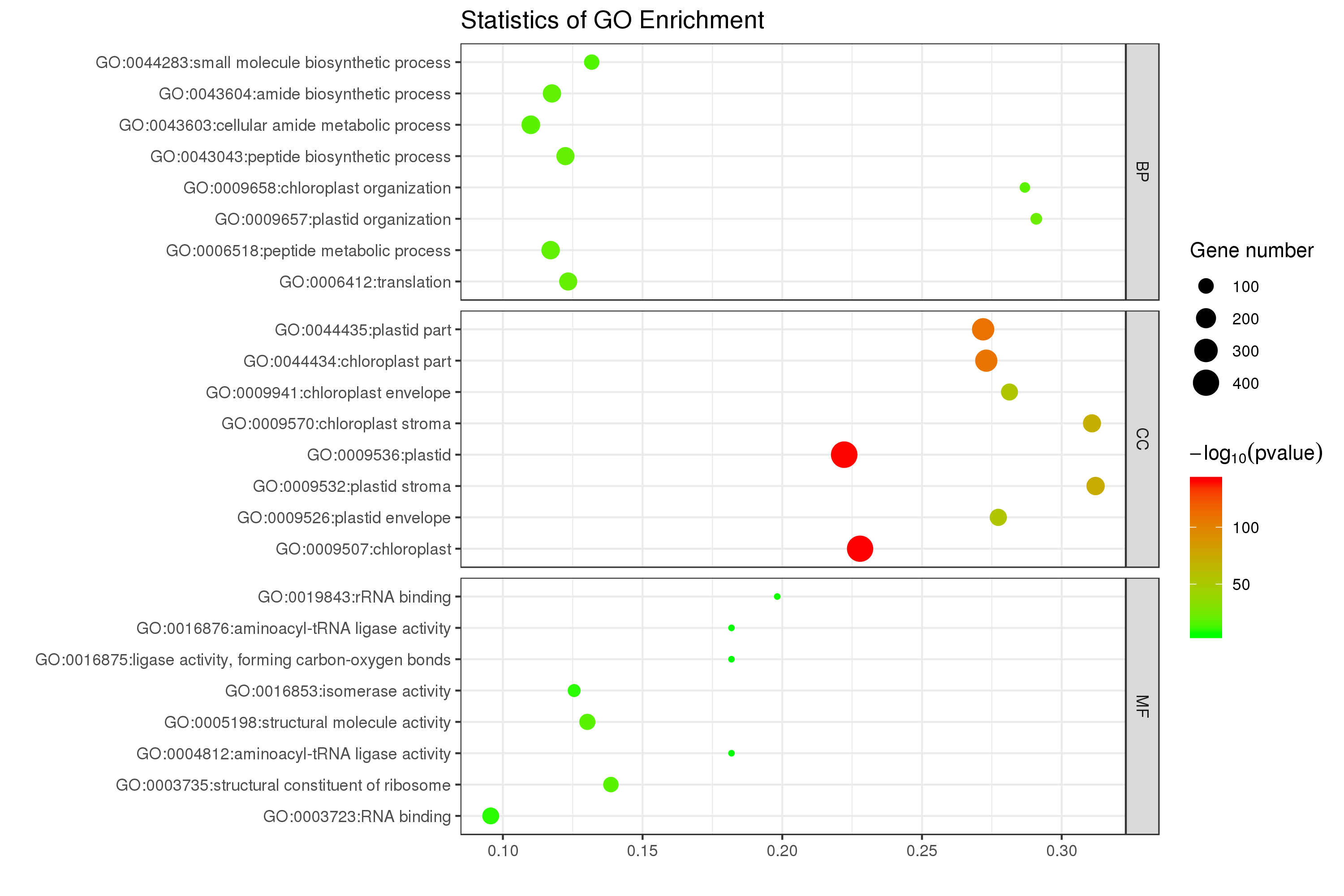

Supplement: Supplementary file 11 — Additional file 11: Figure S1. Bubble diagram showing the GO classification of differentially expressed transcripts between DS and RD in Z141 or NY-17. (a, b) GO terms of downregulated genes overlapping between DS and RD in Z141 (a) or NY-17 (b). (c-f) GO terms of genes up- (c, d) or downregulated (e, f) in only Z141 under DS or RD, respectively. (g-j) GO terms of genes up- (g, h) or downregulated (i, j) in only NY-17 under DS or RD respectively. [file 12864_2021_7416_MOESM11_ESM.zip › Supplementary Figure S1J.png]

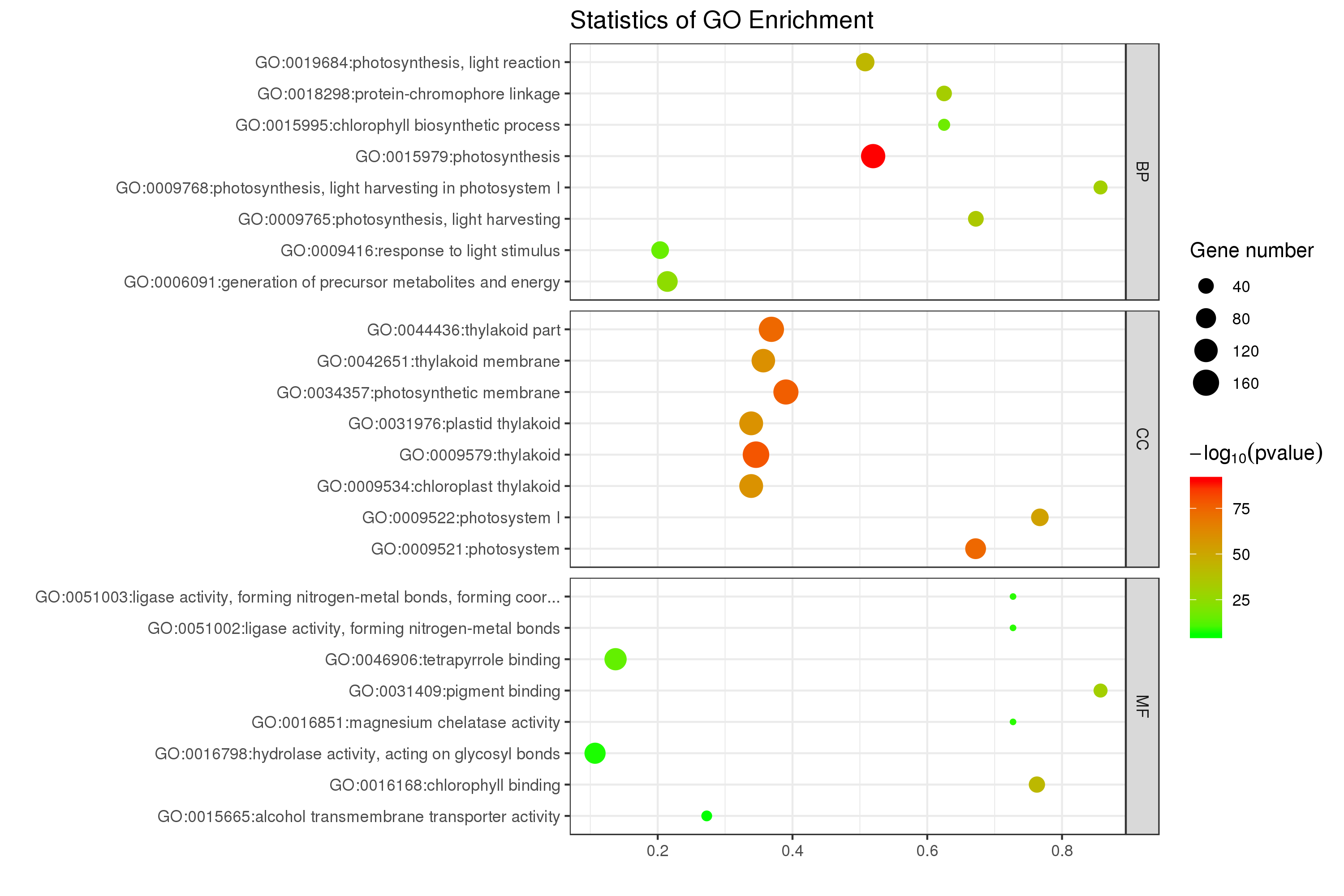

Supplement: Supplementary file 13 — Additional file 13: Figure S2. Bubble diagram showing the GO classification of differentially expressed transcripts between Z141 and NY-17 under DS or RD treatment. (a, b) GO terms of downregulated genes overlapping between Z141 and NY-17 under DS (a) or RD (b) treatment. (c-f) GO terms of genes up- (c, d) or downregulated (e, f) in Z141 or NY-17 under only DS. (g-j) GO terms of genes up- (g, h) or downregulated (i, j) in Z141 or NY-17 under only RD. [file 12864_2021_7416_MOESM13_ESM.zip › Supplementary Figure S2A.png]

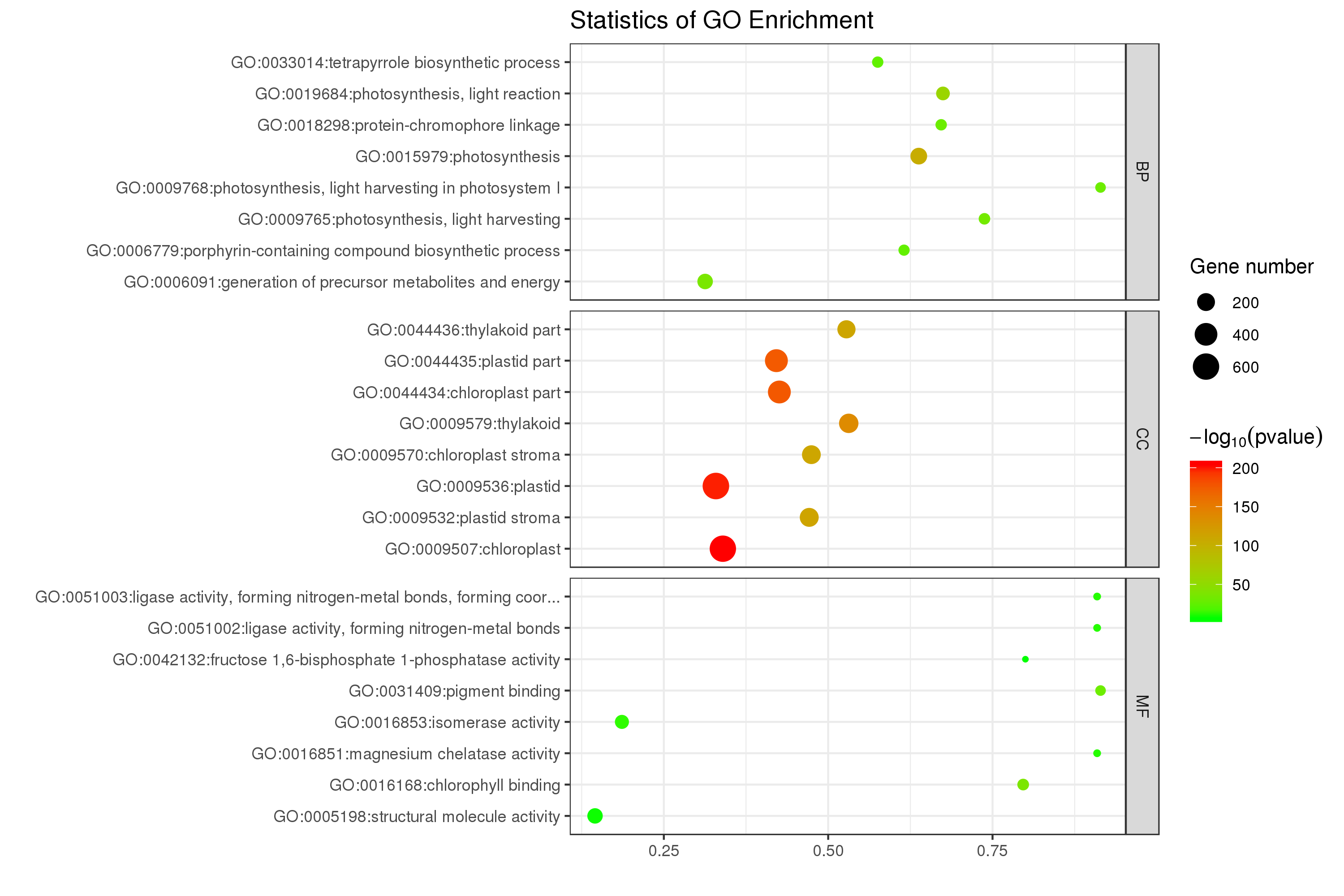

Supplement: Supplementary file 13 — Additional file 13: Figure S2. Bubble diagram showing the GO classification of differentially expressed transcripts between Z141 and NY-17 under DS or RD treatment. (a, b) GO terms of downregulated genes overlapping between Z141 and NY-17 under DS (a) or RD (b) treatment. (c-f) GO terms of genes up- (c, d) or downregulated (e, f) in Z141 or NY-17 under only DS. (g-j) GO terms of genes up- (g, h) or downregulated (i, j) in Z141 or NY-17 under only RD. [file 12864_2021_7416_MOESM13_ESM.zip › Supplementary Figure S2B.png]

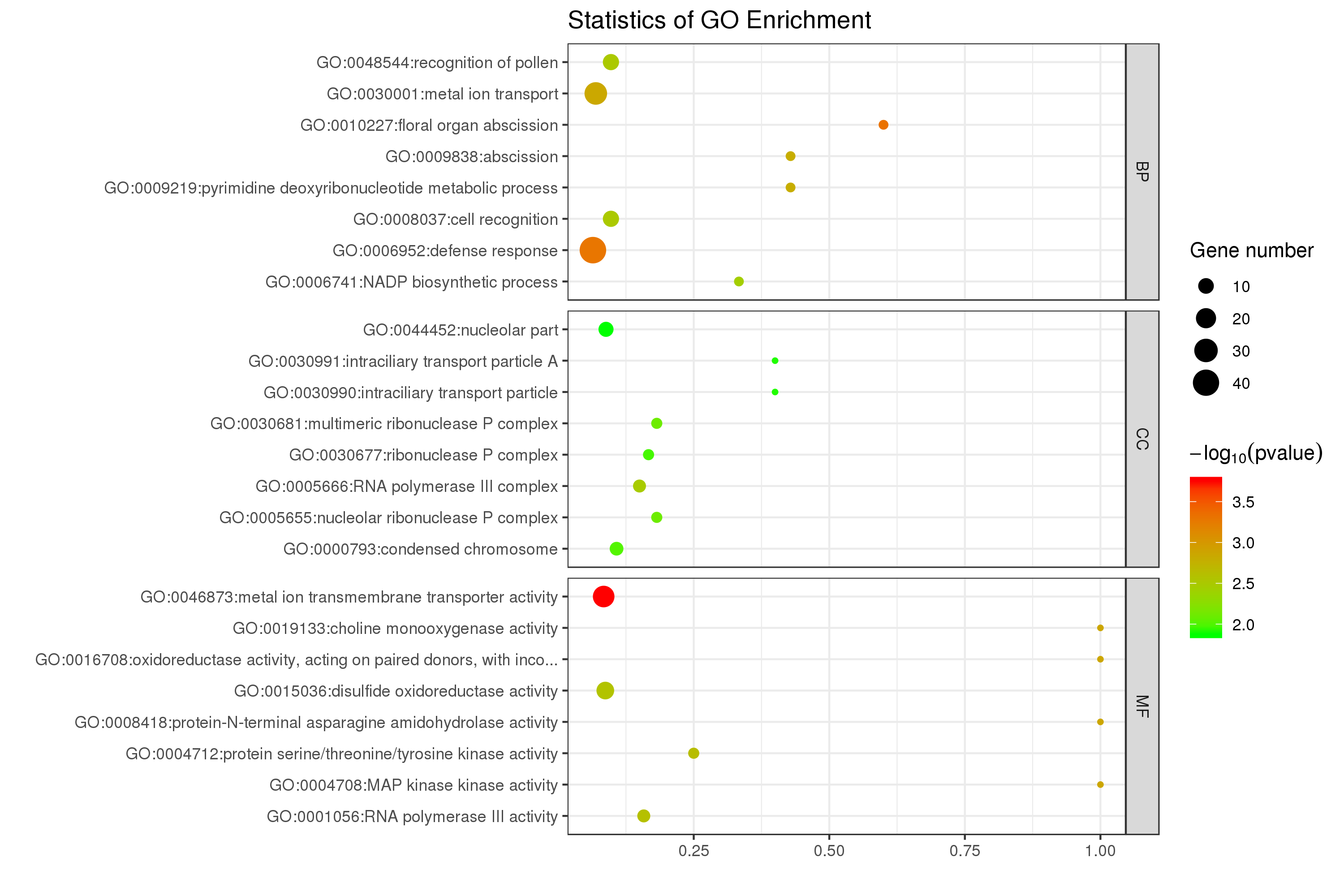

Supplement: Supplementary file 13 — Additional file 13: Figure S2. Bubble diagram showing the GO classification of differentially expressed transcripts between Z141 and NY-17 under DS or RD treatment. (a, b) GO terms of downregulated genes overlapping between Z141 and NY-17 under DS (a) or RD (b) treatment. (c-f) GO terms of genes up- (c, d) or downregulated (e, f) in Z141 or NY-17 under only DS. (g-j) GO terms of genes up- (g, h) or downregulated (i, j) in Z141 or NY-17 under only RD. [file 12864_2021_7416_MOESM13_ESM.zip › Supplementary Figure S2C.png]

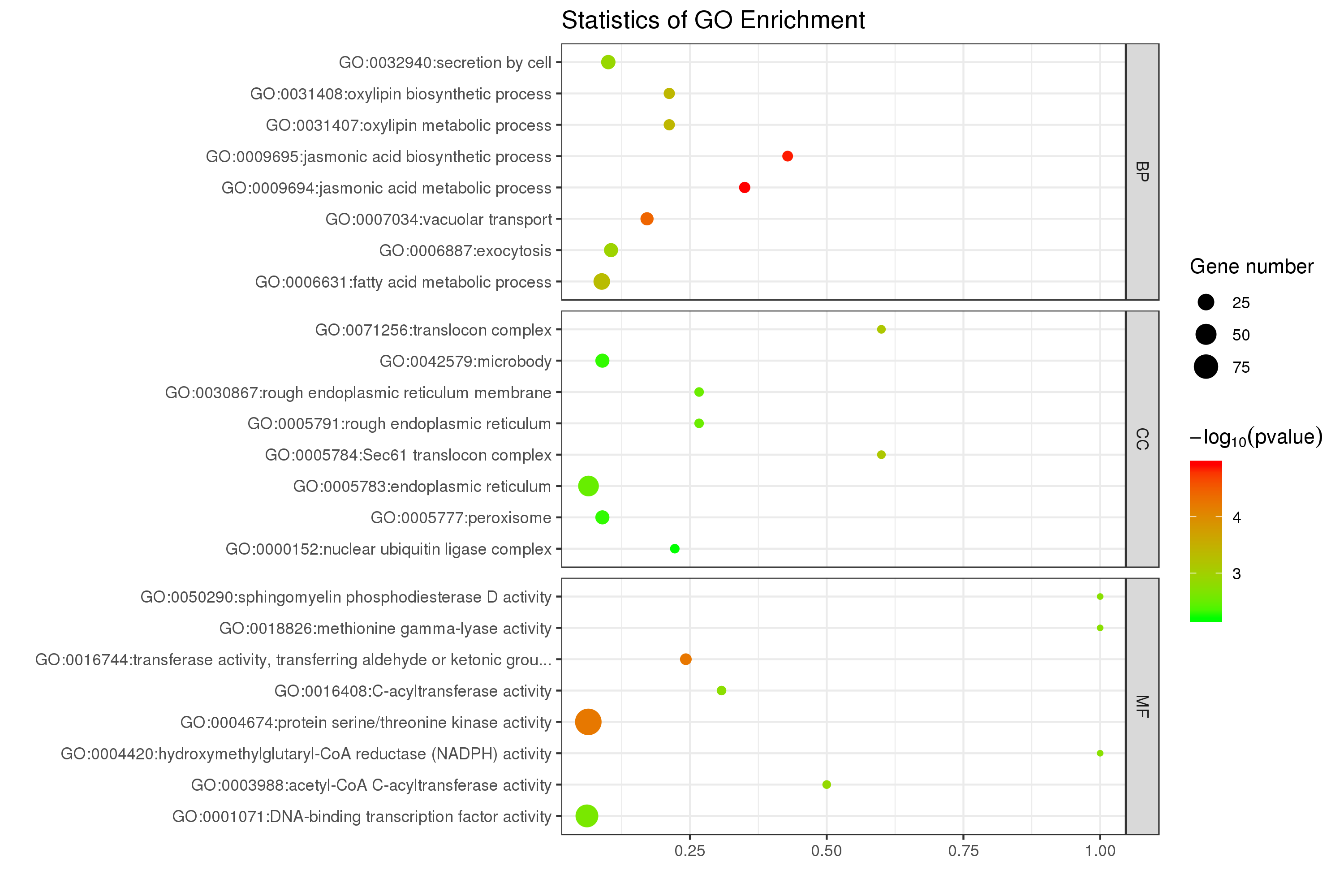

Supplement: Supplementary file 13 — Additional file 13: Figure S2. Bubble diagram showing the GO classification of differentially expressed transcripts between Z141 and NY-17 under DS or RD treatment. (a, b) GO terms of downregulated genes overlapping between Z141 and NY-17 under DS (a) or RD (b) treatment. (c-f) GO terms of genes up- (c, d) or downregulated (e, f) in Z141 or NY-17 under only DS. (g-j) GO terms of genes up- (g, h) or downregulated (i, j) in Z141 or NY-17 under only RD. [file 12864_2021_7416_MOESM13_ESM.zip › Supplementary Figure S2D.png]

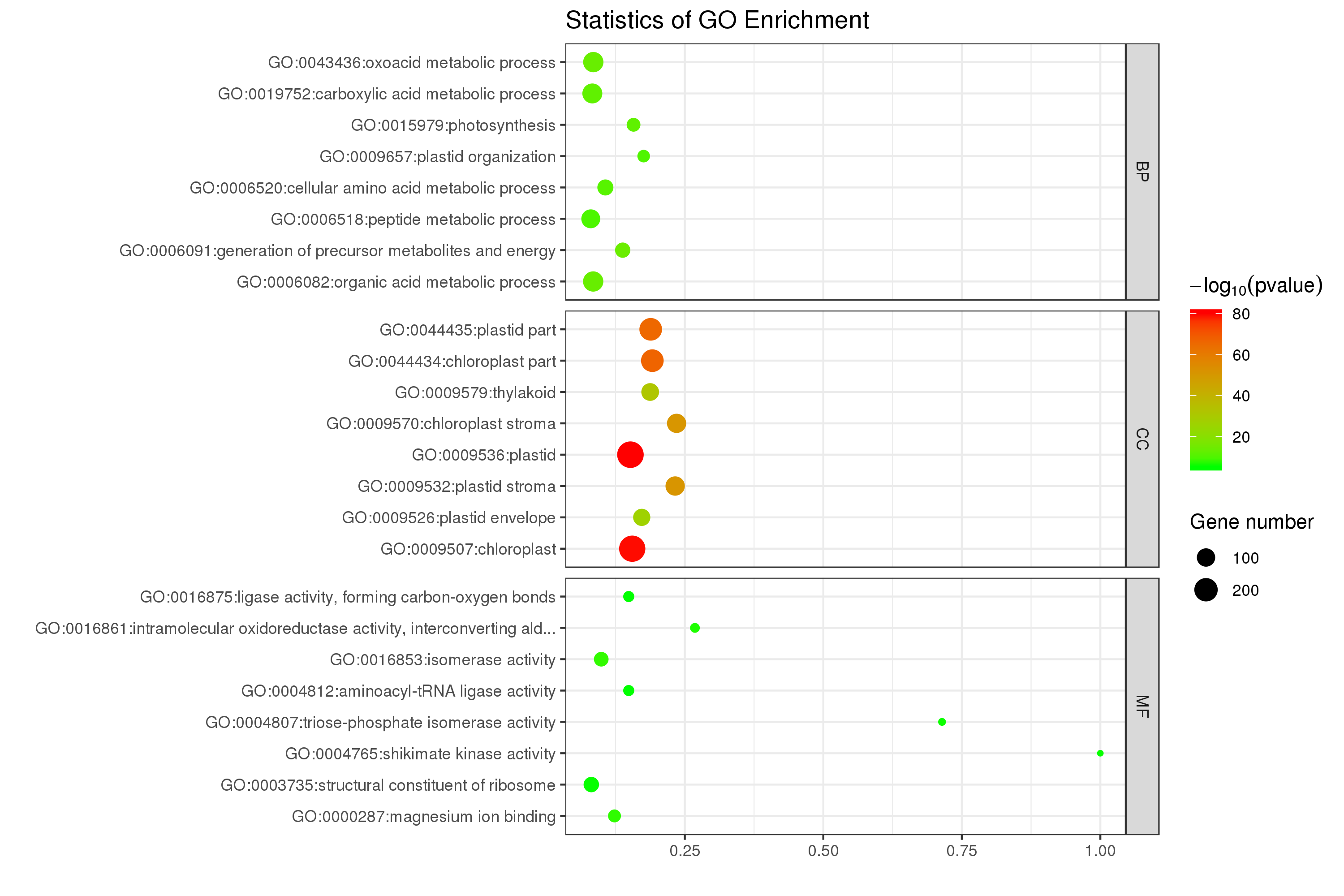

Supplement: Supplementary file 13 — Additional file 13: Figure S2. Bubble diagram showing the GO classification of differentially expressed transcripts between Z141 and NY-17 under DS or RD treatment. (a, b) GO terms of downregulated genes overlapping between Z141 and NY-17 under DS (a) or RD (b) treatment. (c-f) GO terms of genes up- (c, d) or downregulated (e, f) in Z141 or NY-17 under only DS. (g-j) GO terms of genes up- (g, h) or downregulated (i, j) in Z141 or NY-17 under only RD. [file 12864_2021_7416_MOESM13_ESM.zip › Supplementary Figure S2E.png]

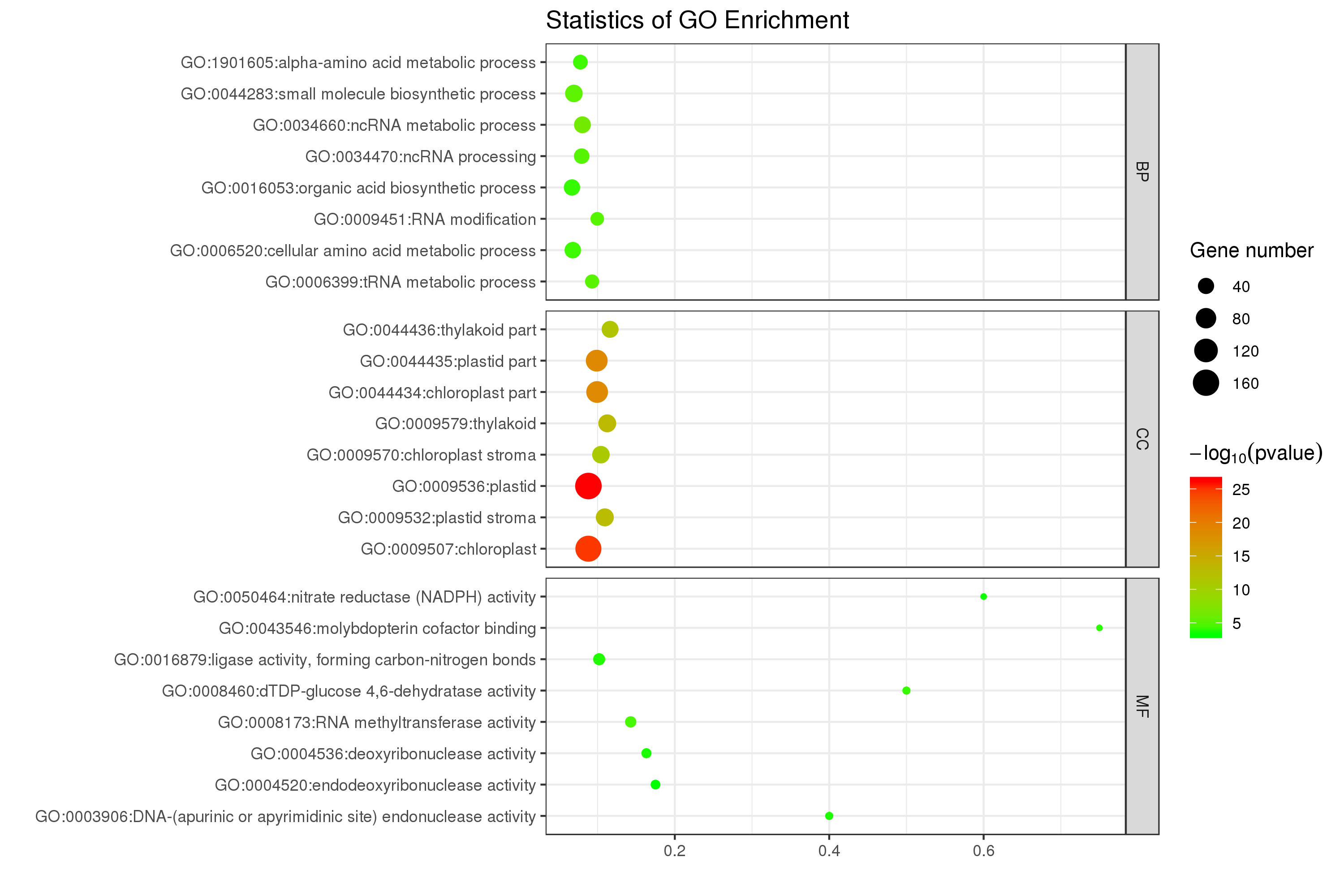

Supplement: Supplementary file 13 — Additional file 13: Figure S2. Bubble diagram showing the GO classification of differentially expressed transcripts between Z141 and NY-17 under DS or RD treatment. (a, b) GO terms of downregulated genes overlapping between Z141 and NY-17 under DS (a) or RD (b) treatment. (c-f) GO terms of genes up- (c, d) or downregulated (e, f) in Z141 or NY-17 under only DS. (g-j) GO terms of genes up- (g, h) or downregulated (i, j) in Z141 or NY-17 under only RD. [file 12864_2021_7416_MOESM13_ESM.zip › Supplementary Figure S2F.png]

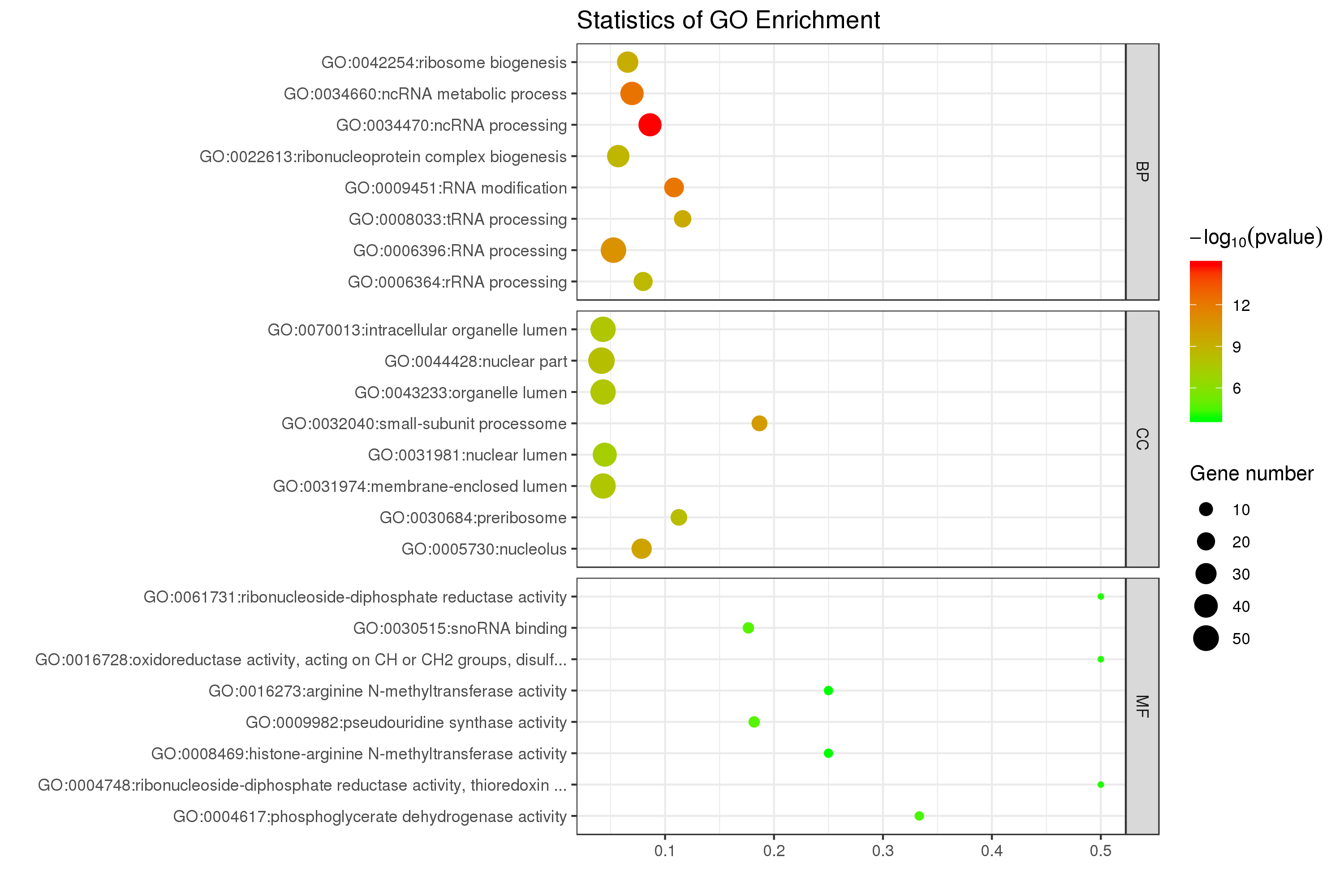

Supplement: Supplementary file 13 — Additional file 13: Figure S2. Bubble diagram showing the GO classification of differentially expressed transcripts between Z141 and NY-17 under DS or RD treatment. (a, b) GO terms of downregulated genes overlapping between Z141 and NY-17 under DS (a) or RD (b) treatment. (c-f) GO terms of genes up- (c, d) or downregulated (e, f) in Z141 or NY-17 under only DS. (g-j) GO terms of genes up- (g, h) or downregulated (i, j) in Z141 or NY-17 under only RD. [file 12864_2021_7416_MOESM13_ESM.zip › Supplementary Figure S2G.png]

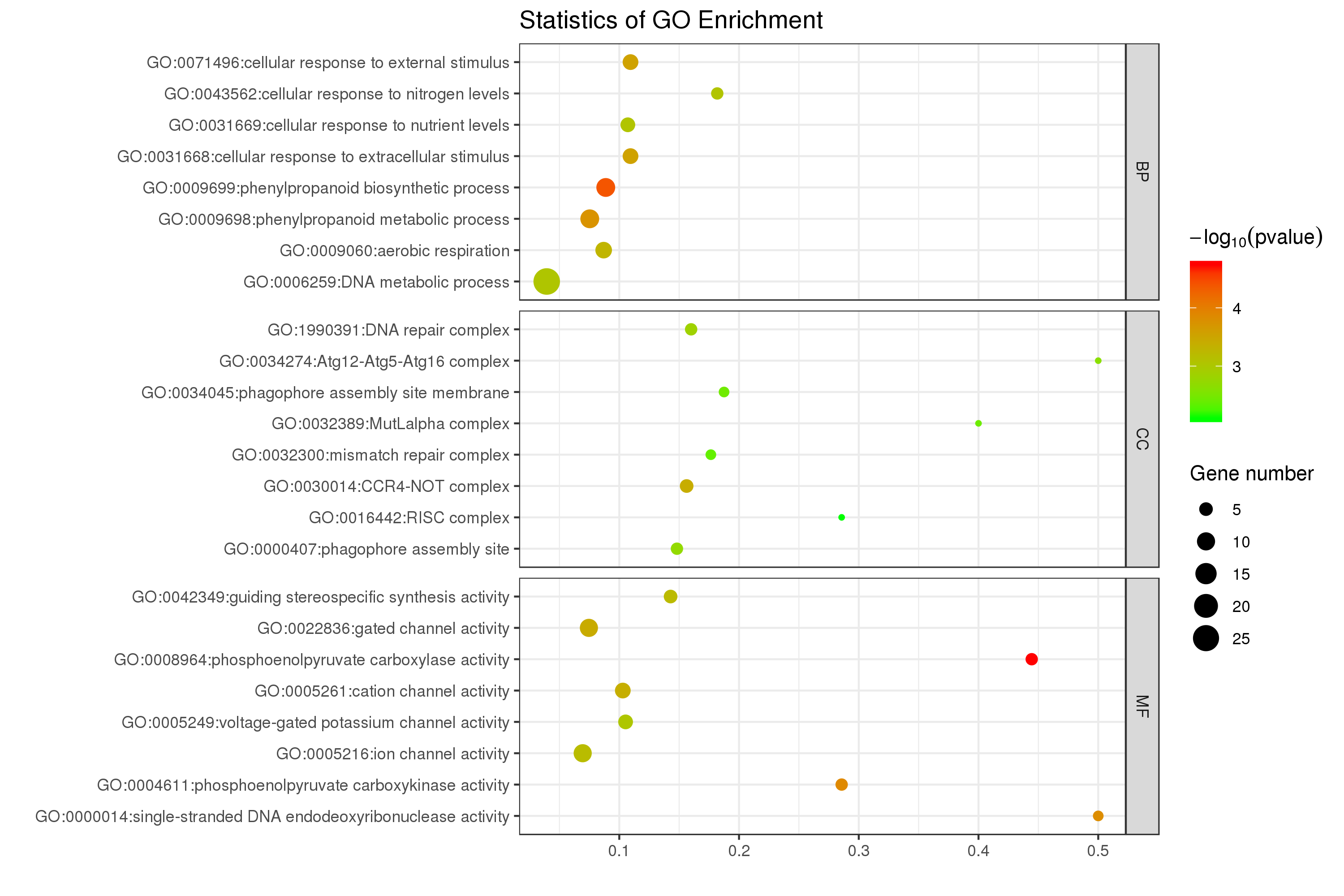

Supplement: Supplementary file 13 — Additional file 13: Figure S2. Bubble diagram showing the GO classification of differentially expressed transcripts between Z141 and NY-17 under DS or RD treatment. (a, b) GO terms of downregulated genes overlapping between Z141 and NY-17 under DS (a) or RD (b) treatment. (c-f) GO terms of genes up- (c, d) or downregulated (e, f) in Z141 or NY-17 under only DS. (g-j) GO terms of genes up- (g, h) or downregulated (i, j) in Z141 or NY-17 under only RD. [file 12864_2021_7416_MOESM13_ESM.zip › Supplementary Figure S2H.png]

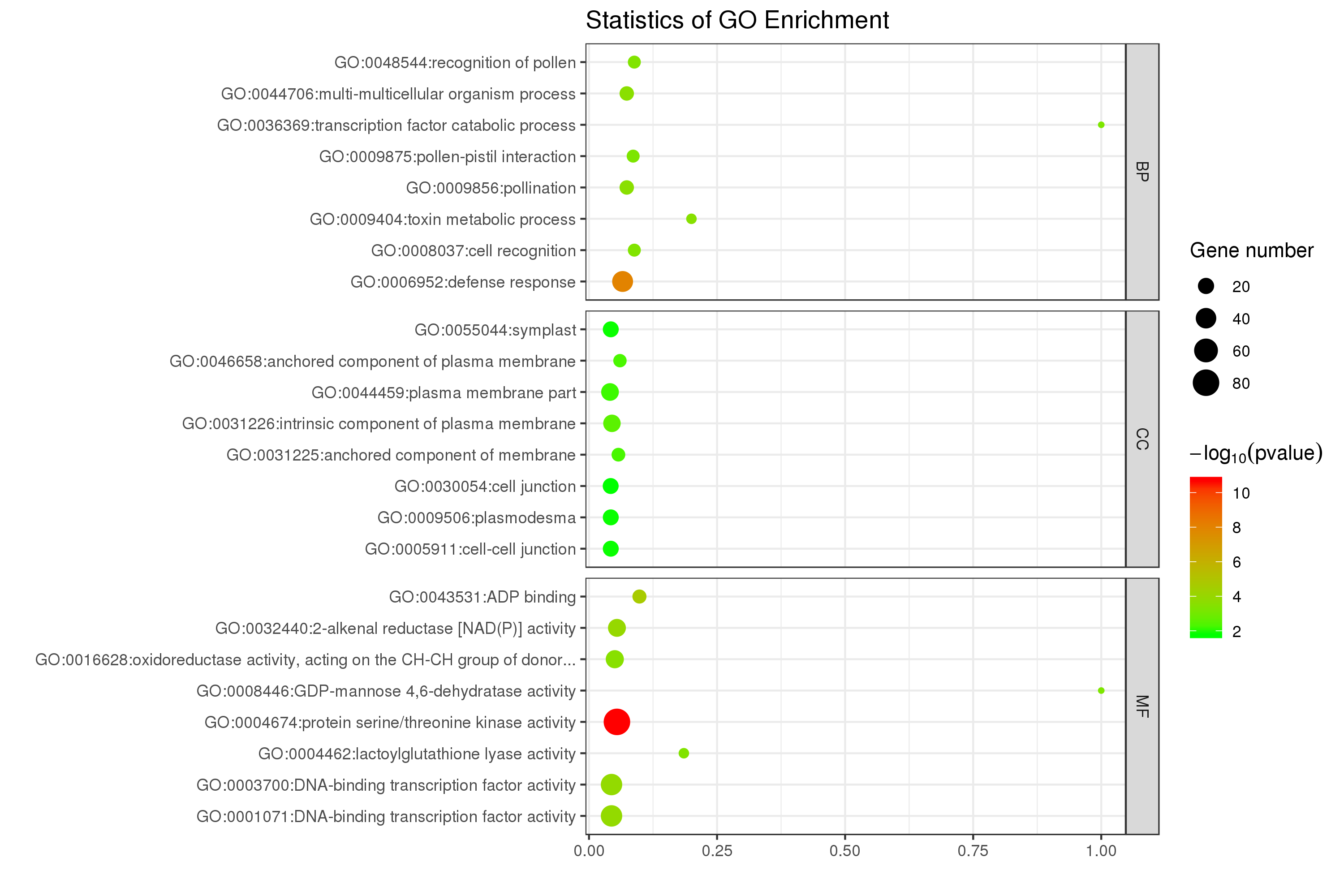

Supplement: Supplementary file 13 — Additional file 13: Figure S2. Bubble diagram showing the GO classification of differentially expressed transcripts between Z141 and NY-17 under DS or RD treatment. (a, b) GO terms of downregulated genes overlapping between Z141 and NY-17 under DS (a) or RD (b) treatment. (c-f) GO terms of genes up- (c, d) or downregulated (e, f) in Z141 or NY-17 under only DS. (g-j) GO terms of genes up- (g, h) or downregulated (i, j) in Z141 or NY-17 under only RD. [file 12864_2021_7416_MOESM13_ESM.zip › Supplementary Figure S2I.png]

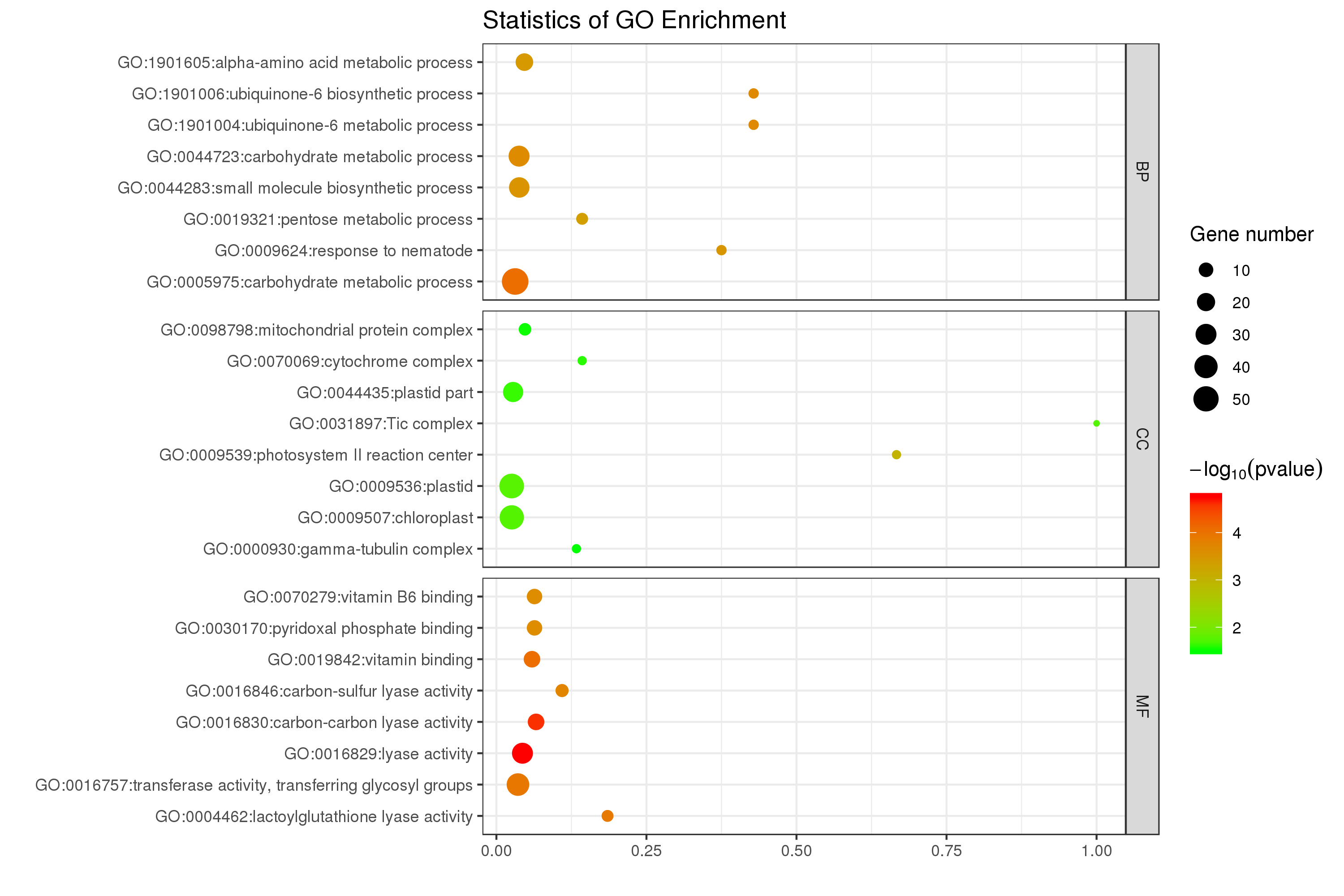

Supplement: Supplementary file 13 — Additional file 13: Figure S2. Bubble diagram showing the GO classification of differentially expressed transcripts between Z141 and NY-17 under DS or RD treatment. (a, b) GO terms of downregulated genes overlapping between Z141 and NY-17 under DS (a) or RD (b) treatment. (c-f) GO terms of genes up- (c, d) or downregulated (e, f) in Z141 or NY-17 under only DS. (g-j) GO terms of genes up- (g, h) or downregulated (i, j) in Z141 or NY-17 under only RD. [file 12864_2021_7416_MOESM13_ESM.zip › Supplementary Figure S2J.png]

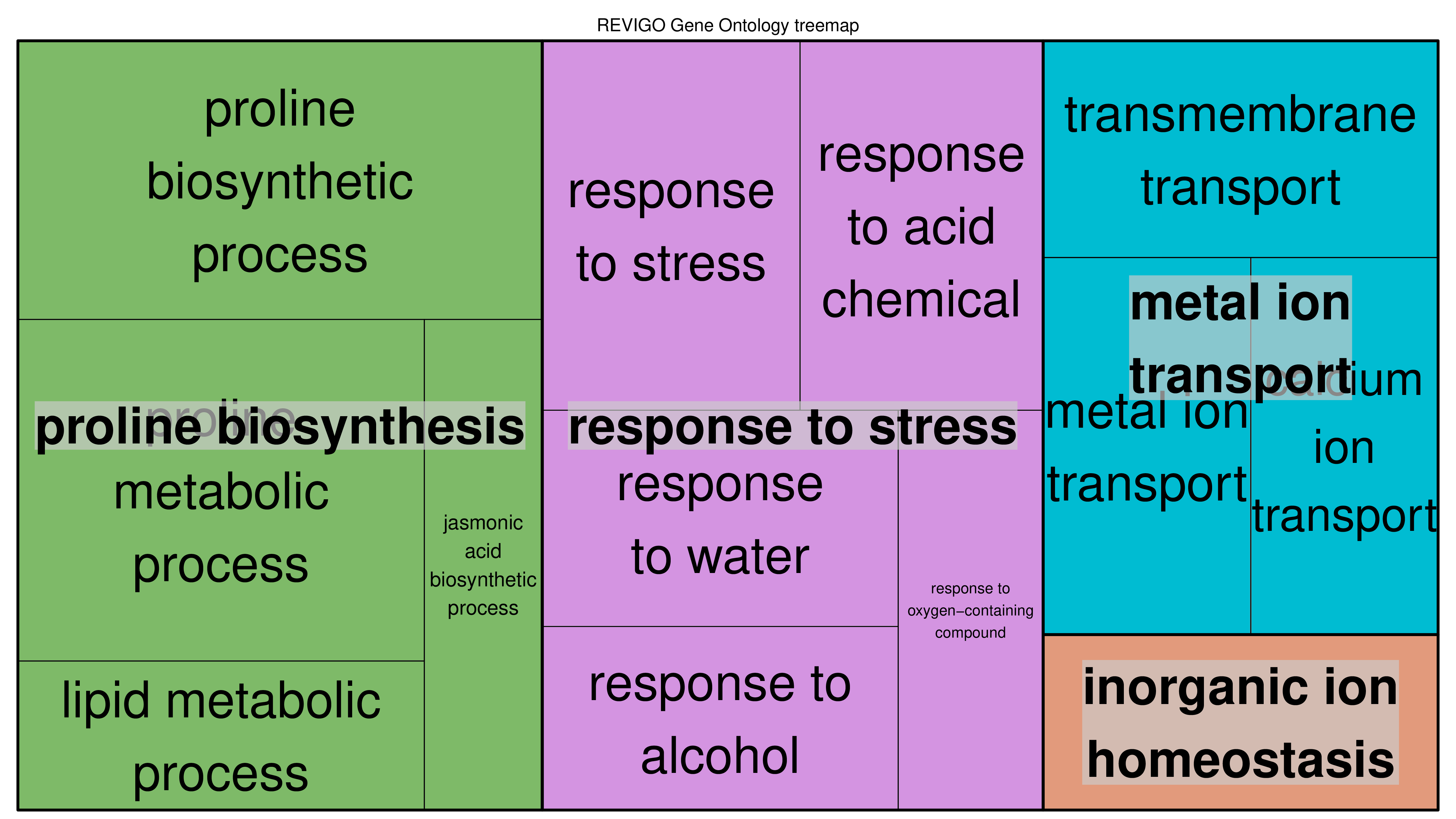

Supplement: Supplementary file 15 — Additional file 15: Figure S3. Tree diagram showing the REVIGO classification of up- or down-regulated differentially expressed transcripts in Z141 or NY-17 under DS or RD respectively. (a, b) The REVIGO classification of up- (a) and down-regulated (b) genes in Z141 under RD stress. (c, d) The REVIGO classification of up- (c) and down-regulated (d) genes in NY-17 under DS stress. (e, f) The REVIGO classification of up- (e) and down-regulated (f) genes in NY-17 under RD stress. [file 12864_2021_7416_MOESM15_ESM.zip › Supplementary Figure S3A.png]

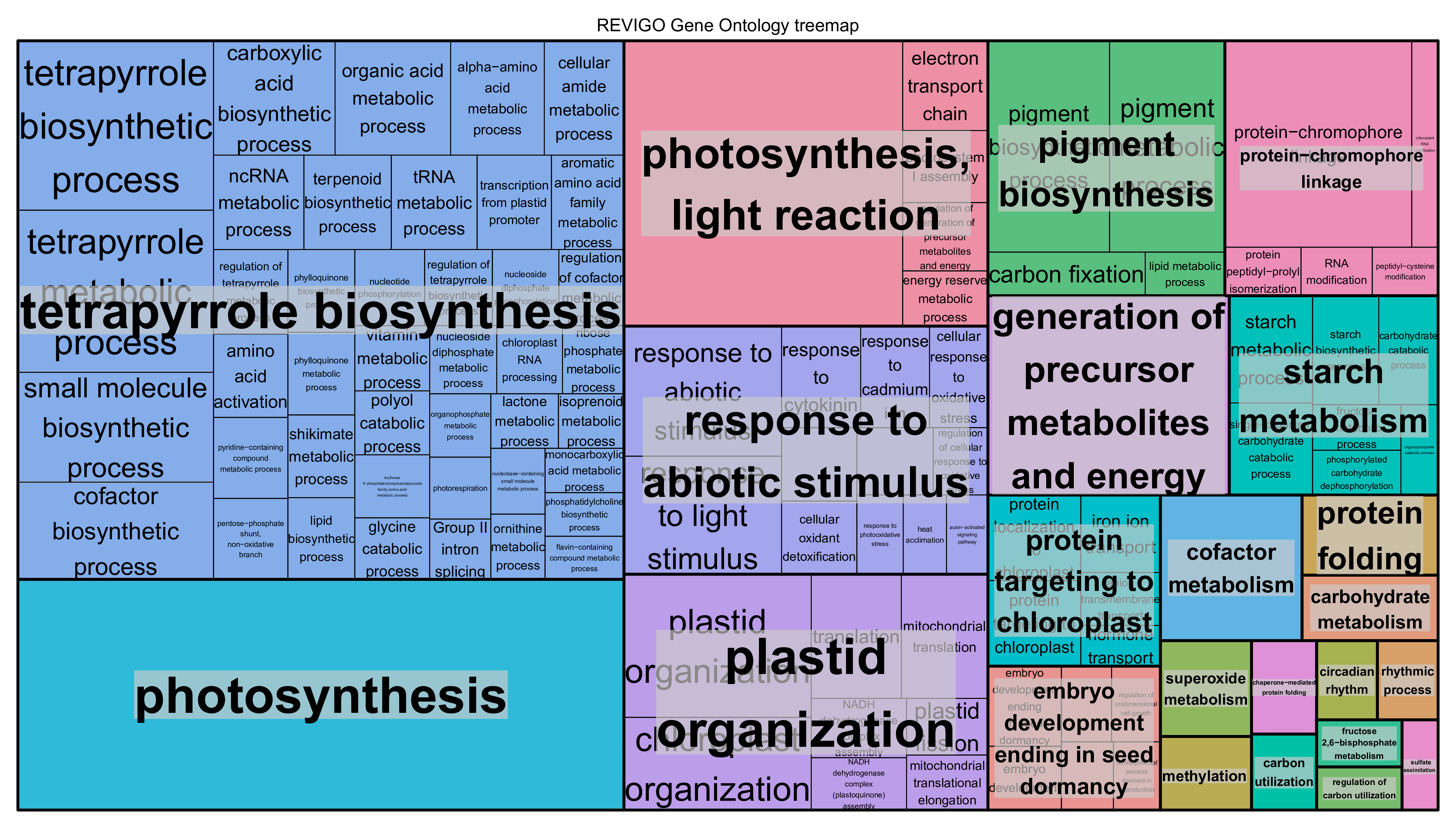

Supplement: Supplementary file 15 — Additional file 15: Figure S3. Tree diagram showing the REVIGO classification of up- or down-regulated differentially expressed transcripts in Z141 or NY-17 under DS or RD respectively. (a, b) The REVIGO classification of up- (a) and down-regulated (b) genes in Z141 under RD stress. (c, d) The REVIGO classification of up- (c) and down-regulated (d) genes in NY-17 under DS stress. (e, f) The REVIGO classification of up- (e) and down-regulated (f) genes in NY-17 under RD stress. [file 12864_2021_7416_MOESM15_ESM.zip › Supplementary Figure S3B.png]

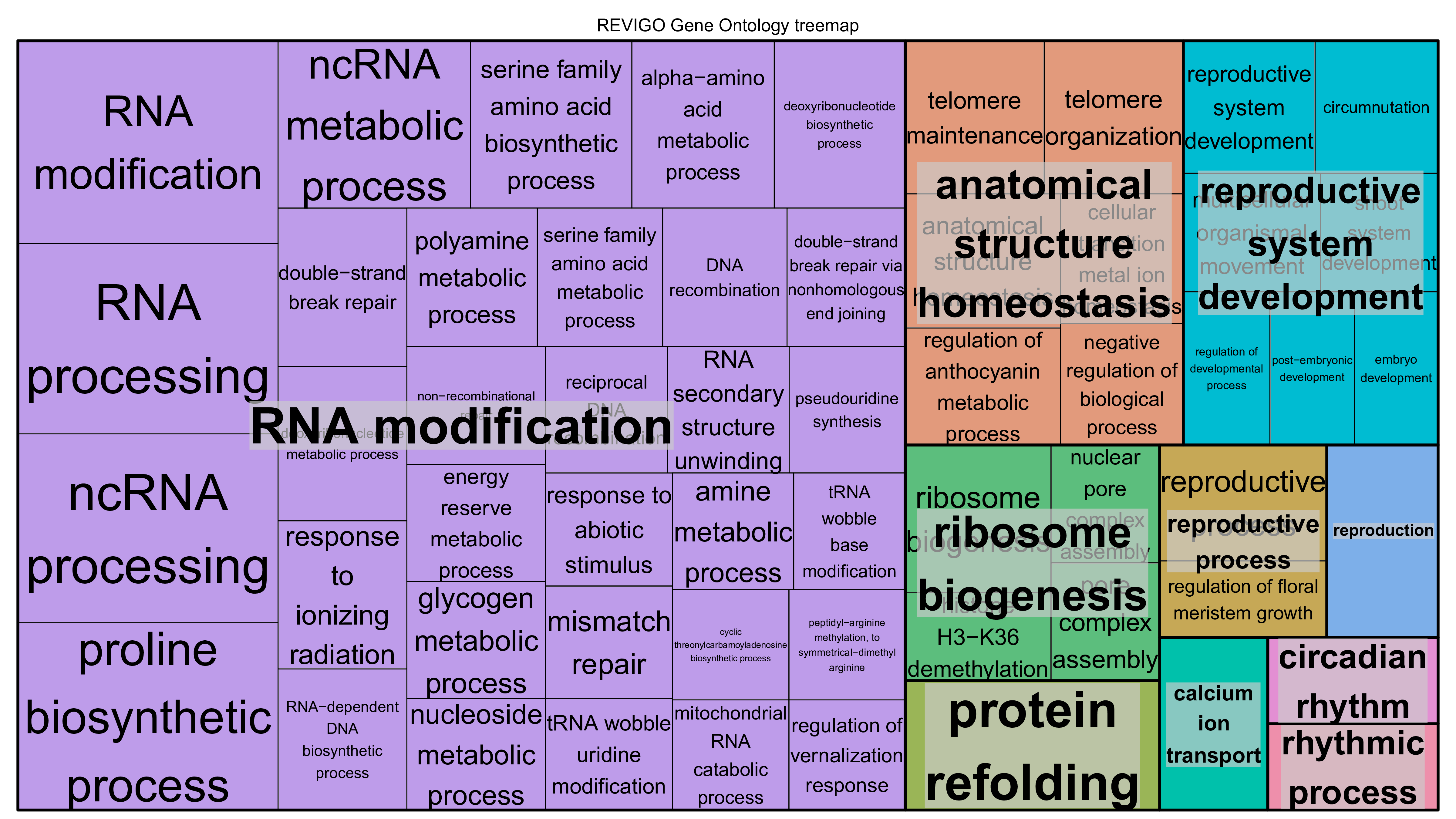

Supplement: Supplementary file 15 — Additional file 15: Figure S3. Tree diagram showing the REVIGO classification of up- or down-regulated differentially expressed transcripts in Z141 or NY-17 under DS or RD respectively. (a, b) The REVIGO classification of up- (a) and down-regulated (b) genes in Z141 under RD stress. (c, d) The REVIGO classification of up- (c) and down-regulated (d) genes in NY-17 under DS stress. (e, f) The REVIGO classification of up- (e) and down-regulated (f) genes in NY-17 under RD stress. [file 12864_2021_7416_MOESM15_ESM.zip › Supplementary Figure S3C.png]

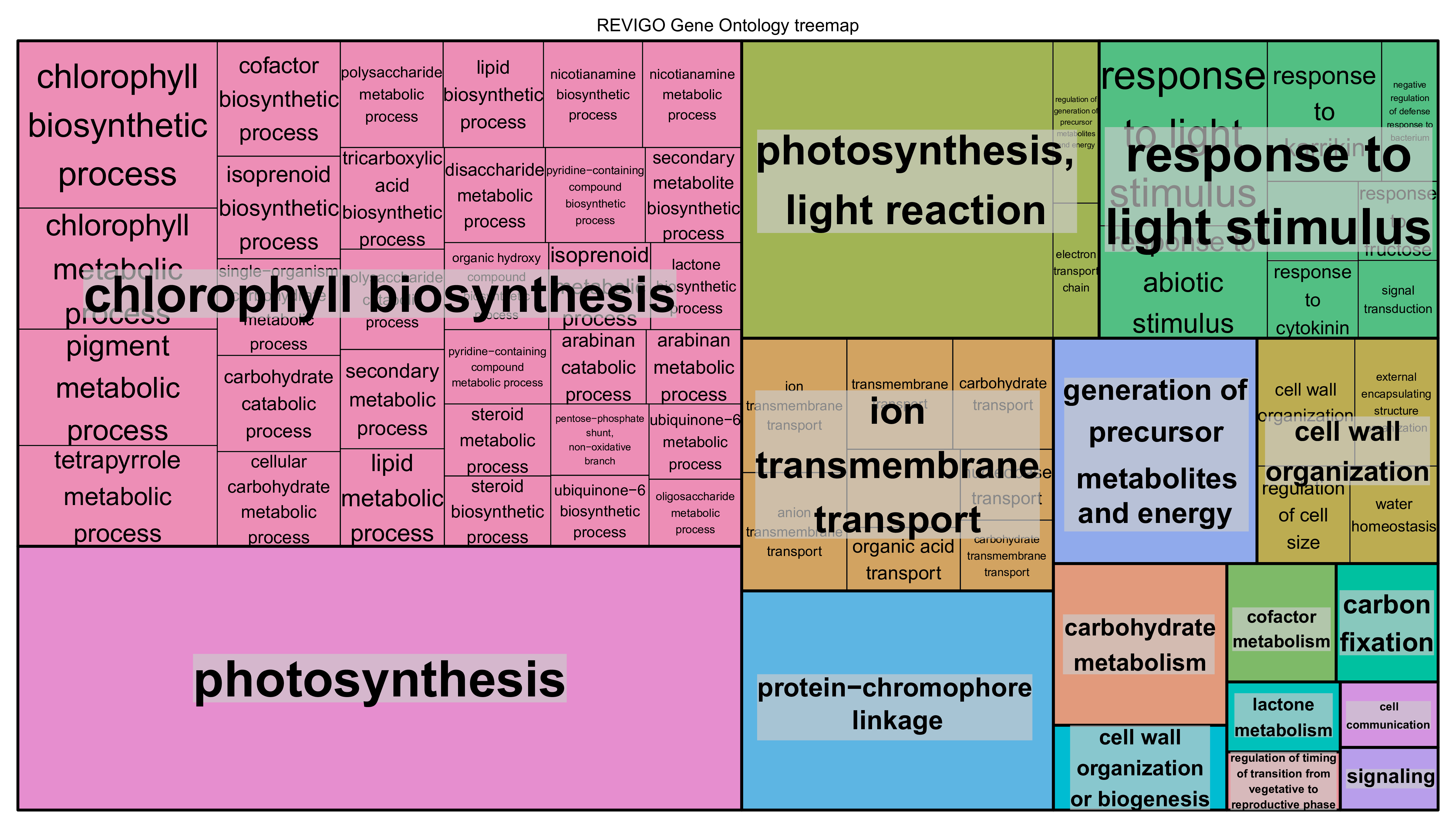

Supplement: Supplementary file 15 — Additional file 15: Figure S3. Tree diagram showing the REVIGO classification of up- or down-regulated differentially expressed transcripts in Z141 or NY-17 under DS or RD respectively. (a, b) The REVIGO classification of up- (a) and down-regulated (b) genes in Z141 under RD stress. (c, d) The REVIGO classification of up- (c) and down-regulated (d) genes in NY-17 under DS stress. (e, f) The REVIGO classification of up- (e) and down-regulated (f) genes in NY-17 under RD stress. [file 12864_2021_7416_MOESM15_ESM.zip › Supplementary Figure S3D.png]

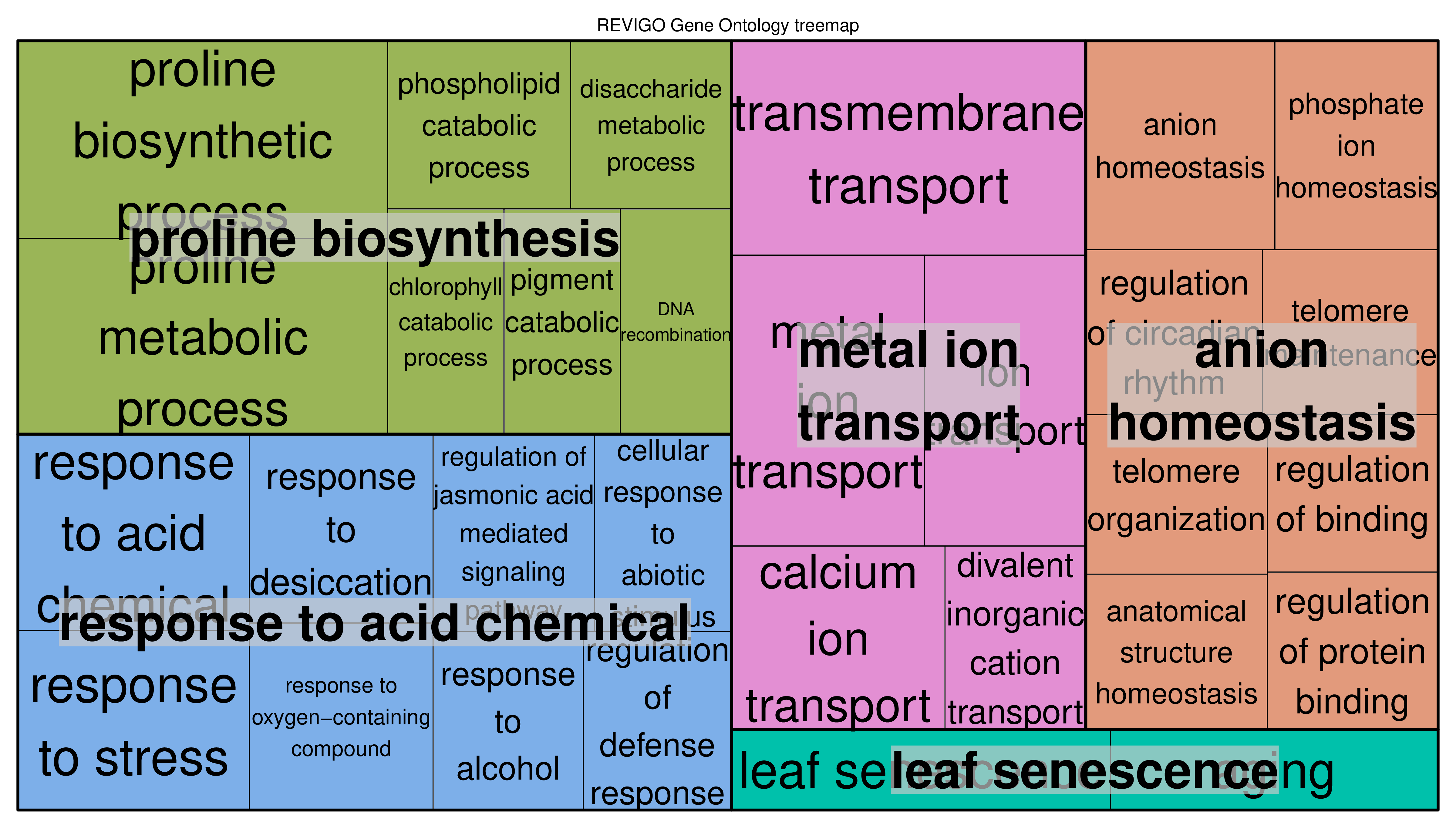

Supplement: Supplementary file 15 — Additional file 15: Figure S3. Tree diagram showing the REVIGO classification of up- or down-regulated differentially expressed transcripts in Z141 or NY-17 under DS or RD respectively. (a, b) The REVIGO classification of up- (a) and down-regulated (b) genes in Z141 under RD stress. (c, d) The REVIGO classification of up- (c) and down-regulated (d) genes in NY-17 under DS stress. (e, f) The REVIGO classification of up- (e) and down-regulated (f) genes in NY-17 under RD stress. [file 12864_2021_7416_MOESM15_ESM.zip › Supplementary Figure S3E.png]

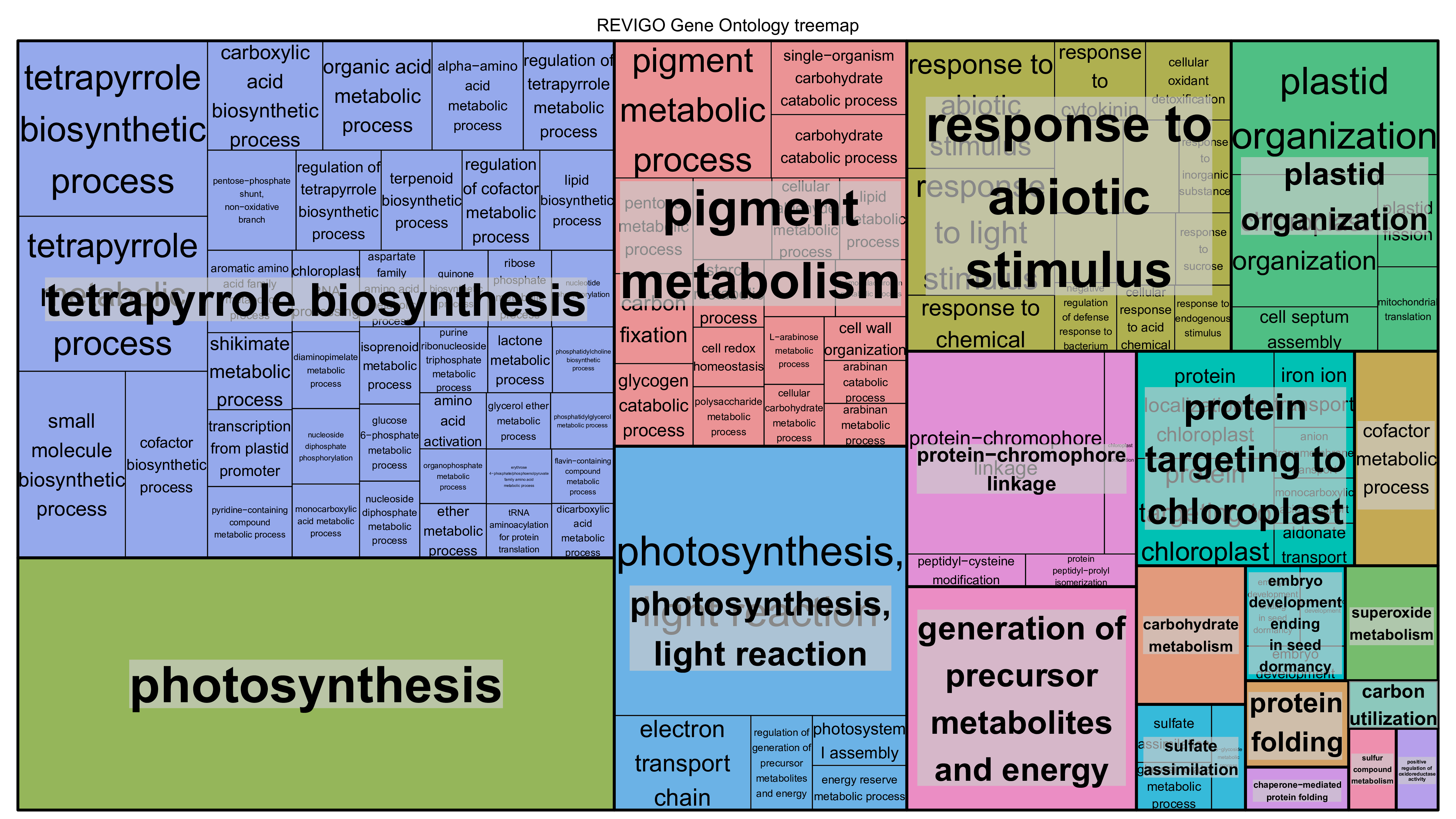

Supplement: Supplementary file 15 — Additional file 15: Figure S3. Tree diagram showing the REVIGO classification of up- or down-regulated differentially expressed transcripts in Z141 or NY-17 under DS or RD respectively. (a, b) The REVIGO classification of up- (a) and down-regulated (b) genes in Z141 under RD stress. (c, d) The REVIGO classification of up- (c) and down-regulated (d) genes in NY-17 under DS stress. (e, f) The REVIGO classification of up- (e) and down-regulated (f) genes in NY-17 under RD stress. [file 12864_2021_7416_MOESM15_ESM.zip › Supplementary Figure S3F.png]

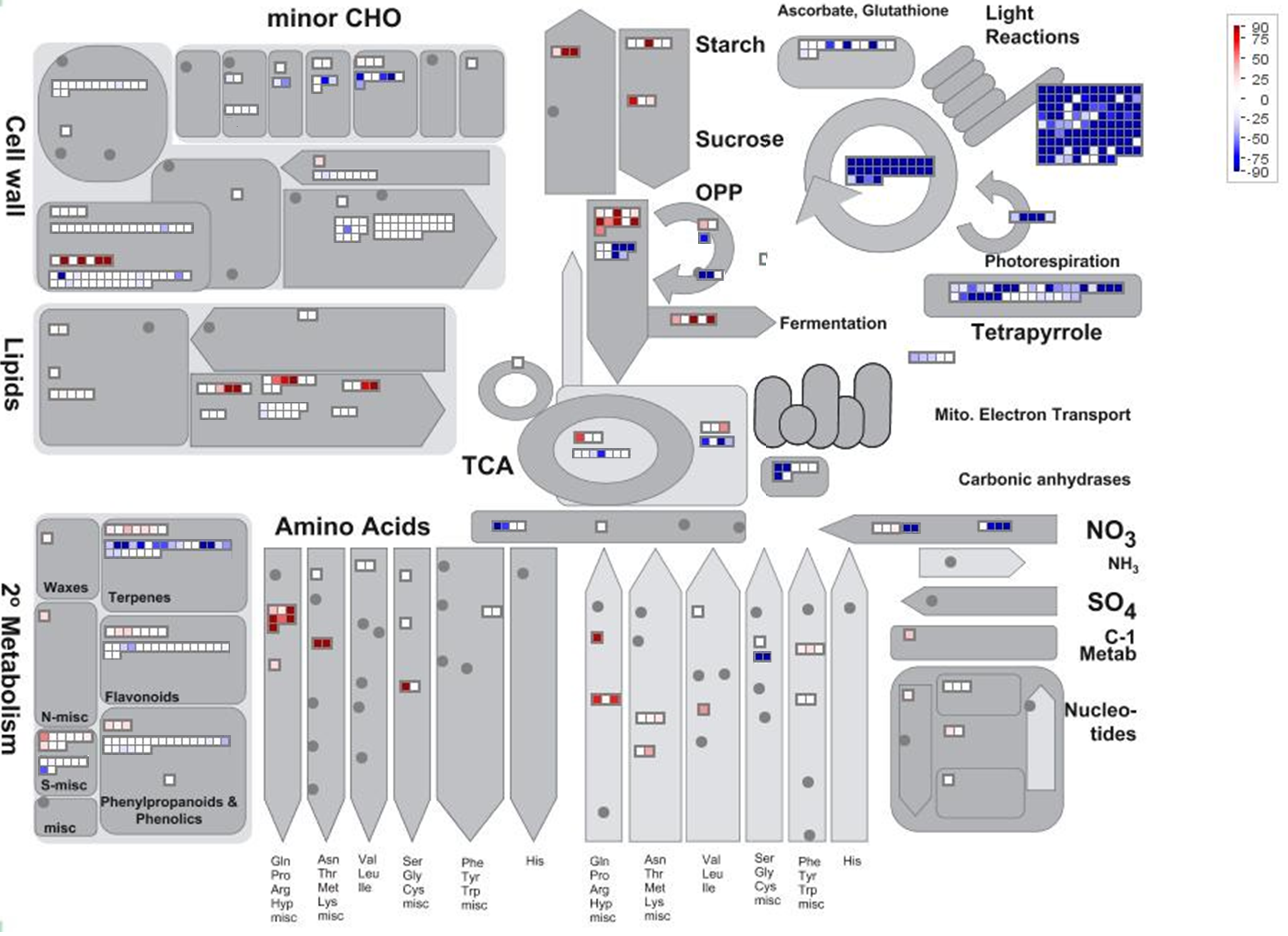

Supplement: Supplementary file 16 — Additional file 16: Figure S4. MapMan visualization of drought stress-responsive DEGs in Z141 (b) and NY-17 (a, c) under DS and RD stress, respectively. The up- and downregulated DEGs are represented in red and blue colour. The Colour brightness indicates the degree of difference, as shown in the scale on the right. [file 12864_2021_7416_MOESM16_ESM.zip › Supplementary Figure S4A.PNG]

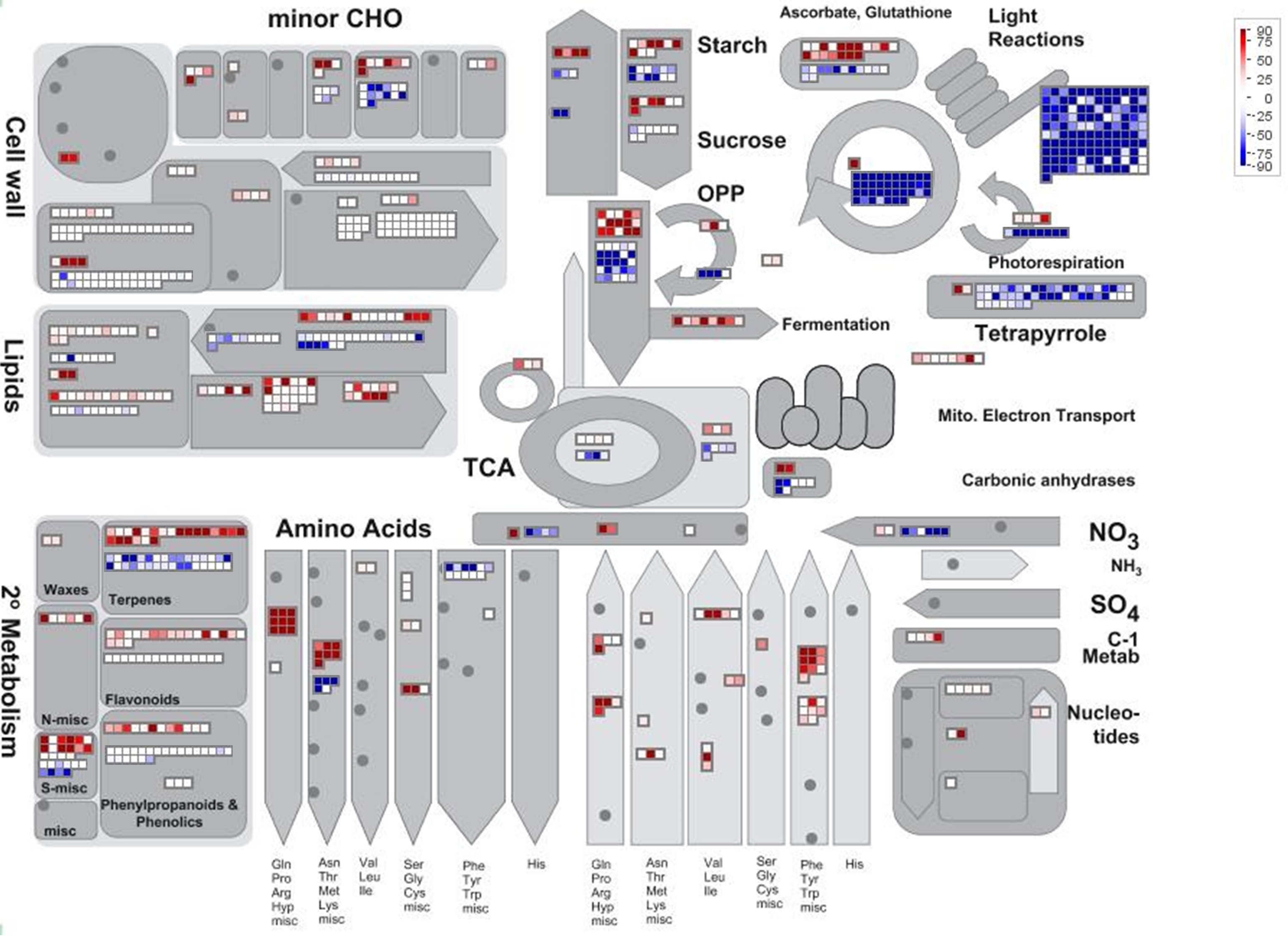

Supplement: Supplementary file 16 — Additional file 16: Figure S4. MapMan visualization of drought stress-responsive DEGs in Z141 (b) and NY-17 (a, c) under DS and RD stress, respectively. The up- and downregulated DEGs are represented in red and blue colour. The Colour brightness indicates the degree of difference, as shown in the scale on the right. [file 12864_2021_7416_MOESM16_ESM.zip › Supplementary Figure S4B.PNG]

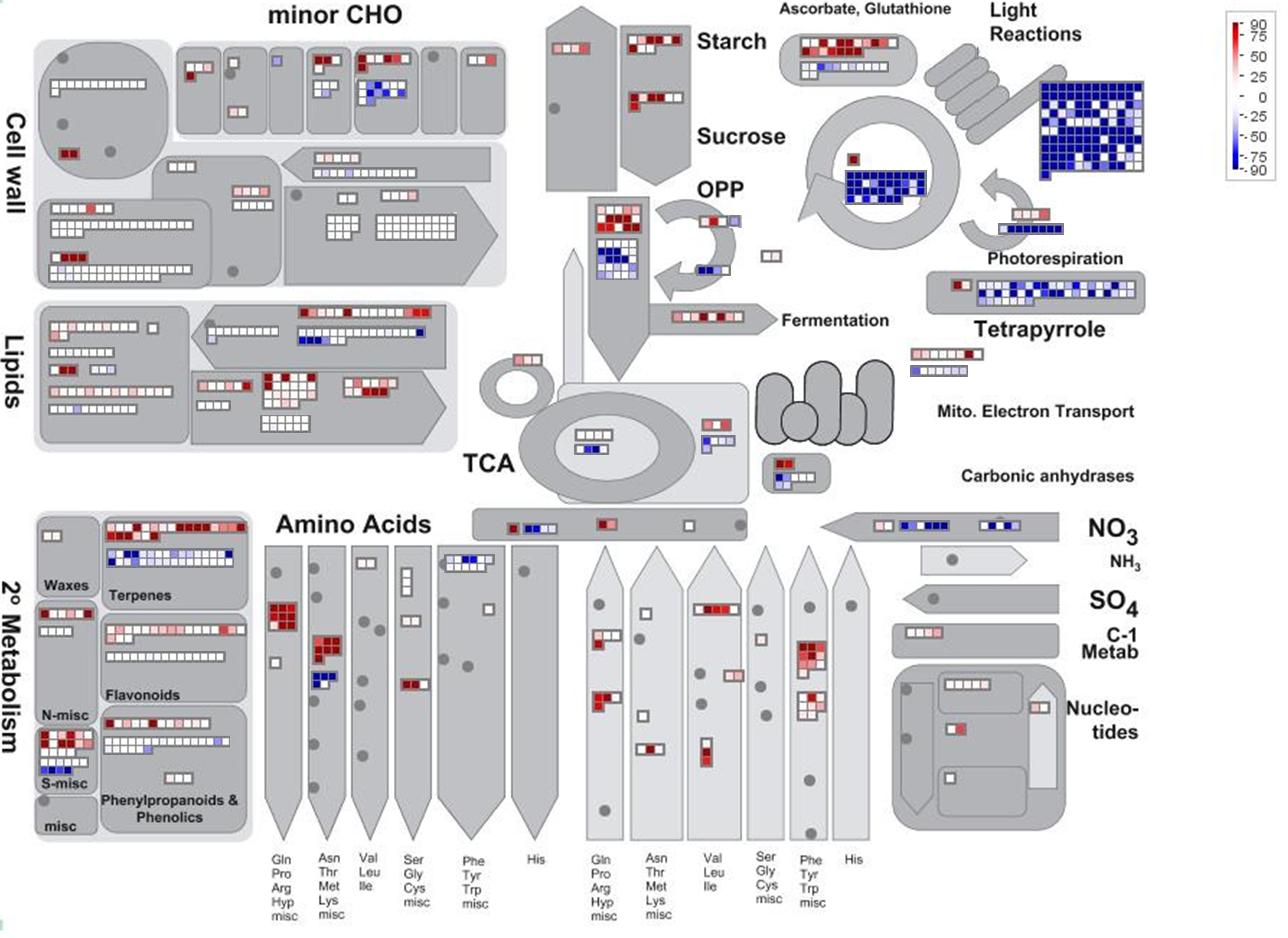

Supplement: Supplementary file 16 — Additional file 16: Figure S4. MapMan visualization of drought stress-responsive DEGs in Z141 (b) and NY-17 (a, c) under DS and RD stress, respectively. The up- and downregulated DEGs are represented in red and blue colour. The Colour brightness indicates the degree of difference, as shown in the scale on the right. [file 12864_2021_7416_MOESM16_ESM.zip › Supplementary Figure S4C.PNG]

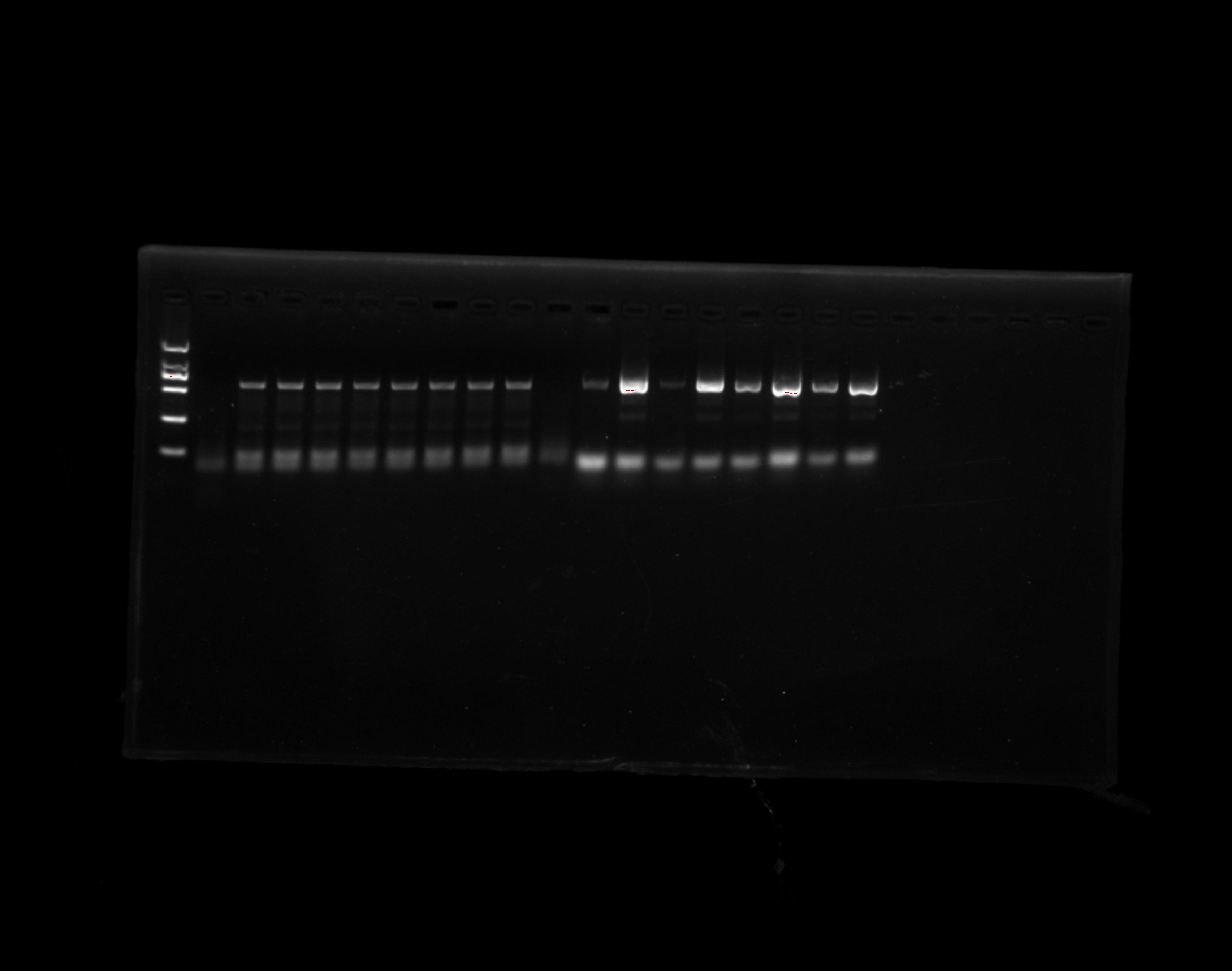

Supplement: Supplementary file 24 — Additional file 24: Figure S5. The original and full-length gel images of Fig. 10. (XLS 3462 kb) [file 12864_2021_7416_MOESM24_ESM.zip › Supplementary Figure S5a.tif]

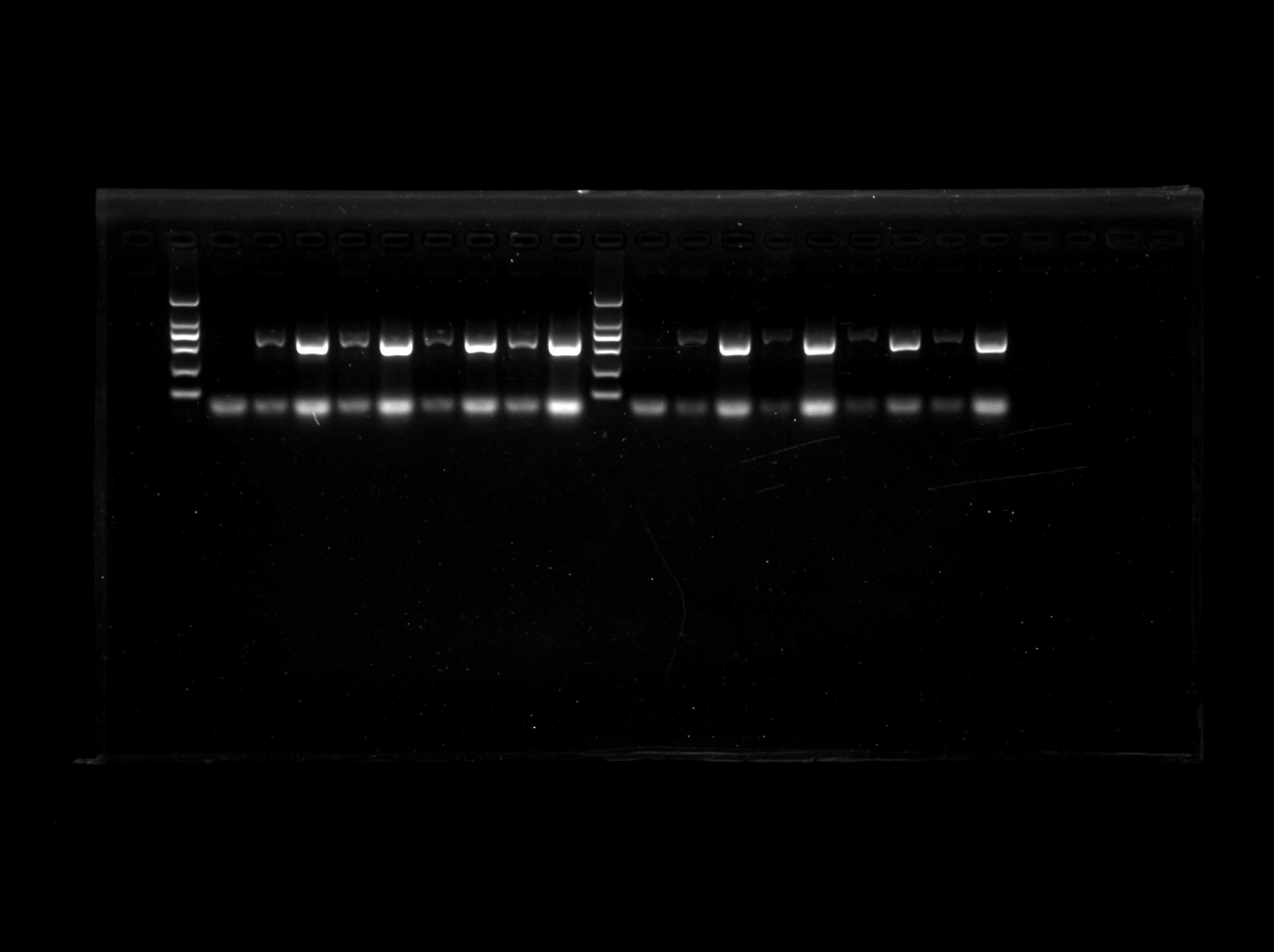

Supplement: Supplementary file 24 — Additional file 24: Figure S5. The original and full-length gel images of Fig. 10. (XLS 3462 kb) [file 12864_2021_7416_MOESM24_ESM.zip › Supplementary Figure S5b.tif]

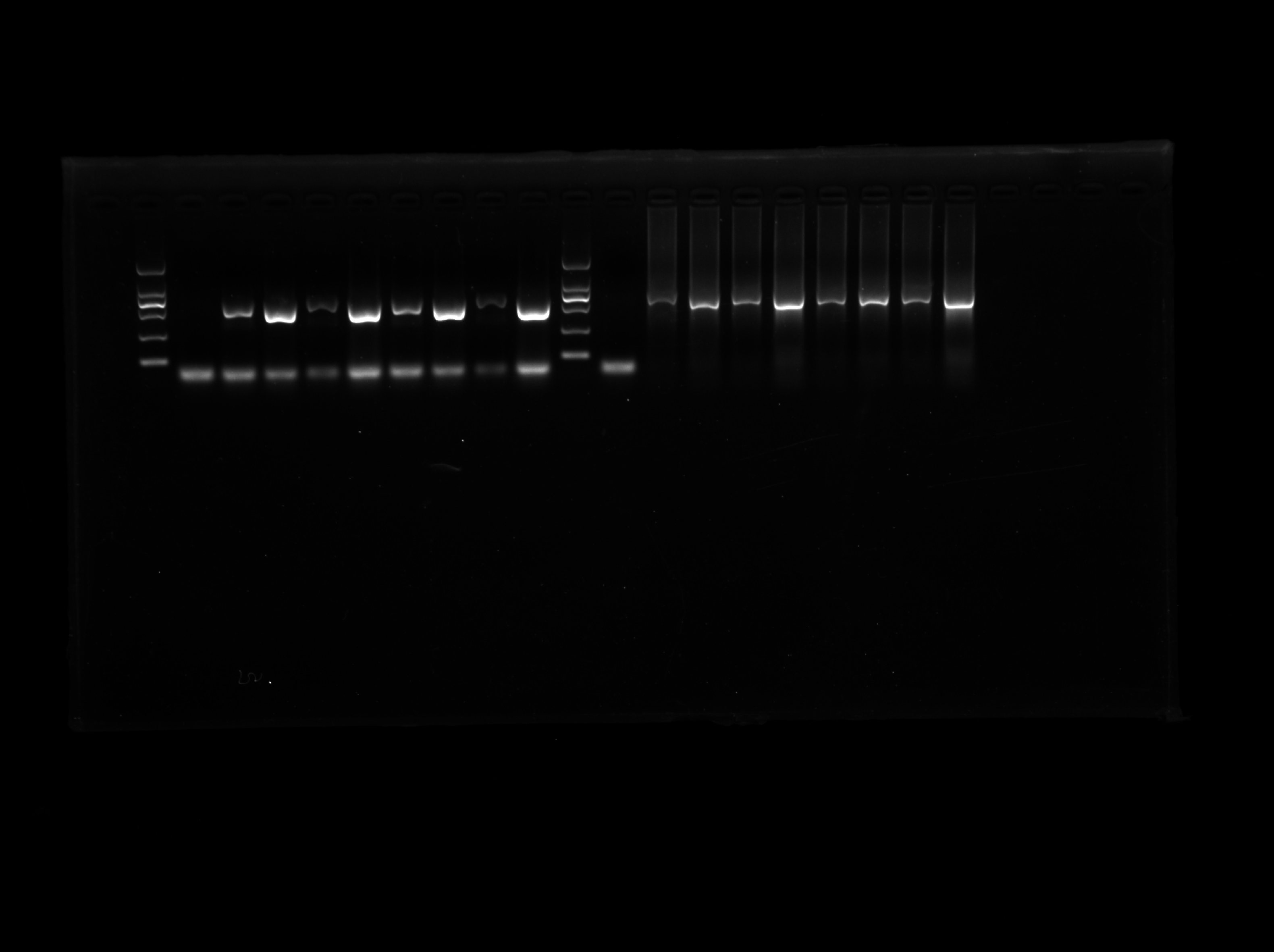

Supplement: Supplementary file 24 — Additional file 24: Figure S5. The original and full-length gel images of Fig. 10. (XLS 3462 kb) [file 12864_2021_7416_MOESM24_ESM.zip › Supplementary Figure S5c.tif]

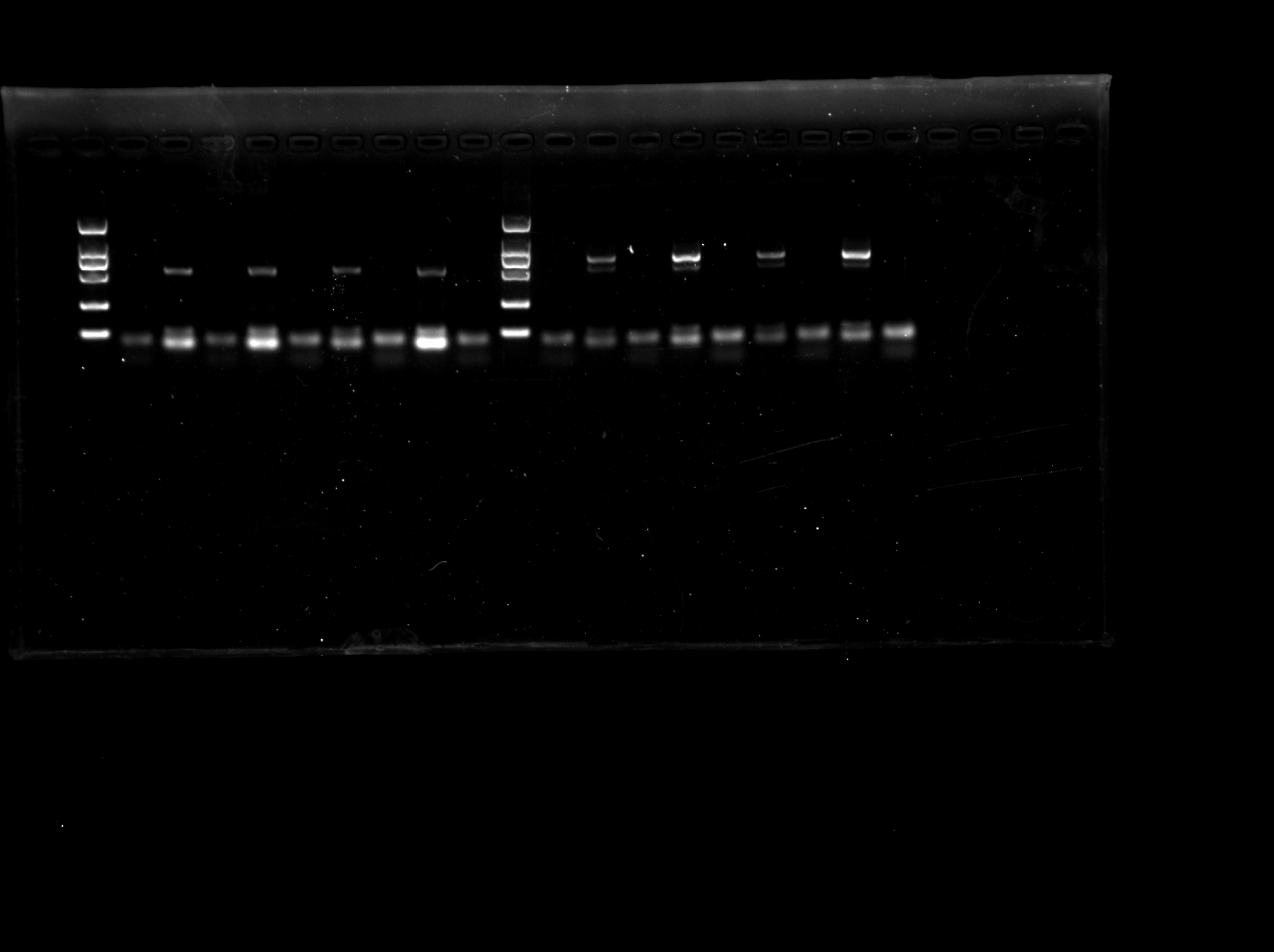

Supplement: Supplementary file 24 — Additional file 24: Figure S5. The original and full-length gel images of Fig. 10. (XLS 3462 kb) [file 12864_2021_7416_MOESM24_ESM.zip › Supplementary Figure S5d.tif]
