# Supplementary material for: Transcriptome analysis and molecular mechanism of linseed (Linum usitatissimum L.) drought tolerance under repeated drought using single-molecule long-read sequencing
Source: BMC Genomics. 2021 Feb 9;22:109. doi: 10.1186/s12864-021-07416-5 (PMC7871411; doi:10.1186/s12864-021-07416-5)
Supplement: Supplementary file 17 — Additional file 17: Table S13. Comparison of our predicated TFs with that released by PlantTFDB. [file 12864_2021_7416_MOESM17_ESM.docx]

Table S13 Comparison of our predicated TFs with that released by PlantTFDB

| **TF Family** | **Predicted in our analysis** | **PlantTFDB** |  | **TF Family** | **Predicted in our analysis** | **PlantTFDB** |
| --- | --- | --- | --- | --- | --- | --- |
| bHLH | 322 | 195 |  | CAMTA | 41 | 9 |
| MYB-related | 306 | 108 |  | NF-YC | 40 | 15 |
| C2H2 | 273 | 136 |  | AP2/ERF-AP2 | 35 | 35 |
| C3H | 273 | 61 |  | NF-YA | 35 | 11 |
| MYB | 269 | 206 |  | BBR-BPC | 29 | 8 |
| NAC | 264 | 191 |  | NF-X1 | 29 | 4 |
| bZIP | 245 | 107 |  | CPP | 28 | 9 |
| AP2/ERF-ERF | 235 | 193 |  | GARP-ARR-B | 27 | 15 |
| B3 | 180 | 107 |  | BES1 | 26 | 12 |
| WRKY | 167 | 108 |  | C2C2-YABBY | 24 | 15 |
| GRAS | 166 | 119 |  | E2F-DP | 21 | 14 |
| GARP-G2-like | 165 | 65 |  | GRF | 21 | 16 |
| B3-ARF | 143 | 34 |  | HB-WOX | 21 | 18 |
| HB-HD-ZIP | 111 | 62 |  | C2C2-LSD | 20 | 9 |
| HB-other | 103 | 23 |  | GeBP | 19 | 12 |
| SBP | 96 | 29 |  | CO-like | 17 | 12 |
| MADS-M-type | 90 | 67 |  | Whirly | 16 | 4 |
| Trihelix | 88 | 54 |  | zf-HD | 15 | 15 |
| MADS-MIKC | 69 | 47 |  | VOZ | 14 | 4 |
| HSF | 64 | 40 |  | DBB | 13 | 9 |
| C2C2-Dof | 56 | 43 |  | SRS | 12 | 13 |
| C2C2-GATA | 56 | 35 |  | AP2/ERF-RAV | 9 | 3 |
| FAR1 | 50 | 6 |  | EIL | 8 | 4 |
| NF-YB | 46 | 32 |  | HB-PHD | 4 | 3 |
| TCP | 45 | 23 |  | S1Fa-like | 4 | 4 |
