# Supplementary material for: Transcriptome analysis and molecular mechanism of linseed (Linum usitatissimum L.) drought tolerance under repeated drought using single-molecule long-read sequencing
Source: BMC Genomics. 2021 Feb 9;22:109. doi: 10.1186/s12864-021-07416-5 (PMC7871411; doi:10.1186/s12864-021-07416-5)
Supplement: Supplementary file 23 — Additional file 23: Table S19. Primers designed for RT-PCR validation. [file 12864_2021_7416_MOESM23_ESM.docx]

Table S19. Primers designed for RT-PCR validation

| **Gene ID** | **Primer** | **Sequence (5’-3’)** | **TM (℃)** | **Length (hp)** |
| --- | --- | --- | --- | --- |
| Lus10014603 | F | GCTGCACATTTGAAGGGTGG | 57.5 | 578 |
|  | R | ATTCCAGCCTTGGCATCGAA | 57.2 |  |
| Lus10021585 | F | GAACATCTCGCGTCTGCAAC | 57.0 | 593 |
|  | R | GCTCATCAACCTAGCCTGCA | 57.8 |  |
| Lus10012962 | F | GGTGGCCAATTTCTGCAAGG | 57.6 | 514 |
|  | R | TGTCATGACATCCCACAGCC | 57.8 |  |
| Lus10001832 | F | CAGTGGTGGAGCAGTCTCAG | 58.3 | 532 |
|  | R | TCCCCTATCCGGTTTGCCTA | 58.1 |  |
| Lus10004697 | F | TCCTAGAGGCAGCAACAAGC | 57.7 | 562 |
|  | R | ATCCACTACTTGCCCACTGC | 57.8 |  |
| Lus10001016 | F | TTCCTGTTCTGGGTCATGCC | 57.8 | 572 |
|  | R | GCCTCTCAATATCCACCGGG | 58.3 |  |
| Lus10038490 | F | CAGGTCTCATCAGGAAGCCC | 58.3 | 521 |
|  | R | CAGGCATTGTTGTTGACCGG | 57.4 |  |
| Lus10027966 | F | CAGCTATGGGGAAGTAGCCG | 58.3 | 539 |
|  | R | GGGTCATCAGCCAATCCCAA | 57.9 |  |

Table S10. The list of Z141 and NY-17 DEGs under DS or RD

Table S14. Detail lists of 15 clusters of differentially expressed transcription factors.

Table S17. All sample FPKM sheet

Table S18. All sample reads count sheet
